# Endothelial and hematopoietic hPSCs differentiation via a hematoendothelial progenitor

**DOI:** 10.1186/s13287-022-02925-w

**Published:** 2022-06-17

**Authors:** Alejandra Vargas-Valderrama, Anne-Charlotte Ponsen, Morgane Le Gall, Denis Clay, Sébastien Jacques, Tudor Manoliu, Valérie Rouffiac, Karine Ser-le-Roux, Cyril Quivoron, Fawzia Louache, Georges Uzan, Maria-Teresa Mitjavila-Garcia, Estelle Oberlin, Hind Guenou

**Affiliations:** 1 INSERM UMRS-MD 1197, Hôpital Paul Brousse, Université Paris-Saclay, 94807 Villejuif, France; 2 Plateforme Protéomique 3P5-Proteom’IC, Institut Cochin, INSERM U1016, CNRS UMR8104, Université de Paris, 75014 Paris, France; 3 INSERM UMS-44, Hôpital Paul Brousse, Université Paris Sud-Université Paris-Saclay, 94807 Villejuif, France; 4 Plateforme de Génomique- GENOM’IC, Institut Cochin, INSERM U1016, CNRS UMR8104, Université de Paris, 75014 Paris, France; 5 Plate-forme Imagerie et Cytométrie, UMS AMMICa, Gustave Roussy, Université Paris-Saclay, 94805 Villejuif, France; 6 INSERM, UMS AMMICa, Plate-forme d’Evaluation Préclinique, Gustave Roussy, 94807 Villejuif, France; 7 Laboratoire d’Hématologie Translationnelle, Gustave Roussy, 94805 Villejuif, France; 8 Université d’Evry-Val-d’Essonne, Université Paris-Saclay, 91000 Evry, France

**Keywords:** Hemogenic endothelium, Endothelium, Hematopoietic cells, hPSC, hESCs, hiPSC, Endothelial differentiation, Hematopoietic differentiation

## Abstract

**Background:**

hPSC-derived endothelial and hematopoietic cells (ECs and HCs) are an interesting source of cells for tissue engineering. Despite their close spatial and temporal embryonic development, current hPSC differentiation protocols are specialized in only one of these lineages. In this study, we generated a hematoendothelial population that could be further differentiated in vitro to both lineages.

**Methods:**

Two hESCs and one hiPSC lines were differentiated into a hematoendothelial population, hPSC-ECs and blast colonies (hPSC-BCs) via CD144^+^-embryoid bodies (hPSC-EBs). hPSC-ECs were characterized by endothelial colony-forming assay, LDL uptake assay, endothelial activation by TNF-α, nitric oxide detection and Matrigel-based tube formation. Hematopoietic colony-forming cell assay was performed from hPSC-BCs. Interestingly, we identified a hPSC-BC population characterized by the expression of both CD144 and CD45. hPSC-ECs and hPSC-BCs were analyzed by flow cytometry and RT-qPCR; in vivo experiments have been realized by ischemic tissue injury model on a mouse dorsal skinfold chamber and hematopoietic reconstitution in irradiated immunosuppressed mouse from hPSC-ECs and hPSC-EB-CD144^+^, respectively. Transcriptomic analyses were performed to confirm the endothelial and hematopoietic identity of hESC-derived cell populations by comparing them against undifferentiated hESC, among each other’s (*e.g.* hPSC-ECs vs. hPSC-EB-CD144^+^) and against human embryonic liver (EL) endothelial, hematoendothelial and hematopoietic cell subpopulations.

**Results:**

A hematoendothelial population was obtained after 84 h of hPSC-EBs formation under serum-free conditions and isolated based on CD144 expression. Intrafemorally injection of hPSC-EB-CD144^+^ contributed to the generation of CD45^+^ human cells in immunodeficient mice suggesting the existence of hemogenic ECs within hPSC-EB-CD144^+^. Endothelial differentiation of hPSC-EB-CD144^+^ yields a population of > 95% functional ECs in vitro. hPSC-ECs derived through this protocol participated at the formation of new vessels in vivo in a mouse ischemia model. In vitro, hematopoietic differentiation of hPSC-EB-CD144^+^ generated an intermediate population of > 90% CD43^+^ hPSC-BCs capable to generate myeloid and erythroid colonies. Finally, the transcriptomic analyses confirmed the hematoendothelial, endothelial and hematopoietic identity of hPSC-EB-CD144^+^, hPSC-ECs and hPSC-BCs, respectively, and the similarities between hPSC-BC-CD144^+^CD45^+^, a subpopulation of hPSC-BCs, and human EL hematopoietic stem cells/hematopoietic progenitors.

**Conclusion:**

The present work reports a hPSC differentiation protocol into functional hematopoietic and endothelial cells through a hematoendothelial population. Both lineages were proven to display characteristics of physiological human cells, and therefore, they represent an interesting rapid source of cells for future cell therapy and tissue engineering.

**Supplementary Information:**

The online version contains supplementary material available at 10.1186/s13287-022-02925-w.

## Introduction

The rise of applied biology areas such as tissue engineering and biomedicine has highlighted the importance of endothelial (ECs) and hematopoietic cells (HCs) in building up the complexity of in vitro biological systems. ECs line the walls of blood and lymphatic vessels, assuring the transport of oxygen, nutrients, and cells throughout the human body. HCs encompass a large number of cells ranging from red blood to immune cells. The discovery of human embryonic stem cells (hESCs) [1] and later, the reprogramming of somatic cells into human-induced pluripotent stem cells (hiPSCs) [2, 3], opened the possibility to generate in vitro large numbers of these and other cell lineages sharing the same genetic background. However, the differentiation of human pluripotent stem cells (hPSCs) into ECs and HCs has been hampered by the limited knowledge on the human embryonic development of both lineages.

Several studies, performed in vitro and in vivo in animal models such as birds, fish and mice, have demonstrated the embryonic endothelial origin of adult HSCs [4, 5]. ECs emerge early during embryonic development from extra- and intraembryonic mesodermal progenitors. They organize into networks giving place to vascular plexuses and major blood vessels [6]. A subpopulation of ECs, known as hemogenic endothelium, located principally at the aorta–gonad–mesonephros (AGM) region but also in the placenta, and the yolk sac has been described to undergo endothelial-to-hematopoietic transition to give rise to primitive and definitive hematopoiesis [7, 8]. Hematopoietic progenitors (HP), arising from the hemogenic endothelium and known as pre-hematopoietic stem cells (pre-HSC), display a dual endo-hematopoietic phenotype characterized by the expression of the endothelial-specific junctional vascular endothelial cadherin (Cdh5, CD144) and the presence of the pan-leukocyte antigen CD45 [9–13]. They migrate to the embryonic liver to expand and maturate before colonizing the bone marrow (BM), the definitive hematopoietic reservoir in adult organisms [14–16]. There, ECs keep playing an important role in controlling proliferation and differentiation of HSCs [17].

Inspired by human embryonic development, several research groups have developed protocols to differentiate hPSCs into HPs through the derivation of an in vitro hemogenic endothelium [5, 18–21]. Other groups have reported efficient differentiation of hPSCs into functional ECs [22–33]. In both cases, protocols require the selection of endothelial progenitors at around day 6 of differentiation. Today only few studies have simultaneously generated both ECs and HPs from hPSCs [34–36]. The final hPSC-derived population is often as a mix of both lineages, without in vivo validation of independent cell types. Interestingly, despite all the efforts made in improving the current differentiation protocols, no functional nor transcriptomic characterization of these hPSC-derived lineages compared to human embryonic physiological populations has been done.

ECs and HCs simultaneous differentiation may be beneficial for their maturation and further biomedical applications. In this study, we generated a well-characterized population of hematoendothelial progenitors from hPSCs after only 84 h of differentiation in a serum-free medium without feeder. This population was proven to contain hemogenic ECs that generated HCs in vitro through the formation of blast colonies (hPSC-BCs) and in vivo in irradiated immunodeficient mice. Similarly, a pure population (> 90%) of hPSC-ECs was obtained from these hematoendothelial progenitors and showed a functional endothelial phenotype in vitro and in vivo in an ischemia model on a mouse dorsal skinfold chamber.

Finally, we analyzed the gene expression profiles of hPSC-derived populations (*i.e.,* EB-CD144+, unsorted-BC, BC-CD144^+^CD45^+^ and ECs) by Affymetrix GeneChip microarrays and compared them to undifferentiated hPSCs, to differentiated-hPSCs themselves and to physiological subpopulations isolated from human embryonic livers (EL). Thanks to this study, we confirmed their molecular identity and their similarities to their respective physiological counterparts. In conclusion, herein we present a protocol to differentiate hPSCs into ECs and HPs through an early hematoendothelial population and define a developmental hierarchy among hPSC-derived populations that mimics the ontogeny of the human hematopoietic system.

## Materials and methods

### Human pluripotent stem cells

hESCs, SA01 (Cellartis AB, Sweden) and H1 (WiCell Research Institute, USA), were provided by the INSERM cell bank «*ESteam Paris Sud—Plate-forme cellules souches*» (INSERM U935) according to the agreement n° RE14_007R of the French Biomedical Agency*.* The hiPSC line A29 was derived, characterized, and provided by the team INSERM U1193 led by Dr. Dubart-Kupperschmitt in the frame of the iLite RHU (ANR-16-RHUS-0005) [37]. Mitomycin-treated mouse embryonic fibroblasts were used as feeder cells for all hPSCs in knockout DMEM medium (Gibco) supplemented with 20% knockout serum replacement (Gibco), 1% penicillin/streptomycin, 1 mM l-glutamine, 1% non-essential amino acids, 0.1 mM β-Mercaptoethanol (all from Gibco) and 5 ng/mL basic fibroblast growth factor (bFGF, Miltenyi) at 37 °C and 5% CO_2_. The medium was daily replaced by fresh medium, and cells were enzymatically passaged upon confluency, by using collagenase type IV at 1 mg/mL (Gibco).

### hPSCs differentiation into a hematoendothelial population, hPSC-ECs and hPSC-BCs

Confluent hPSCs, between passages 52 to 100, were enzymatically passaged as described in the preceding paragraph. Small cell clumps were cultured in embryoid body (hPSC-EB) basal medium (Stemline II Hematopoietic Stem Cell Expansion Medium (Sigma-Aldrich) supplied with 1% streptomycin, 1 mM de L-glutamine) supplemented with 50 ng/mL VEGF-A_165_ (Peprotech), 50 ng/mL BMP-4 and 6 µM CHIR99021 (StemCell Technologies) in low-attachment culture plates (NUNC, ThermoFisher) at a cellular density equivalent to 3.5 × 10^4^ cells/ml. After 48 h, EB basal medium was refreshed and supplemented with 50 ng/mL of VEGF-A_165_ and BMP-4, and 20 ng/mL of SCF, Flt-3L and TPO (all from Peprotech). At 84 h of EB differentiation, EBs were recovered and dissociated enzymatically with Accutase (Corning). CD144^+−^cells were isolated from hPSC-EBs by magnetic-based cell sorting (MACS) using microbeads conjugated with anti-hCD144 antibodies (Miltenyi) on MS sorting columns (Miltenyi) according to the manufacturer's instructions.

CD144^+^- and CD144^−^-sorted cells from 84 h-hPSC-EBs (Table 1) were plated on fibronectin (Sigma-Aldrich) pre-coated well plates or Lab-Tek chamber slides (ThermoFisher) at a density of 15,000 cells/cm^2^ in EC culture medium: STEMdiff^™^ APEL^™^2 Medium (StemCell Technologies) supplied with 5% FBS (Lonza), 1 mM de l-glutamine, 50 ng/mL VEGF-A_165_ and 10 ng/mL bFGF. 10 µM of DAPT (Sigma Aldrich) was added to the EC culture medium for artery/vein differentiation experiments. hPSC-ECs (Table 1) were cultured at 37 °C and 5% CO_2_. Medium was replaced every other day. Confluent ECs was passaged enzymatically with Accutase (approximately every 4–6 days).

**Table 1:** Undifferentiated and differentiated hPSC populations

Populations	Abbreviations/identifications used in the text
Undifferentiated hPSCs	
hESCs	H1 and SA01 cell lines
hiPSCs	A29
Differentiated hPSCs	
Non-sorted EBs at 84 h of differentiation	hPSC-EBs
Sorted EBs based on the expression of CD144	hPSC-EB-CD144^+^
ECs derived from hPSC-EB-CD144^+^	hPSC-ECs
BCs derived from hPSC-EB-CD144^+^	hPSC-BCs
Sorted hPSC-BCs based on the expression of CD144 and CD45	hPSC-BC-CD144^+^CD45^+^

In order to obtain hPSC-BCs (Table 1), 50,000 CD144^+^ and hPSC-EB-CD144^−^ cells/mL were cultured for 6 days in a serum-free enriched methylcellulose (StemCell Technologies) and EB basal medium supplied with 50 ng/mL of VEGF-A_165_, BMP-4, TPO and Flt-3L, 20 ng/mL of bFGF and 3 units/mL of EPO in low-attachment culture plates.

### Endothelial colony forming assay

Dissociated hPSC-EBs were incubated with fluorochrome-conjugated mouse antibody anti-human CD144 (Miltenyi, clone REA199) for 30 min at 4 °C. Cells were washed and resuspended in PBS containing 5% FBS and 1 μg/mL of 7AAD (Sigma Aldrich). 7AAD^−^hPSC-EB-CD144^+^ cells were sorted with the BD FacsAria Sorter (BD Biosciences) and plated at 1 cell/well on fibronectin pre-coated 96-well plates under endothelial conditions. Endothelial medium was replaced twice per week. After 15 days in culture at 37 °C and 5% CO_2_, ECs colonies were counted, fixed with 4% paraformaldehyde (PFA, Electron Microscopy Sciences), and analyzed by immunofluorescence.

### Characterization of hPSC-ECs

All the in vitro hPSC-EC characterization tests were carried out with confluent hPSC-ECs at passage 2. Replicates correspond to hPSC-ECs obtained from independently performed hPSC-differentiation experiments. Endothelial colony-forming cells (ECFC) isolated from human umbilical cord blood and cultured following the protocol described somewhere else [38] were used as positive control.

#### LDL uptake assay

hPSC-ECs and ECFCs were incubated with endothelial medium supplied with 15 μg/mL of AF488-conjugated acetylated-LDL (Invitrogen) for 4 h at 37 °C and 5% CO_2_. After incubation, endothelial medium was removed. ECs were rinsed with PBS and fixed with 4% PFA in PBS for further immunofluorescence analyses.

#### Endothelial activation by TNF-α

hPSC-ECs and ECFCs were incubated with endothelial medium supplied with 10 ng/mL of TNF-α (Miltenyi) overnight at 37 °C and 5% CO_2_. Next day, TNF-α-treated and non-treated hPSC-ECs and ECFCs were analyzed for ICAM-1 expression by flow cytometry. Cells were considered as ICAM^High^ when their fluorescence intensity was higher than 5 × 10^5^.

#### Nitric Oxide (NO) detection

hPSC-ECs and ECFCs were incubated with endothelial medium supplied with 0.5% FBS and 1 μM DAF-FM-2A (Invitrogen) for 1 h at 37 °C and 5% CO_2_. Positive controls were stimulated with 100 ng/mL LPS during the night preceding the experience. After incubation with the probe, cells were washed, and fluorescence was analyzed by flow cytometry.

#### Matrigel-based tube formation assay

hPSC-ECs and ECFCs were detached enzymatically from the culture plates with Accutase and seeded on a layer of 9 mg/mL Matrigel (Corning) at 20,000 cells/cm^2^ with endothelial medium for 24 h at 37 °C and 5% CO_2_. ECs were imaged every two hours with an inverted phase contrast microscope (Leica), and images were analyzed with the Image J tool Angiogenesis analyzer (Carpentier G. Contribution: angiogenesis analyzer. ImageJ News. 2012;5). Analyses herein shown correspond to quantification of 8-h images when the maximum number of networks was obtained. At 24 h, cells were fixed and analyzed by immunofluorescence.

### Transduction of hPSC-ECs

hPSC-ECs derived from SA01 cell line were transduced with a lentiviral vector carrying the red fluorescence protein mCherry under the control of a CMV promoter (LV-CMV-mCherry-Puro, SignaGen Laboratories). Twenty-four hours after seeding hPSC-EB-CD144^+^ under endothelial conditions, cells were incubated with the lentiviral vector at multiplicity of infection (MOI) 10 for 12 h at 37 °C and 5% CO_2_ in the presence of 8 μg/mL polybrene (Sigma-Aldrich). After incubation, medium was replaced, and hPSC-ECs from SA01 cell line were recovered once they reached confluency. The transduction efficiency and the endothelial phenotype were evaluated by flow cytometry analysis. Only hPSC-ECs from SA01 cell line having more than 80% mCherry^+^ cells were used for in vivo experiments.

### Colony-forming cell (CFC) assay

Six-day-old hPSC-BCs were recovered and seeded for CFC assay at a density of 5 × 10^4^ cells/mL in MethoCult GF (H4435 StemCell Technologies) and Iscove′s modified Dulbecco’s medium (Gibco). Replicates correspond to hPSC-BCs obtained from independently performed hPSC-differentiation experiments. At day 14, hematopoietic CFCs (BFU-E, CFU-GM and CFU-GEMM) were counted and classified according to standard morphological criteria. BFU-E and CFU-GEMM were hand-picked and pooled in order to extract RNA for further hemoglobin quantification.

### Immunofluorescence microscopy

As previously described in earlier sections, samples were fixed with 4% PFA in PBS for 10 min and washed with PBS. Samples were further permeabilized with PBS 0.1% Triton, blocked with PBS 5% bovine serum albumin (BSA, Eurobio) and incubated with primary antibodies recognizing human CD31, CD144 and von Willebrand Factor (vWF) diluted according to the manufacturer’s conditions or previous experiences in PBS 1% BSA for 1 h at 37 °C (Additional file 10: Table S1). Excess of primary antibody was rinsed with PBS, and samples were incubated with fluorochrome-conjugated secondary antibodies and DAPI (Life technologies) for 1 h at 37 °C. After three washes with PBS, samples were mounted with Glycergel mounting medium (Dako). To verify the specificity of the conjugated secondary antibodies, control samples were treated following the aforementioned conditions in the absence of primary antibodies. Immunofluorescence images were acquired with the confocal microscope Leica TCS SP5 (Leica) and analyzed with software ImageJ.

Some adaptations of this protocol were necessary for treating hPSC-EB-CD144^+^ samples and the dorsal skinfold chambers. In both cases, incubation with 4% PFA was extended to 4 h. For the dorsal skinfold chambers, the epidermis still present at one side of the chamber was manually removed from the dermis. Permeabilization and blocking time were extended to 2 h at room temperature, and incubation of primary and secondary antibodies was performed overnight.

### Sorting and cell analysis by flow cytometry

#### Cell analysis

hPSC single-cell suspensions were incubated with fluorochrome-conjugated antibodies against the human pluripotency markers SSEA-3, SSEA-4 and TRA-1-81 (BD Sciences). Single-cell suspensions of hPSC-EBs, hPSC-ECs, hPSC-BCs and ECFCs were incubated with fluorochrome-conjugated mouse antibodies recognizing the human hematoendothelial markers CD34 (BD Sciences), CD143 (Miltenyi), and CD309 (Miltenyi), the endothelial markers CD144 and CD31 (Miltenyi), and the hematopoietic markers CD43, CD45 and CD41a (BD Sciences) (Additional file 10: Table S1). For the intracytoplasmic detection of eNOS, hPSC-ECs and ECFCs were treated with the FIX & PERM Cell Permeabilization Kit (ThermoFisher) according to the manufacturer’s instructions and incubated with mouse PE-conjugated antibody against human eNOS. For TNF-α activation tests, hPSC-ECs and ECFCs were incubated with anti-ICAM-FITC (Miltenyi). Blood samples from the NSG mice from the hematopoietic reconstitution experiences were treated with the ACK lysis buffer (Gibco) according to the manufacturer instructions to lyse red blood cells. Lysed blood samples and bone marrow mouse samples were further incubated with specific anti-human CD45 (BD Sciences) (Additional file 10: Table S1). Cells were incubated with either the aforementioned antibodies or conjugated isotypes for 30 min on ice, washed and resuspended in PBS containing 5% FBS and 1 μg/mL of 7AAD to exclude dead cells. Data acquisition was performed with the BD Accuri C6 cytometer, and analyses were carried out with the BD Accuri C6 Plus Software (BD Biosciences).

#### Cell sorting

Single-cell suspensions were incubated with anti-SSEA3-AlexaFluor647 for isolation of SA01 hPSCs, anti-CD144-PE for isolation of 84 h-hPSC-EB-CD144^+^, and anti-CD144-PE and anti-CD45-FITC for isolation of hPCS-BC-CD144^+^CD45^+^ following the same procedure as described in the “Cell analysis” section. Selected populations were isolated with a BD FacsAria Sorter (BD Biosciences).

### RT and qPCR

Total RNAs from hPSCs, 48 h-hPSC-EBs, 84 h-hPSC-EBs, hPSC-EB-CD144^+^, hPSC-EB-CD144^−^, hPSC-ECs, hPSC-BCs and red CFC (BFU-E + CFU-GEMM) were extracted with the RNeasy Micro Kit (Qiagen) and quantified with the NanoDrop^™^ 1000 Spectrophotometer (Thermo Fisher Scientific). Reverse transcription (RT) was further performed by using the SuperScript III kit (Invitrogen) with random hexamers. Three replicates per sample, each corresponding to 9 ng of cDNA, were analyzed for differential gene expression by using the Mx3000P qPCR thermocycler system (Agilent) with Brilliant III Ultra-Fast SYBR Green (Agilent) for most of the genes and with TaqMan Universal Master Mix II, no UNG (Applied Biosystems) for arterial/vein endothelial genes as shown in Additional file 11: Table S2 and Additional file 12: Table S3. mRNA extractions and RTqPCR conditions were set according to the manufacturer’s instructions. Target gene expression was normalized with the endogenous RNA control human 18S and ERCC3 according to the 2^−∆∆Ct^ method. Fold-change expressions were calculated with respect to the initial hPSCs of the same differentiation experiment for *NANOG, POU5F1, TBXT* and *KDR. ETV2* and *RUNX1* fold-change expressions in CD144^+^-hPSC-EBs and CD144^−^-hPSC-EBs were calculated with respect to non-sorted 84 h-hPSC-EBs. For arterial/venous phenotype, expressions were relative to ECFCs gene expressions. For the hematopoietic genes, mRNA expressions were compared to a total human EL samples.

For the mouse BM samples from the hematopoietic reconstitution experiments, qPCR was also used to quantify the presence of human CD45 DNA. DNA was extracted according to the instructions of the GeneJET Genomic DNA Purification kit (ThermoFisher Scientific) and quantified with the NanoDrop^™^ 1000 Spectrophotometer. Three replicates per sample, each corresponding to 36 ng of DNA, were analyzed for the presence of human CD45 DNA and normalized based on the mouse actin gene (Additional file 13: Table S4).

### In vivo experiments

#### Ischemic tissue injury model on a mouse dorsal skinfold chamber

Eight-week-old male nude mice were obtained from the Animal Core Facility of Gustave Roussy Institute. The surgical procedure followed a previously described protocol [39]. Briefly, after 100 µL subcutaneous injection of Lidocaine (21.33 mg/mL), the dorsal skinfold of an anesthetized mouse (mix 1.5% isoflurane with air at 1.5 L/min) was stretched keeping the dorsal median line at the top. The two faces of the dorsal chamber were positioned on each side of the skinfold and secured by sutures between two adjacent orifices. To prepare the side of the chamber where cells would be injected, the entire epidermis and upper dermis were removed as close as possible to the edges of the chamber. Fifteen nude mice were grouped in 3 experimental groups as follows. 6 nude mice were injected intradermally on the dorsal chamber with 3 × 10^5^ mcherry hPSC-ECs from SA01 cell line resuspended in 20 μL of PBS. On 9 nude mice, an ischemia was induced by thermal cauterization of a macrovessel 15 min before cell injection; 6 of them were further injected with 3 × 10^5^ mcherry hPSC-ECs resuspended in 20 μL of PBS and 3 of them with 20 μL of PBS. In all the cases, hPSC-ECs or PBS controls were injected next to the cauterized blood vessel. The injected side of the chamber was covered with a glass coverslip sealed under mechanical pressure by the rings. A subcutaneous injection of Tolfédine (4 mg/kg) was administrated at the end of the surgical procedure before mouse awakens. Mice were maintained for a maximum of 30 days after dorsal chamber implantation. Once per week, anesthetized mice (mix 1.5% isoflurane with air at 1.5 L/min) were injected with 70 kDa-FITC dextran intravenously in the retro-orbital sinus (100 µL), and images of the dorsal chamber were acquired with a confocal microscope (SP8, Leica) in brightfield and fluorescence mode. At day 30, after the last acquisitions, mice were euthanatized by progressive doses of CO_2_ according to ethical guidelines, and the dorsal chambers were recovered to perform further immunofluorescence analyses as described above.

#### Quantification of the blood vessels at the dorsal skinfold chamber

Treatments of macroscopy images of the dorsal chambers were performed with the software ImageJ. PBS controls were used to set up the parameters for background subtraction and blood vessel quantification. The plugin Tubeness [40] followed by Analyze skeletons 2D/3D [41] were used on all the images following the parameters selected for the control. The density of blood vessels was reported as the total branch length measured in the dorsal chamber for day 5 and day 19 post-injection. For every mouse, we calculated the change in percentage of the total branch length of day 19 compared to day 5.

#### Hematopoietic reconstitution in irradiated immunosuppressed mouse

For the hematopoietic reconstitution, NOD.Cg-PrkdcscidIl2rgtm1Wjl/SzJ mice (NSG) obtained from the Animal Core Facility of Gustave Roussy Institute were bred and maintained under specific pathogen-free conditions with acidified water (pH 5.3). Six- to eight-week-old mice were irradiated at 2.5 Gy with an IBL637 gamma irradiator containing a Cs-137 source. After 24 h, mice were subjected to isoflurane anesthesia and submitted to intra-BM transplantation (IBMT) in the right femur with 4 × 10^5^ hPSC-EB-CD144^+^ in 15 µL PBS. Peripheral blood was obtained from the retro-orbital sinus of anesthetized mice and analyzed at 6 weeks post-transplant for the presence of human hematopoietic cells by flow cytometry analysis as described previously [42]. Mice were euthanatized at 12 weeks post-transplant. BM cells were collected and analyzed for the presence of human hematopoietic cells (hCD45) by flow cytometry. Genomic DNA was extracted from the BM of the right (injected) and left (non-injected) femurs and analyzed by quantitative PCR (Q-PCR) as described in materials.

### Human EL isolation and cell preparation

Human embryos (range 7–8 weeks of gestation) were obtained following voluntary abortions. Developmental age was estimated based on several anatomic criteria according to the Carnegie classification for embryonic stages 74 and by ultrasonic measurements. ELs were excised sterilely using microsurgery instruments and a dissecting microscope in phosphate-buffered saline (PBS) supplemented with 10% fetal bovine serum (FBS, Hyclone Laboratories). ELs were then disrupted mechanically through successively 18-, 23- and 26-gauge needles. Cells clumps were removed on a 70-μm nylon filter (Invitrogen), washed three times with Dulbecco’s modified Eagle medium (DMEM; Gibco)/FBS 10% and quantified. Mononuclear cells were isolated using Pancoll centrifugation gradient (Dominique Dutscher). Mononuclear cells from human EL were incubated with anti-CD45-FITC, anti-CD144-PE and anti-CD34-APC antibodies for 30 min at 4 °C. Selected CD144^+^CD45^−^CD34^+^ EL-EC, CD144^+^CD45^low^CD34^+^ EL-pre-HSC, CD144^−^CD45^low^CD34^+^ EL-HSC/HP and CD144^−^CD45^high^CD34^−^ mature EL-HC cell populations were sorted by flow cytometry (Table 2) as described previously [16].

**Table 2:** Human embryonic liver (EL) sorted cell populations

Sorted cell populations	Name
EL CD144^+^CD45^−^CD34^+^ cells	EL-ECs
EL CD144^+^CD45^low^CD34^+^ cells	EL-pre-HSCs
EL CD144^−^CD45^low^CD34^+^ cells	EL-HSCs/HPs
EL CD144^−^CD45^high^CD34^−^ cells	EL-mature HCs

### Transcriptomic analyses

For the transcriptomic study, hPSC-ECs, hPSC-EB-CD144^+^, hPSC-BCs and hPSC-BC-CD144^+^CD45^+^ (Table 1) were derived from undifferentiated hESC (H1 cell line). CD144^+^CD45^−^CD34^+^ EL-EC, CD144^+^CD45^low^CD34^+^ EL-pre-HSC, CD144^−^CD45^low^CD34^+^ EL-HSC/HP and CD144^−^CD45^high^CD34^−^ EL-mature HC cell populations were freshly isolated from human ELs (Table 2). Total RNAs were purified using Direct-Zol ^™^RNA kit (ZymoResearch) according to manufacturer’s protocol. After validation of the RNA quantity and quality with the Bioanalyzer 2100 (using Agilent RNA6000 nano chip kit), 10 ng of total RNA was reverse-transcribed following the Ovation PicoSL V2 system (Tecan). Briefly, the resulting double-strand cDNA was used for amplification based on SPIA technology. After purification according to Tecan protocol, 3.6 ug of Sens Target DNA were fragmented and biotin labelled using Encore Biotin Module kit (Tecan). After control of fragmentation using Bioanalyzer 2100, cDNA was then hybridized to GeneChip® HumanGene 2.0 ST (Affymetrix) at 45 °C for 17 h. Chips were washed on the fluidic station FS450 following specific protocols (Affymetrix) and scanned using the GCS3000 7G. The scanned images were then analyzed with Expression Console software (Affymetrix) to obtain raw data (cel files) and metrics for quality controls. RMA normalization was performed using TAC4.0 software (Thermofisher) with default parameter.

Data were first explored with unsupervised learning methods using the Perseus software platform (http://www.perseus-framework.org) v1.6.14.0 to perform principal component analyses and hierarchical clustering (with Pearson correlation).

Supervised analyses were performed using TAC4.0 software to identify transcripts specifically up- or down-regulated in each pairwise cell population comparison (Additional file 8: Fig. S8). For each cell population, a Tukey's biweight robust mean is calculated from log2 expression values. Mean ratio is then calculated from unlog mean values. If ratio > 1 , then FoldChange = ratio, and if ratio < 1 then FoldChange = − 1/ratio. Statistical pValue is calculated after ANOVA test using ebayes model.

Enrichments were also explored using GSEA (Gene Set Enrichment Analysis) v4.1.0 software (https://www.gsea-msigdb.org/gsea/). Gene Set Enrichment Analysis determined whether gene sets from the Molecular Signatures Database (MSigDB) Gene Ontology Biological Process collection (c5.go.bp.v7.4) were randomly distributed throughout our ranked transcript lists or if they were located at the top or bottom of them. The selected parameters were: Signal2Noise metric, gene set permutation (*n* = 1000).

Datasets generated and analyzed during the current transcriptomic study are available in GEO (accession number GSE194372) and can be queried via the following link [https://www.ncbi.nlm.nih.gov/geo/query/acc.cgi?acc=GSE194372].

### Statistics analyses

Sample sizes were determined by the nature of the experiment and variability of the output, not by a statistical method. Statistical analyses were performed in PRISM software. Data obtained from multiple experiments were reported as mean ± standard deviation (SD). *n* = 5 in most all the experiments unless stated differently. Where appropriate, either a paired parametric *t*-test or a one-way ANOVA with a Tukey post hoc test. Differences were considered significant when *P < 0.05, **P < 0.005, or ***P < 0.0005.

## Results

### hPSCs differentiation into CD144^+^ hematoendothelial progenitors

**Fig. 1:**
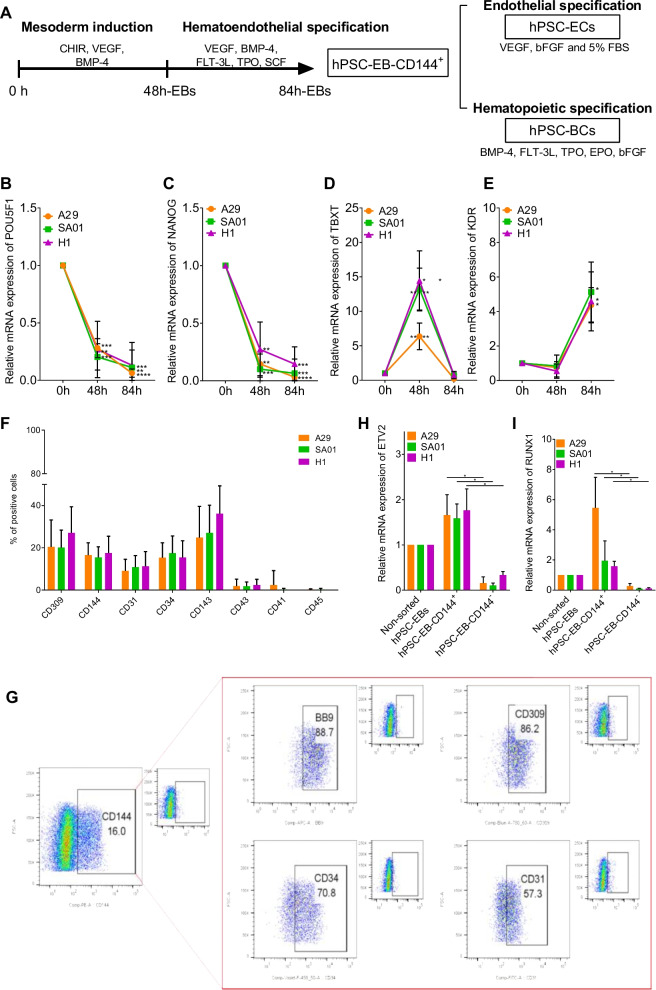
hPSC differentiation into a hematoendothelial population. **A** One hiPSC cell line (A29) and two hESC cell lines (SA01 and H1) were differentiated following embryoid body (EB) formation for 84 h supplied with growth factors inducing mesoderm and hematoendothelial specification. CD144^+^-sorted cells were differentiated into endothelial and hematopoietic lineage in the presence of specific growth factors. **B**–**E** EBs were analyzed at 48 h and 84 h by RTqPCR and compared to the hPSCs preceding EB formation (0 h) for the stem cell genes *POU5F1* (**B**) and *NANOG* (**C**), the mesoderm gene *TBXT* (**D**) and the hematoendothelial gene *KDR* (**E**). **F** At 84 h, EBs were dissociated enzymatically and analyzed by flow cytometry for the expression of hematoendothelial (CD309, CD34 and CD143), endothelial (CD144 and CD31) and hematopoietic markers (CD43, CD41 and CD45), *n* = 30 for every hPSC cell line and marker. **G** Representative flow cytometry analysis of CD143, CD309, CD34 and CD31 within the CD144+ population from 84 h-EBs. **H**–**I** 84 h-EBs were sorted based on the expression of CD144 and analyzed for the expression of the hematoendothelial transcription factors *ETV2* (h) and *RUNX1* (I) by RTqPCR, *n* = 5 independent experiments for each cell line. *P value < 0.05; **P value < 0.005, ***P value < 0.0005. Data are represented as mean ± SEM

hPSC-EBs were generated from one hiPSC line, A29, and two hESC lines, SA01 and H1, following an adapted version of the Lu et al. protocol [36]. Briefly, mesoderm was induced during the first 48 h of hPSC-EB formation followed by hematoendothelial specification step from the mesoderm during the next 36 h in a serum-free medium (Fig. 1A). A progressively loss of pluripotency during hPSC-EB formation was confirmed by a decrease in the mRNA levels of two pluripotency markers, *POU5F1 (OCT3/4*) and *NANOG*, for all hPSCs (Fig. 1B, C). This loss of pluripotency coincided in time with a peak in the expression of the mesodermal marker *TBXT* at 48 h (Fig. 1D) followed by a significant increase in the expression of *KDR* (CD309) at 84 h of hPSC-EB formation (Fig. 1E). No significant differences in this kinetics were observed among the hPSC lines.

Since *KDR* (CD309) has been described as an important marker of endothelial and hematopoietic precursors [43], we hypothesized that the increased in *KDR* expression may mark the apparition of hematoendothelial progenitors within 84 h-hPSC-EBs. Thus, we analyzed the expression of other endothelial and hematopoietic markers in 84 h-hPSC-EB by flow cytometry (Fig. 1F, Additional file 14: Table S5, Additional file 1: Fig. S1A–B). Between 15 to 25% of cells within 84 h-hPSC-EBs expressed hematoendothelial markers as CD309, CD34 and CD143, and the endothelial marker CD144, independently on the hPSC line. Fewer cells (≈ 10%) expressed the endothelial marker CD31, and almost none expressed the hematopoietic markers CD43, CD45 and CD41. Interestingly, the CD309^+^ population did not contain exclusively hematoendothelial progenitors since only 60% of these CD309^+^ cells coexpressed CD34, CD143 and CD144 (Additional file 1: Fig. S1B). Instead, the CD144^+^ population seemed a more homogeneous population composed by more than 90% of CD34^+^CD143^+^CD309^+^ cells (Fig. 1G). CD144 is a classical endothelial marker expressed by both hemogenic and non-hemogenic endothelium. Thus, the CD144^+^ population was isolated from 84 h-hPSC-EBs as a possible source of both ECs and HCs cells. As expected, hPSC-EB-CD144^+^ expressed significantly higher levels of two master transcription factors required for endothelial and hematopoietic specification, *ETV2* and *RUNX1*, compared to the non-sorted 84 h-hPSC-EBs and hPSC-EB-CD144^−^ (Fig. 1H–I). No significant differences among the hPSC lines were observed. These results suggest that hPSC-EB-CD144^+^ may contain not only ECs but also hemogenic ECs.

### hPSC-EB-CD144^+^ generates functional and mature hPSC-ECs

**Fig. 2:**
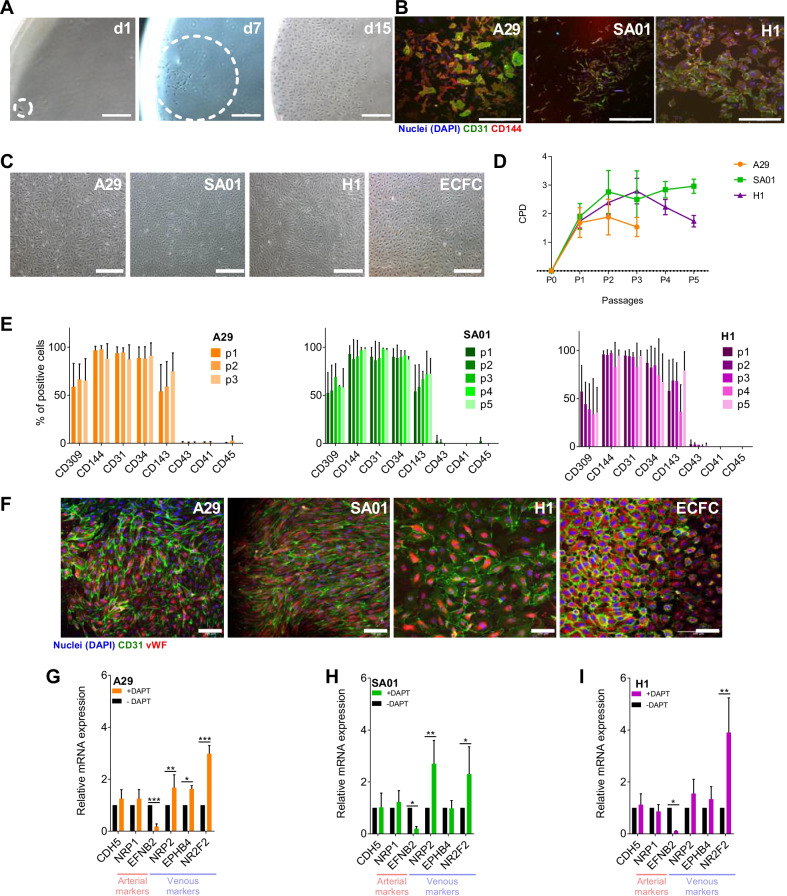
Endothelial differentiation of hPSC-EB-CD144^+^. **A** Representative phase-contrast images of endothelial colonies formed from single CD144^+^ cells at day 0 (left), day 7 (middle) and at day 15 (right). Scale bar 100 µm. **B** Confocal microscope images of endothelial colonies from the colony-forming assay. hPSC-ECs were labeled against CD31 (green), CD144 (red) and with DAPI for the nuclei (blue). Scale bar 500 µm. **C** Representative phase-contrast images of endothelial colony-forming cells (ECFC) and hPSC-derived endothelial cells (hPSC-ECs) from CD144+-EBs. Scale bar 500 µm. **D** Cumulative population doubling (CPD) of hPSC-ECs along passages. **E** Endothelial phenotype was analyzed along the passages by flow cytometry for hPSC-EC from A29 (left), SA01 (center) and H1 (right) cell lines. hPSC-ECs derived from the three cell lines kept a stable phenotype characterized by the expression of CD309, CD144, CD31, CD34 and CD143 (see also Additional file 2: Fig. S2). The expression of hematopoietic markers was negligible at every passage. **F** Confocal microscope images of hPSC-ECs and ECFC labeled with antibodies against von Willebrand Factor (vWF, red) and CD31 (green). Nuclei stained with DAPI (blue). Scale bar 500 µm. **G**–**I** RTqPCR analysis of arterial (*NRP1* and *EFNB2*), venous genes (*NRP2, EPHB4* and *COUPTFII*) and the pan-endothelial gene *CDH5* (CD144) in hPSC-ECs after treatment with an antagonist of Notch (DAPT) during the EC differentiation, *n* = 5 independent experiments for every cell line. *P value < 0.05; **P value < 0.005 and ***P value < 0.0005. Data are represented as mean ± SEM

We first sought to determine whether hPSC-EB-CD144^+^ include proliferative endothelial progenitors*.* hPSC-EB-CD144^+^ were seeded at one cell per well in a 96-well plate. Small colonies were observed at day 7 and kept growing until day 15 confirming the existence of proliferative ECs within hPSC-EB-CD144^+^ (Fig. 2A, B). Similar results were obtained when fresh and frozen hPSC-EB-CD144^+^ were cultured at a normal cell density. hPSC-EB-CD144^+^ differentiated into typical endothelial cobblestone-like cells that proliferate until reaching confluence (Fig. 2C). Remarkably, their cumulative population doublings (CPD) decreased along passages suggesting hPSC-ECs do not proliferate indefinitely (Fig. 2D). It is worth noting that CPD was dependent on the hPSC line, hPSC-ECs coming from A29 reached a plateau earlier than hPSC-ECs derived from SA01 and H1. However, we did not observe significant differences in their phenotype among hPSC lines and passages (Fig. 2E, F, Additional file 15: Table S6, Additional file 2: Fig. S2A). In all cases, hPSC-ECs were composed of more than 90% cells expressing CD144, CD31, CD34 and vWF (Fig. 2E, F, Additional file 2: Fig. S2B). Most of these cells also expressed CD309 and CD143. We did not observe the expression of the hematopoietic markers CD43, CD45 and CD41 (Fig. 2E, Additional file 2: Fig. S2B).

Next, hPSC-EB-CD144^+^ were cultured in the presence or absence of an inhibitor of Notch pathway (DAPT). Notch signaling pathway plays a pivotal role in arterial but not in venous differentiation [44]. hPSC-EB-CD144^+^ proliferated and formed a confluent homogeneous layer of hPSC-ECs in both conditions, independently on the hPSC line of origin (Fig. 2C). Their endothelial identity was confirmed by flow cytometry analysis, showing that more than 95% of cells expressed CD309, CD144, CD31, CD34 and CD143 independently on the presence or absence of DAPT. No expression of the hematopoietic markers CD43 was detected (Additional file 3: Fig. S3A). Similarly, RTqPCR analysis revealed no differences in the expression of the panendothelial marker *CDH5*, suggesting that the inhibition of the Notch pathway does not affect the differentiation of hPSC-EB-CD144^+^ into hPSC-ECs. No differences in the expression of the arterial marker *NRP-1* were observed between DAPT-treated and DAPT-untreated ECs (Fig. 2G–I). However, DAPT-treated ECs downregulated the arterial marker *EFNB2*. Some heterogeneity between hPSC lines was observed regarding the expression of venous markers. While hPSC-EC from A29 cell line cultured in the presence of DAPT showed a significantly higher expression of *NRP2, EPHB4* and *NR2F2* compared to the control, hPSC-EC from SA01 and H1 cell lines only showed upregulation of *NRP2* and *NR2F2*, and *NR2F2*, respectively (Fig. 2G–I). These results suggest that the hPSC-EB-CD144^+^ arterial/venous specification may be guided in vitro under the appropriate culture conditions although their response to environmental factors might be influenced by the hPSC line from which they are derived.

**Fig. 3:**
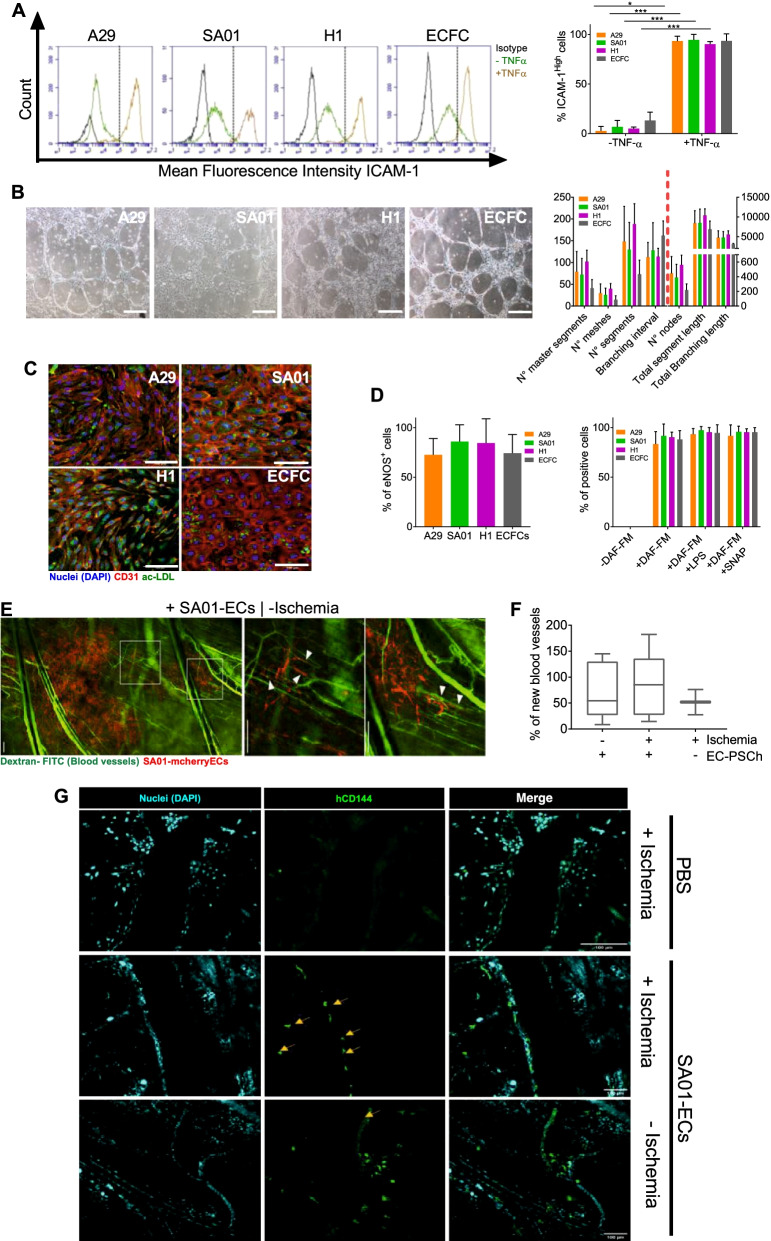
hPSC-EB-CD144^+^-derived endothelial cells are functional in vitro and in vivo. **A** Endothelial activation by TNF-α assay. Representative histograms of flow cytometry analysis for ICAM-1 expression in hPSC-ECs and ECFCs (left panel) and quantification of ICAM-1^High^ cells (right panel) in the absence and presence of TNF-α. **B** Matrigel-based tube formation assay 8 h after seeding. Phase contrast images with scale bar 1000 µm (left panel) and cell network characterization based on the number of segments, meshes, branches and nodes (right panel). **C** Confocal microscopy images of hPSC-ECs and ECFCs after 4 h of incubation with acLDL-AlexaFluor-488. CD31 in red and nuclei in blue (DAPI). Scale bar 100 µm. **D** hPSC-ECs nitric oxide (NO) production was analyzed based on the expression of the endothelial nitric oxide synthase (eNOS) measured by flow cytometry (left histogram) and indirectly, by using the probe DAF-FM diacetate to detect NO in hPSC-ECs and ECFCs. DAF-FM fluorescence was measured by flow cytometry in cells that were not incubated with the probe (-DAF-FM) as a negative control, in cells incubated with the probe alone (+ DAF-FM) and in the presence of a S-nitrosothiol molecule (+ DAF-FM + SNAP) or in cells previously stimulated with lipopolysaccharides (+ DAF-FM + LPS), the two latter as positive controls, *n* = 5 independent experiments for every cell line. **E** Representative confocal image at day 19 post-injection of a mouse dorsal skinfold chamber without ischemia, injected with mcherry-hPSC-ECs. Vessel perfusion was observed thanks to the injection of dextran 70 kDa-FITC (green). Scale bar: 200 µm. **F** Percentage of new blood vessels formed at day 19 post-injection. *n* = 6 independent experiments. **G** Confocal images of the dorsal chambers analyzed post-mortem by immunofluorescence. Yellow arrows indicate human CD144^+^ ECs (green) localized at the wall of blood vessels. Scale bar: 100 µm. *P value < 0.05; **P value < 0.005 and ***P value < 0.0005. Data are represented as mean ± SEM

We also investigated whether hPSC-EB-CD144^+^ were able to differentiate into fully mature ECs in vitro*.* To this, we compared the in vitro functionality of hPSC-ECs to human umbilical cord blood endothelial progenitors, ECFCs. More than 90% of hPSC-ECs and ECFC expressed high levels of the adhesion molecule ICAM-1 in response to TNF-α. In contrast, less than 1% of untreated cells expressed similar levels of ICAM-1, suggesting that hPSC-ECs may be activated by proinflammatory cytokines (Fig. 3A, Additional file 16: Table S7). All hPSC-ECs formed a tubular network comparable to the one formed by ECFCs after seeding on Matrigel (Fig. 3B and Additional file 3: Fig. S3B, Additional file 16: Table S7). No significant differences in the number of master segments and meshes, neither in the total segment length nor in the branching length, were observed among hPSC lines and ECFCs (Fig. 3B and Additional file 16: Table S7). However, ECFCs formed networks with a slightly reduced number of segments, nodes and branching intervals compared to hPSC-ECs (Fig. 3B).

In addition, ac-LDL was observed in the cytoplasm of hPSC-ECs derived from A29, SA01 and H1 hPSCs in a similar manner than ECFCs (Fig. 3C) after 4 h of incubation with a fluorochrome-conjugated ac-LDL, suggesting that hPSC-ECs are able to endocyte LDL. Similarly, the production of nitric oxide (NO) by the endothelial nitric oxide synthase (eNOS) is known to play an important role in endothelial homeostasis, inflammation, and vasodilation. We detected eNOS in 72 to 86% of hPSC-ECs and 84.5% of ECFCs (Fig. 3D, Additional file 16: Table S7 and Additional file 4: Fig. S4A). We further analyzed NO production by using the probe DAF-FM diacetate. This permeable molecule is de-acetylated inside the cell and reacts with NO to form fluorescent DAF-FM that can be detectable by flow cytometry. In the absence of the probe, no fluorescence was detected for any of the hPSC-ECs nor ECFCs. However, after incubation with the probe, we detected the fluorescent DAF-FM in 83 to 92% of hPSC-ECs and 88% of ECFCs (Fig. 3D, Additional file 16: Table S7 and Additional file 4: Fig. S4B). Slightly increase of DAF-FM fluorescence was detected when ECs were incubated with LPS (known to induce the NO production) and with a S-nitrosothiol molecule (SNAP) as a positive control.

Finally, we tested the in vivo angiogenic potential of hPSC-ECs in a mouse dorsal chamber model where an ischemic injury has been induced. Since not apparent differences in the functionality of the three hPSC-ECs were observed in vitro, we performed the in vivo experiments with mcherry-ECs derived from SA01 cell line. mcherry-ECs were detected for 4 weeks in the mice. Real time in vivo imaging showed that mcherry-ECs migrated and localized around the proximities of mouse blood vessels at day 5 independently on the induction or not of an ischemia. Intravital microscopy analysis demonstrated hPSC-ECs appear to line up around blood vessels and capillaries (Fig. 3E). Quantification of the blood vessels shows no significant differences between the analyzed conditions, although the number of new blood vessels was slightly higher in mice injected with mcherry-ECs (Fig. 3F, Additional file 5: Fig. S5A–B). The immunofluorescent analysis postmortem confirmed the survival of human CD144^+^ hPSC-ECs in both ischemic and non-ischemic mice and their localization at the wall of the blood vessels. No teratomas or any other complications were observed at the dorsal chambers. Thus, SA01-ECs survive and participate to the formation of new vessels in vivo (Fig. 3G).

### hPSC-EB-CD144^+^ contain hemogenic hPSC-ECs

**Fig. 4:**
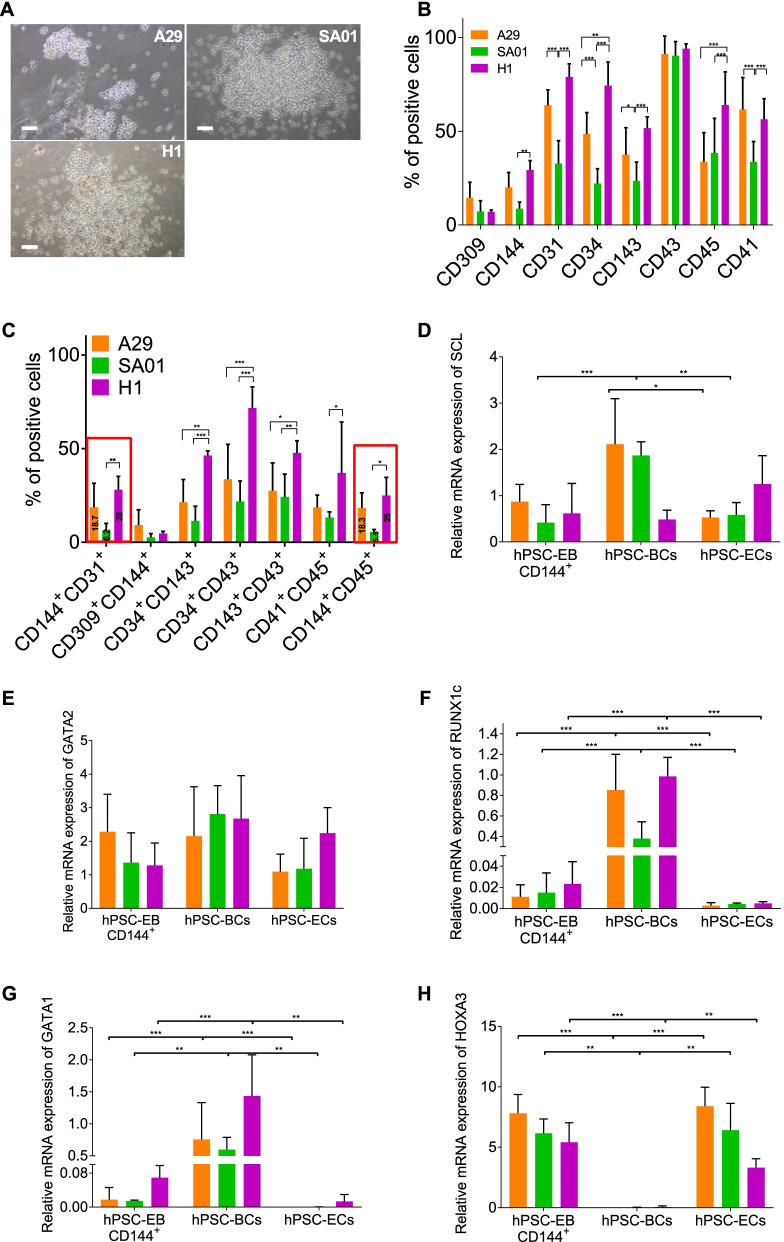
Differentiation of hPSC-EB-CD144^+^ into hPSC-BCs. **A** Phase-contrast microscopy images of three hPSC-derived blast colonies (hPSC-BCs) A29, SA01 and H1 cell lines. Scale bar 100 µm. **B**, **C** Flow cytometry analysis of day 6 hPSC-BCs for hematoendothelial (CD309, CD143 and CD34), endothelial (CD144 and CD31) and hematopoietic markers (CD43, CD45 and CD41) as simple (**B**) and double labelling (**C**), *n* = 15 for every hPSC line and marker. **D**–**H** Expression of hematopoietic and endothelial transcription factors in CD144^+^-EBs, hPSC-ECs and hPSC-BCs: *SCL* (**D**), *GATA*2 (**E**), *RUNX1c* (**F**), *GATA1* (**G**) and *HOXA3* (**H**). Data are represented as mean ± SEM, *n* = 5 independent experiment for each cell line. *P value < 0.05; **P value < 0.005 and ***P value < 0.0005

To confirm the existence of hemogenic ECs within the hPSC-EB-CD144^+^ population, fresh and frozen hPSC-EB-CD144^+^ and hPSC-EB-CD144^−^ cells were seeded on a serum-free methylcellulose enriched with hematopoietic cytokines. After 4 to 6 days, hPSC-BCs were observed for the three hPSC lines only in the CD144^+^ fraction (Fig. 4A). Flow cytometry analysis showed that more than 90% of hPSC-BCs were positive for the hematopoietic marker CD43 (Fig. 4B, Additional file 17: Table S8, Additional file 6: Fig. S6A). Analyses of other hematopoietic and endothelial markers revealed heterogeneity within hPSC-BCs. Between 33 and 64% of hPSC-BCs expressed CD41 and 34 and 62% of hPSC-BCs expressed CD45 (Fig. 4B, Additional file 17: Table S8, Additional file 6: Fig. S6A). In addition, a subpopulation of hPSC-BCs coexpressed the hematoendothelial markers CD34 and CD143 (11–46%). Interestingly, only a small fraction of hPSC-BCs retained the expression of CD144 and around 5 to 25% of hPSC-BCs co-expressed CD144 and CD45 markers (Fig. 4C, Additional file 17: Table S8, Additional file 6: Fig. S6B), suggesting that hPSC-EB-CD144^+^ may undergo endothelial-to-hematopoietic transition (EHT) to give rise to HCs. Even though hPSC-BCs were observed from all the three hPSC lines, significant differences in the expression of hematopoietic and endothelial markers were observed among the hPSC-BCs. Notably, hPSC-BCs from H1 expressed significantly more CD34, CD143, CD45 and CD41, suggesting a difference in hematopoietic potential among the hPSC cell lines (Fig. 4B, C, Additional file 17: Table S8).

We further analyzed the expression of key hematopoietic transcription factors in hPSC-BCs compared to hPSC-EB-CD144^+^ and hPSC-ECs RTqPCR. Consistently with their common origin and the role of *SCL* and *GATA2* in both endothelial and hematopoietic differentiation, *SCL* and *GATA2* mRNAs were detected in hPSC-EB-CD144^+^, hPSC-ECs and hPSC-BCs independently on the hPSC-line (Fig. 4D, E). However, the expression of *SCL* was significantly higher in hPSC-BCs for A29 and SA01 (Fig. 4D). hPSC-BCs expressed significantly higher levels of the *RUNX1* isoform c and *GATA1*; transcription factors implicated in HSC emergence and erythropoiesis, respectively (Fig. 4F, G). In addition, hPSC-BCs expressed reduced levels of *HOXA3* compared to hPSC-EB-CD144^+^ and hPSC-ECs, a transcription factor key for arterial and hemogenic endothelium determination (Fig. 4H).

**Fig. 5:**
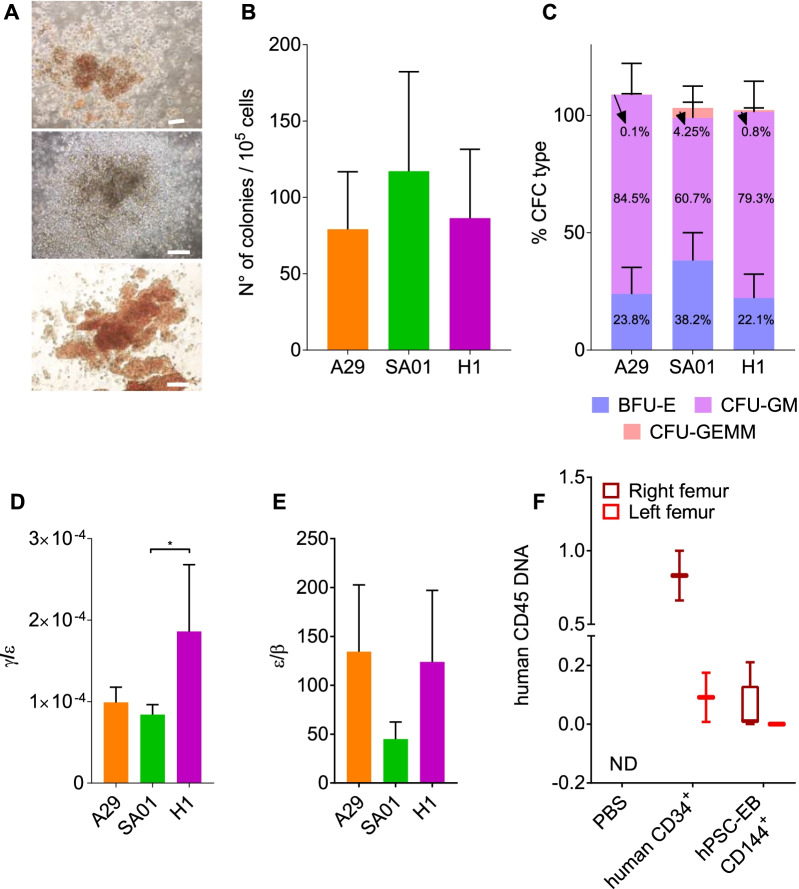
Functional characterization of hematopoiesis. **A** Phase-contrast microscopy images of myeloid, CFU-GEMM (top image) and CFU-GM (middle image) (image above right), and erythroid colonies, BFU-E (bottom image), derived from hPSC-BCs. Scale bar: 100 µm. **B** Number of hematopoietic colonies per 10^5^ hPSC-BCs seeded cells in methylcellulose (A29 *n* = 7, SA01 *n* = 6 and H1 *n* = 10). **C** Percentage of every type of myeloid and erythroid colony per hPSC line. **D** Ratio of gamma/epsilon and **E** epsilon/beta hemoglobin expression of the BFU-E and CFU-GEMM derived from hPSCs. **F** Presence of human DNA in the mouse BM was assessed by qPCR with primers amplifying specifically the human gene CD45. Data are represented as mean ± SEM. For the in vivo experiment: ND: non-detected, *n* = 2 for CD34^+^ and PBS and *n* = 5 for CD144^+^-EBs. For all the other histograms: *n* = 5 independent experiments for each cell line. *P value < 0.05; **P value < 0.005 and ***P value < 0.0005

Given the hematopoietic profile of hPSC-BCs, we next assessed hPSC-BC hematopoietic potential in vitro. hPSC-BC derived from hiPSCs and hESCs lines gave rise to myeloid and erythroid colonies CFU-GM, CFU-GEMM and BFU-E with an efficiency of 79 to 117 CFU per 10^5^ seeded hPSC-BCs (Fig. 5A, B). From these, most of the colonies accounted for CFU-GM (61–85%), around 22 to 38%, corresponded to BFU-E and only 0.1 to 4% were CFU-GEMM, confirming the heterogeneity previously described by flow cytometry (Fig. 5C). Although similar percentages were found for the three hPSC lines, it is worth noting that hPSC-BCs from SA01 generated slightly more CFU-GEMM (4.3 ± 2.4%) than A29 cell line (0.1 ± 0.4%) and H1 (0.8 ± 0.9%) (Fig. 5C). Since hemoglobin subunits are differentially expressed during the embryonic, fetal, and adult erythropoiesis, we analyzed by RTqPCR the expression of the embryonic (*ε*), fetal (*γ*) and adult (*β*) hemoglobin chains from hPSC-derived CFU-GEMM and BFU-E colonies, and we compared them to the embryonic human liver transcripts. CFU-GEMM and BFU-E derived from hPSCs expressed significantly higher levels of *ε* hemoglobin (*HBE1*) (10–10^3^-fold change) compared to ELs (Additional file 7: Fig. S7). In contrast, all the hPSC-derived hematopoietic colonies expressed significantly less *γ* (*HBG1*) (0.025–0.12-fold change) and *β* (HBB) hemoglobin (7 × 10^–5^–1 × 10^−4^-fold change) than human ELs (Additional file 7: Fig. S7). In other words, hPSC-derived CFU-GEMM and BFU-E expressed around 10^4^ times more *ε* than *γ* hemoglobin (Fig. 5D) and 10^2^ times more *ε* than *β* hemoglobin (Fig. 5E). These results suggest that our protocol yields principally embryonic erythrocytes and, to lesser extent, definitive erythrocytes.

To confirm the existence of bona fide HSCs/HPs in hPSC-EB-CD144^+^, we analyzed the in vivo hematopoietic reconstitution potential of hPSC-EB-CD144^+^ in a xenograft model of immunodeficient NSG mice. When hPSC-EB-CD144^+^ from H1 were intravenously injected, we did not observe any hematopoietic engraftment in the transplanted mice while human cord blood CD34^+^ cells engrafted efficiently (data not shown). To circumvent any caveat due to homing defects, we injected hPSC-EB-CD144^+^ cells directly into the right femur of sublethally irradiated NSG mice by intra-BM transplantation (IBMT). Mice were monitored for human reconstitution by peripheral blood analyses (at 6 weeks, see Table 3) and were euthanatized at 12 weeks post-transplant. BM was, then, analyzed for human CD45 expression by FACS analysis, and the presence of human DNA in the BM of the right and left femur of transplanted mice was also analyzed by qPCR. Six weeks after transplantation, we observed human CD45^+^ cells in the peripheral blood of mice injected with human cord blood CD34^+^ cells (1% and 9%) and with hPSC-EB-CD144^+^ from H1 (between 0.5 and 3.6%). We did not detect any human CD45^+^ in the control mice injected with PBS (Table 3). After 3 months of engraftment, human-specific CD45 was detected by flow cytometry only in the right femur in 5 out of 5 mice injected with hPSC-EB-CD144^+^ from H1 (between 0.1 and 0.6%) (Table 3). In the mice injected with human umbilical cord blood CD34^+^ cells as control, human-specific CD45 was detected in both right (9% and 10%) and left femurs (1% and 5%) (Table 3). These results were further confirmed by qPCR where CD45 human-specific DNA was detected in the right femur for hPSC-EB-CD144^+^ and in the right and left femur for hCD34^+^ cells from human cord blood. One left femur out of five was also positive for human DNA; however, it was negative for CD45 in the flow cytometry analysis. Mice injected with PBS did not express human-specific CD45 neither contained human DNA in the femurs (Fig. 5F). No developed lymph nodes or tumors were observed in any of the examined mice. These results suggest hPSC-EB-CD144^+^ have a hematopoietic reconstitution potential only by IBMT; however, this is considerably lower compared to human CD34^+^ isolated from umbilical cord blood.

**Table 3:** Hematopoietic reconstitution in NSG mice

Samples	Human CD34		hPSC-EB-CD144^+^					PBS	
	#1	#2	#1	#2	#3	#4	#5	#1	#2
%hCD45 in the PB	9%	1%	1.5%	0.6%	0.5%	0.9%	3.6%	0%	0%
%hCD45 in the BM									
RF	10%	9%	0.1%	0.3%	0.1%	0.1%	0.6%	0%	0%
LF	5%	1%	0%	0%	0%	0%	0%	0%	0%
Human DNA fold change									
RF	1	0.662	0.007	0.210	0.013	0.0004	0.051	ND	ND
LF	0.175	0.008	ND	ND	0.0002	ND	ND	ND	ND

Percentage of human CD45^+^ cells detected by flow cytometry in peripheral blood (PB) and in the bone marrow (BM) from right (RF) and left femur (LF) of NSG mice injected with either human CD34^+^ cord blood cells, hPSC-EB-CD144^+^ cells or PBS. Relative quantification of human DNA (based on the amplification of human CD45 gene) in BM of mice injected with human cells or PBS. ND: non-detected.

### Transcriptomic profile of CD144^+^-EBs, hPSC-ECs and hPSC-BCs

To investigate whether hPSC-EB-CD144^+^, hPSC-ECs and hPSC-BCs may correspond to their physiological equivalent, we performed a transcriptome analysis to compare hPSC-derived cell populations to undifferentiated hPSC, to differentiated-hPSC themselves and to human EL cell populations. In human, EL is considered as a major organ of hematopoiesis during development. From day 30 to the 30th week of human gestation, mature ECs (CD144^+^CD34^+^CD45^−^) coexist with diverse hematopoietic populations resulted from the migration, proliferation and maturation of HCs, namely pre-HSCs (CD144^+^CD34^+^CD45^Low^), HSCs and HPs (CD144^−^CD34^+^CD45^Low^) and mature HCs (CD144^−^CD45^High^) [14]. Thus, EL allow us to compare hPSC-derived cells to ECs and HCs, including intermediate pre-HSCs no longer found in adults but also HSCs/HPs and mature HCs, all with an identical genetic background.

**Fig. 6:**
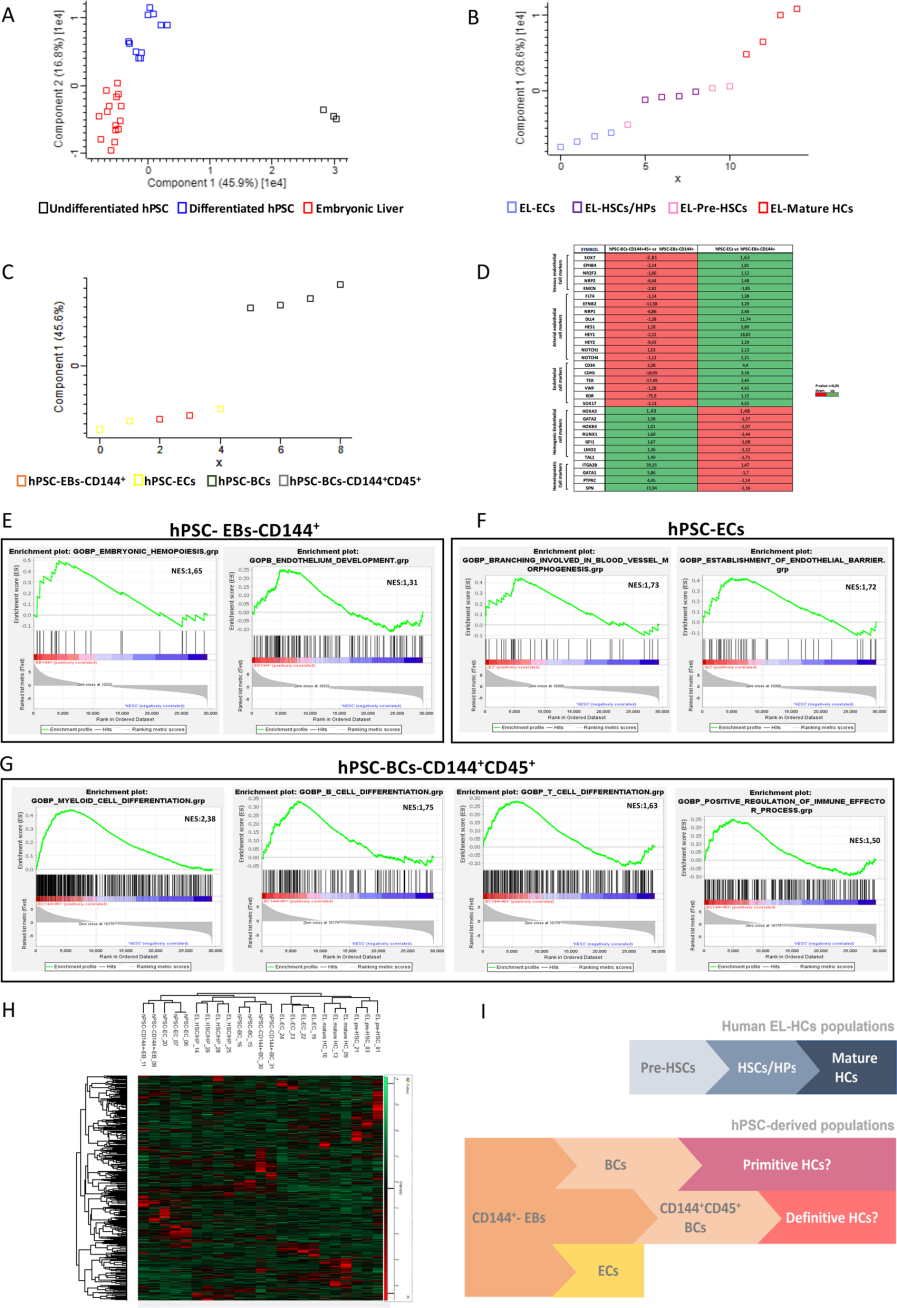
Transcriptomic analyses. **A**–**C** PCA loading plot of the first two principal components distribution of EL (red), differentiated hPSC (blue) and undifferentiated hPSC (black) (**A**). Distribution of EL-ECs (mauve), EL-HSCs/HPs (purple), EL-Pre-HSCs (pink) and EL-mature HCs (red) (**B**). Distribution of hPSC-derived populations, hPSC-BCs (blue), hPSC-BC-CD144+ CD45+ (grey), hPSC-EB-CD144^+^ (orange) and hPSC-ECs (yellow) (**C**), each square represents one sorted cell population. **D** Visualizing significant differential level expression of selected genes of hPSC-BC-CD144^+^CD45^+^ and hPSC-ECs between hPSC-EB-CD144^+^ revealing distinct endothelial, hemogenic endothelial and hematopoietic signatures. All fold-changes were calculated with respect to the mean value of hPSC-EB-CD144^+^. *P* value ≤ 0.05. **E**–**G** GSEA curves for the most significantly enriched hematopoietic and endothelial GO biological process in hPSC-EB-CD144^+^ (**E**), hPSC-ECs (**F**) and hPSC-BC-CD144+ CD45^+^ (**G**) compared to undifferentiated hPSCs. Enrichment analysis was performed using Gene Set Enrichment Analysis software v4.1.0 and probing the c5.go.bp.v7.4 collection of the Molecular Signatures Database (MSigDB). GO = Gene Ontology; NES = normalized enrichment score. *P* value ≤ 0.05. **H** Non-supervised hierarchical clustering of transcript (rows) and samples (columns) based on their distance using Pearson correlation. Color intensity indicates expression levels scaled to the column mean. The heatmap is coded red for increasing and green for decreasing. The colored bar on top indicates how the heat map colors are related to the standard score (*z*-score), i.e., the deviation from row mean in units of standard deviations above or below the mean. **I** Model summarizing transcriptomic results where hPSC-EB-CD144^+^ give rise to hPSC-ECs, hPSC-BCs and hPSC-BC-CD144^+^CD45^+^, these two latter containing primitive and definitive hematopoietic progenitors, respectively

We first confirmed the identity of the samples by a principal component analysis (PCA). As expected, undifferentiated hPSC- and hPSC-derived populations were grouped in two independent groups (Fig. 6A). In addition, hPSC-derived cells did not cluster with EL samples, yet they were spatially closer to EL than to hPSCs (Fig. 6A). Independent PCA within each group (EL- and hPSC-derived populations) clustered samples according to their endothelial and hematopoietic identity. As shown in Fig. 6B, EL sample distribution along the first component of EL-PCA showed a clear difference between EL-ECs and EL-mature HCs, located at the opposite extremes of the axis, while EL-pre-HSCs and EL-HSCs/HPs are in between these two populations, as a result of the EHT giving place to HCs. A similar analysis within hPSC-derived cells clustered hPSC-EB-CD144^+^ and hPSC-ECs in a separate group from hPSC-BC-CD144^+^CD45^+^ sorted and unsorted hPSC-BCs (Fig. 6C).

Supervised analysis performed to count and identify transcripts specifically up- or down-regulated in each pairwise cell population comparison confirmed these results. As attempted we identified much more differentially expressed genes (DEG) between hPSC-derived cell populations and undifferentiated hPSC (DEG range: min 17,292 and max 18,609), than between hPSC-derived cell populations themselves (DEG range: min 2361 and max 4602 D EG). When hPSC-derived cell populations were compared to EL cell population transcripts specifically up- or down-regulated in each pairwise cell population were comprised between 3098 and 8257 DEG and suggested similarities between them (Additional file 8: Fig. S8).

Regarding the list of genes differentially expressed, we confirmed the aforementioned RTqPCR and flow cytometry results, namely a decrease in the expression of pluripotency genes in all hPSC-derived populations together with an increased expression of hemato-endothelial genes in hPSC-EB-CD144^+^ compared to undifferentiated hPSC (Additional file 9: Fig. S9). As expected, hPSC-ECs compared hPSC-EB-CD144+, upregulated endothelial genes (e.g., *CDH5, KDR, CD34, VWF, TEK*), including arterial-specific genes (*e.g., SOX17, Notch1, NRP1*) and to a lesser extent, venous-related genes (*e.g., NRP2, NR2F2*). Conversely, hPSC-BCs upregulated hematopoietic genes compared to hPSC-EB-CD144^+^ (*e.g., PTPRC, SPN, ITGA2b*) (Additional file 9: Fig. S9) and also hemogenic endothelial genes as *TAL1*, *LMO2*, *GFI1*, *RUNX1*, *HOXB4* and *GATA2* (Fig. 6D). Although we did not include hPSC-BFU-E and CFU-GEMM populations in the transcriptome, mRNAs of embryonic (*ε*), fetal (*γ*) and adult (*β*) hemoglobin chains were detected in in hPSC-BCs and hPSC-BC-CD144+ CD45. The comparative analysis against EL-cell populations (*i.e.,* EL-pre-HSCs, EL-HPs/HSCs and mature EL-HCs) transcripts suggests that our differentiation protocol yields principally embryonic erythrocytes and to lesser extent, definitive erythrocytes (Additional file 9: Fig. S9).

Interestingly, although hPSC-EB-CD144^+^ generated both hPSC-ECs and hPSC-BC-CD144^+^CD45^+^ and shared the expression of CD144, they differed greatly in their gene signatures. Compared to hPSC-EB-CD144^+^, only hPSC-BC-CD144^+^CD45^+^ upregulated both hemogenic (*e.g., RUNX1, HOXB4)* and hematopoietic genes (*e.g., SPN, ITGA2B)* as shown in Fig. 6D.

To confirm these targeted genes analyses, we performed a Gene Set Enrichment Analysis (GSEA) between each hPSC-derived cell population and undifferentiated hPSCs. hPSC-EB-CD144^+^ expressed a discrete list of endothelial and hematopoietic-related terms (see Fig. 6E and Additional file 18: Table S9), including *Embryonic Hemopoiesis* and *Endothelium Development* ones. Endothelial terms were mainly enriched in hPSC-ECs and hPSC-EB-CD144^+^ compared to hPSC-BC-CD144^+^45^+^ and hPSC-BCs (Fig. 6E–G and Additional file 18: Table S9). In addition, hematopoietic terms including *Embryonic_Hemopoiesis* were enriched in hPSC-EB-CD144^+^, hPSC-BCs and hPSC-BC-CD144^+^45^+^ but not in hPSC-ECs (Fig. 6E–G and Additional file 18: Table S9). From all the hPSC-derived populations, hPSC-ECs contained the larger group of endothelial-related terms, including the following *Blood vessel morphogenesis*, *Endothelial P*ro*liferation*, *Lymphangiogenesis and Endothelial Barrier* (see Fig. 6F and Additional file 18: Table S9)*.* hPSC-ECs display also few terms involved in HSCs/HPs differentiation and term related to erythroid, myeloid, and megakaryocytic hematopoietic lineages. In contrast to hPSC-EC, hPSC-BCs and hPSC-BC-CD144^+^CD45^+^ contained a larger list of hematopoietic-related terms involved principally in the differentiation of HSCs/HPs and the maturation of HCS from erythroid, myeloid, megakaryocytes but also terms related to lymphoid lineages (see Fig. 6G and Additional file 18: Table S9). It is worth mentioning that the terms related to leukocytes, lymphocytes and immune system were largely more enriched in hPSC-BC-CD144^+^CD45^+^ than in unsorted hPSC-BC (Fig. 6G). These results suggest that hPSC-BC and more specifically hPSC-BC-CD144^+^CD45^+^ have the potential to generate multilineage hematopoiesis contrary to hPSC-EB-CD144^+^ that were only enriched in terms involved in erythroid, myeloid and megakaryocytic lineages (see Additional file 18: Table S9).

Once confirmed the identity of hPSC-derived populations, we performed an unsupervised hierarchical clustering between the hPSC-derived populations and the EL populations. As illustrated in Fig. 6H, two main clusters were obtained coinciding with the origin of the samples and not with their identity (*e.g.* endothelial or hematopoietic). An exception was observed for EL-HSCs/HPs, which were clustered together with non-sorted hPSC-BCs and hPSC-BC-CD144^+^CD45^+^, suggesting that these two hPSC-derived populations are closed to physiological EL-HSCs/HPs (Fig. 6H).

We then performed a GSEA between hPSC-derived populations and their closest EL-equivalent population. Compared to EL-pre-HSCs, hPSC-EB-CD144^+^ displayed a stronger endothelial signature. However, they were also enriched in a discrete list of terms related to EL-HSCs/HPs differentiation. This hematopoietic potential was further confirmed by GSEA between hPSC-EB-CD144^+^ and EL-ECs. Interestingly, we did not observe enriched terms related to mature hematopoietic lineages (Additional file 19: Table S10).

When hPSC-ECs were compared to EL-ECs, hPSC-ECs exhibited a more mature endothelial phenotype compared to EL-ECs since they were enriched on terms associated with endothelial function so as *platelet aggregation* or *myeloid-leukocyte migration* but also on terms related to HSCs/HPs differentiation (Additional file 19: Table S10).

Although comparison between hPSC-derived populations and undifferentiated hPSC showed that hPSC-BCs have a hematopoietic signature, GSEA between hPSC-BCs and EL-HSCs/HPs revealed that hPSC-BCs were enriched in endothelial terms when compared to human EL-HSCs/HPs hematopoietic populations. Furthermore, hPSC-BCs were not enriched in hematopoietic multilineage-related terms when compared to EL-HSCs/HPs. In contrast, hPSC-BC-CD144^+^-CD45^+^ exhibit a long list of terms related to multilineage hematopoietic progenitors when compared to EL-HSCs/HPs. Regarding their endothelial phenotype, hPSC-BC-CD144^+^-CD45^+^ expressed more endothelial-related terms than EL-HSCs/HPs but surprisingly less than EL-pre-HSC (Additional file 19: Table S10). These results suggest hPSC-BC-CD144^+^-CD45^+^ are more engaged towards hematopoietic lineages and have a largest multilineage potential compared to hPSC-BC and may correspond to a population in between EL-pre-HSCs and EL-HSCs/HSCs.

Thus, these transcriptomic analyses suggest that hPSC differentiation into endothelial and hematopoietic cells recapitulates the human hematopoietic developmental processes through an EHT leading first to primitive hematopoiesis progenitors and thereafter to definitive hematopoietic progenitors (Fig. 6I).

## Discussion

In the last decades, an increasing need to generate in vitro multicellular entities that recapitulate better organ physiology has pointed out the importance of ECs and HCs in these models. These two populations develop in close relationship during human embryonic development and maintain a tight connection during adulthood. Despite this, hPSC protocols focus on the differentiation into these lineages independently. Although most of the differentiation protocols of hPSCs into HCs generate an intermediate hemogenic endothelium, they do not generate mature ECs in vitro. Here we modified a previously published protocol of hematopoietic hPSC differentiation [36] by adding a WNT agonist, CHIR, during the first 48 h of differentiation. Early activation of the WNT canonical pathway has been proven to support definitive hematopoiesis [45]. After only 84 h of hPSC differentiation, we generated around 16% of a population of hematoendothelial cells. Recent hPSC-endothelial differentiation protocols reported an efficiency higher than 70% after 6 days of differentiation [28, 46, 47]. Protocols for the derivation of a hemogenic endothelium obtained a lower efficiency of around 30% after several days of differentiation [5, 42, 48]. The early cell isolation in our protocol could probably be in detriment of the obtention of a higher percentage of CD144+ ECs but assures the possibility to isolate progenitors of both endothelial and hematopoietic lineages. Further studies to increase the efficacy of our protocol may also contribute to the understanding of mechanisms underlying endothelial and hematopoietic specification. We showed that CD144 is a more suitable marker than CD309 to isolate a population of endothelial progenitor-like cells capable of generating both hematopoietic and endothelial cells, characterized for the expression of other hematoendothelial markers such as CD34, CD143 and CD309 [36, 49, 50] and hematoendothelial transcription factors such as *ETV2, RUNX1, GATA2, SCL* and *HOXA3*. In fact, hPSC-EB-CD144^+^ can be frozen and/or differentiated further into mature ECs or HCs. Mature hPSC-ECs derived from these hematoendothelial progenitors were able to take up LDL, activated upon pro-inflammatory signals and synthesized NO in vitro. We demonstrated that EC differentiation can be guided in vitro to arterial or venous lineages. In vivo*,* they incorporated to host blood vessels as we showed in a murine model of ischemia on a dorsal skinfold chamber. These results are similar to those previously obtained by our team using umbilical cord ECFCs [51]. hPSC-ECs seem also to facilitate new blood vessel formation 1 month post-injection, as evidenced by a slightly increase in the number of blood vessels compared to the control mouse injected with PBS. Ischemia did not seem to be necessary for the incorporation of new blood vessels since we observed human CD144^+^ cells in both conditions. This may be caused by natural turnover of the host endothelial layer and replacement by hPSC-ECs or by the wound-healing process following the surgical procedure leading to the formation of new blood vessels.

We showed hPSC-EB-CD144^+^ contained also a hemogenic endothelium able to differentiate into hPSC-BCs and, through these, generate erythroid and myeloid colonies. hPSC-BCs display a hematopoietic phenotype expressing CD43 and contained subpopulations expressing CD45, CD41, CD34 and/or CD143, which may correspond to different HP populations. As observed in the CFC assay, only a minority (< 1%) of colonies corresponded to GEMM, suggesting the existence of very few multipotent HPs in hPSC-BCs. These CFU-GEMM and BFU-E expressed high levels of ε-globulins as expected from primitive HPs. However, adult β-globin transcripts in these colonies besides the expression of genes involved in definitive hematopoiesis such as *RUNX1* (and the isoform c), *GATA2* and *HOXA3* suggest that hPSC-EB-CD144^+^ may also be able to generate definitive hematopoietic progenitors [52–54]. Other studies generating definitive HPs from hPSCs require longer time of differentiation [52, 55] or coculture with stromal cells [46, 47]. Since differentiation of hPSC-EB-CD144^+^ into hPSC-BCs may not favor the maintenance of multipotent HPs, we injected hPSC-EB-CD144^+^ and not hPSC-BCs in irradiated NSG mouse. We did not detect any human cells in mice that were injected intravenously. Instead, we were able to detect very few human CD45^+^ cells in the BM of mice-injected hPSC-EB-CD144^+^ by IBM injections. These results confirm that hPSC-EB-CD144^+^ may generate definitive progenitors under the right conditions but highlight the impossibility of these cells to migrate towards the hematopoietic niche. This is not a rare event since it has been previously documented that human hemogenic endothelium requires in vitro coculture with stromal cells in order to reconstitute hematopoiesis in host irradiated mouse [11, 48].

Despite all hPSC lines differentiate into endothelial and hematopoietic differentiation, we observed differences among them in terms of proliferation and response to DAPT in the case of hPSC-ECs, or expression of hematopoietic markers and CFC composition, in the case of hPSC-BCs. Many studies have reported differences among hPSCs lines, hESCs and hiPSCs [56–58]. Our protocol is robust enough to generate both lineages in an efficient way from different hPSCs but allows variability among individuals, which might be useful in fields such as developmental biology.

Transcriptomic analyses between hPSC-derived populations and undifferentiated hPSCs, between hPSC-derived populations themselves and against human EL-populations, confirmed our results. hPSC-EB-CD144^+^ display an hematoendothelial signature and were more enriched in endothelial terms than EL-pre-HSCs, as expected from a hemogenic endothelium. hPSC-ECs derived from hPSC-EB-CD144^+^ did show characteristics of mature EL-ECs. Interestingly, hPSC-ECs expressed genes involved in lymphangiogenesis, suggesting hPSC-ECs may either contain progenitors of lymphatic ECs or retain certain plasticity allowing them to differentiate further in lymphatic ECs. In addition, GSEA showed hPSC-ECs expressed genes related to HPs and hematopoiesis. Further analyses are required to determine whether this transcriptomic signature is a remnant of the hematoendothelial progenitor which they are differentiated from (hPSC-EB-CD144^+^) or evidence of their supportive role in hematopoietic differentiation as in the hematopoietic niche [17, 46].

Regarding hPSC-BCs, comparison between hPSCs-derived populations showed that hPSC-BCs displayed a hematopoietic signature as it has been demonstrated in our study and by other authors [59, 60]. hPSC-BCs were enriched in genes participating in embryonic hematopoiesis restricted to megakaryocytic, myeloid and erythroid lineages. Remarkably, hPSC-BCs were similarly enriched in endothelial-related terms compared to EL-pre-HSCs. At the end of the twentieth century and beginning of the twenty-first century, several studies pointed out the bipotency of hPSC-BCs [36, 49, 59, 60], able to generate both endothelial and hematopoietic lineages. In 2009, Lancrin et al. demonstrated in mice that hPSC-BCs generate HCs through an endothelial intermediate [59]. Our study demonstrated that hPSC-BCs are a heterogeneous population which contain a small fraction of CD144^+^CD45^+^ cells. Rather than an intermediate of the differentiation of hPSC-EB-CD144^+^ into hPSC-BCs (hPSC-EB-CD144^+^  > hPSC-BC-CD144^+^CD45^+^  > hPSC-BC-CD144^−^CD45^+^), our transcriptomic analyses suggest that hPSC-BC-CD144^+^CD45^+^ are more engaged in hematopoiesis and contain more multilineage progenitors involved in the differentiation of definitive HCs such as lymphoid cells than hPSC-BCs and that they may correspond to a population in between EL-pre-HSCs and EL-HSCs/HSCs. Thus, our hematopoietic differentiation protocol of hPSC-EB-CD144^+^ seems to recapitulate in vitro both primitive and definitive hematopoiesis. Isolation and expansion of these hPSC-BC-CD144^+^CD45^+^ subpopulation should be explored in the future as a potential source of human HSCs.

## Conclusion

In conclusion, herein we presented a differentiation protocol of hematopoietic and endothelial cells through a hematoendothelial population. This intermediate population is obtained after 84 h of hPSC-EB formation following CD144-based cell sorting. These cells can be frozen without altering their bipotential properties. Their endothelial and hematopoietic progeny are considered as pure because they are not mixed populations of both lineages. This protocol was shown to be reproducible; hPSC-ECs and hPSC-BC-CD144^+^CD45^+^ were obtained independently on the hESC and hiPSC used. Comparison of hPSC-derived cells with human EL subpopulations showed similarities between these hPSC-derived cells and their physiological human EL counterpart and help to define a developmental hierarchy between hPSC-derived cells themselves. Finally, hPSC-ECs and hPSC-BC-CD144+CD45+ subpopulation were proven to be good candidates for future cell therapy. Therefore, hPSC-EB-CD144^+^ are an interesting and rapid source of ECs and HCs for tissue engineering.

## Supplementary Information

**Additional file 1.** **Supplementary figure 1.** (**A**) Representative image of the flow cytometry analysis of 84h- hPSC-EBs for hematoendothelial (CD309, CD143 and CD34), endothelial (CD144 and CD31) and hematopoietic markers (CD43, CD45 and CD41) for every hPSC line. (**B**) Representative analysis of hematoendothelial, endothelial and hematopoietic markers within the positive population for CD144, CD143, CD309, CD34 and CD31 for A29-EBs.

**Additional file 2.** **Supplementary figure 2.** (**A**) Representative phase-contrast images of hPSC-derived endothelial cells (hPSC-ECs) from passage (p) 1 to 5 derived from H1-CD144+-EBs. Scale bar 500 μm. (**B**) Representative flow cytometry histograms of hPSC-ECs from A29 (top), SA01 (center) and H1 (below) cell lines at p1 and p3 for the expression of hematoendothelial (CD309, CD143 and CD34), endothelial (CD144 and CD31) and hematopoietic markers (CD43, CD45 and CD41).

**Additional file 3.** **Supplementary figure 3.** Arterial and vein differentiation of hPSC-ECs. (**A**) Differentiation scheme of hPSC-EB-CD144+ into hPSC-ECs in the presence or absence of DAPT and representative phase-contrast microscopy images after differentiation. Scale Bar 500 μm. Flow cytometry analysis of the endothelial phenotype of hPSC-ECs in the presence and absence of DAPT. (**B**) Confocal immunofluorescent image of Matrigel networks formed by hPSC-ECs. CD31 in green and DAPI in blue. Scale Bar: 100 μm.

**Additional file 4.** **Supplementary figure 4.** (**A**) Representative histograms of the flow cytometry analysis of eNOS expression in hPSC-ECs and ECFC (**B**) Representative histograms of the flow cytometry analysis of the probe DAF-FM for NO detection in hPSC-EC and ECFC in the presence and absence of LPS and SNAP.

**Additional file 5.** **Supplementary figure 5.** (**A**) Representative macroscopic images (left panels) of a mouse dorsal chamber without (superior panel) and with ischemia (inferior panel) at day 5 and day 29 post-injection of hPSC-ECs. Right panels show the corresponding treated images used for quantification of the area occupied by blood vessels. (**B**) Representative confocal image at day 19 post-injection of a mouse dorsal skinfold chamber with ischemia, injected with mcherry-hPSC-ECs (red, white arrows). Vessel perfusion was observed thanks to the injection of dextran 70 kDa-FITC (green). Scale bar: 200 μm.

**Additional file 6.** **Supplementary figure 6.** Representative flow cytometry histograms and dot plots of hPSC-BCs from A29 (top), SA01 (center) and H1 (below) cell lines for the expression of hematoendothelial (CD309, CD143 and CD34), endothelial (CD144 and CD31) and hematopoietic markers (CD43, CD45 and CD41) as (**A**) simple and (**B**) double labeling.

**Additional file 7.** **Supplementary figure 7.** Relative mRNA expression of the hemoglobin subunit epsilon-1 (HBE1), gamma-1 (HBG1) and beta-1 (HBB1) in the BFU-E and CFU-GEMM derived from every hPSC-cell line compared to mRNAs from human embryonic liver.

**Additional file 8.** **Supplementary figure 8.** Counting of differential transcripts between undifferentiated hESCs, differentiated hESCs and EL cell populations. P<0.05 and -1.5<foldchange<1.5.

**Additional file 9.** **Supplementary figure 9.** (**A**) Heatmap depicting mean level expression of POU5F1, NANOG, KDR, CDH5, CD34, SPN, ITGA2B and PTPRC genes in undifferentiated hPSC (H1 cell line), in hPSC-EB-CD144^+^, hPSC-BCs and hPSC-BC-CD144^+^CD45^+^ and hPSC-ECs derived populations obtained by gene microarray analysis (GeneChip® HumanGene 2.0 ST; Affymetrix). Mean level expression of selected genes are also visualizing in CD144^+^CD45-CD34^+^ EL-EC, CD144^+^CD45^low^CD34^+^ EL-pre-HSC, CD144-CD45^low^CD34^+^ EL-HSC/HP and CD144-CD45^high^CD34-EL-mature HC EL cell subpopulations for comparison. Heatmap revealed the loss of pluripotency during hPSC-EB-CD144^+^ formation and subsequent hPSC- BCs and hPSC-ECs derivation and distinct endothelial and hematopoietic signatures in hPSC-EB-CD144^+^, hPSCBCs, hPSC-BC-CD144^+^CD45^+^ and hPSC-ECs derived populations. (**B**) Heatmap depicting mean level expression of embryonic HBE1 (Hemoglobin ε), fetal HBG1 (Hemoglobin γ), and adult HBB (hemoglobin β) genes in undifferentiated hPSC (H1 cell line), in hPSC-EB-CD144^+^, hPSC-BCs and hPSC-BC-CD144^+^CD45^+^ and hPSC-ECs derived populations obtained by gene microarray analysis (GeneChip^®^ HumanGene 2.0 ST; Affymetrix). Mean level expression of selected genes are also visualizing in CD144^+^CD45^low^CD34^+^ EL-pre-HSC, CD144-CD45^low^CD34^+^ EL-HSCs/HPs and CD144-CD45^high^CD34- EL-mature HCs EL cell subpopulations for comparison. Heatmap revealed that hPSC-BCs, hPSC-BC-CD144^+^CD45^+^ contained principally embryonic erythrocytes and to lesser extent definitive erythrocytes. Undifferentiated hESC (H1) , hESC-derived cell populations (n=3) and EL cell populations (n=4) After normalization, for each cell population, a Tukey's Biweight Robust Mean is calculated from log2 expression values. For each gene analyzed the most highly expressed transcript, is in red, the lowest level expressed transcript is in green. ID, Identifiant Affymetrix; Undiff, undifferentiated.

**Additional file 10. Supplementary table 1.** List of antibodies used in flow cytometry and immunofluorescence analysis.

**Additional file 11.** **Supplementary table 2.** Oligonucleotides used in RTqPCR experiments for hPSC-EBs, hPSC-ECs and hPSC-BCs.

**Additional file 12.** **Supplementary table 3.** Oligonucleotides used in RTqPCR experiments in artery and vein differentiation.

**Additional file 13.** **Supplementary table 4.** Oligonucleotides used in qPCR for the detection of human DNA.

**Additional file 14.** **Supplementary table 5.** Average percentage ± SD of positive cells for the endothelial and hematopoietic markers analyzed by flow cytometry in hPSC-EBs. Supplementary to Figure 1F.

**Additional file 15.** **Supplementary table 6.** Average percentage ± SD of positive cells for the endothelial and hematopoietic markers analyzed by flow cytometry in hPSC-ECs during consecutive passages. Supplementary to Figure 2D.

**Additional file 16.** **Supplementary table 7.** Summary of average ± SD values of in vitro endothelial characterization tests: Matrigel tube formation assay, TNFα activation assay, eNOS expression and NO production. Supplementary to Figure 3A-D.

**Additional file 17.** **Supplementary table 8.** Average percentage of positive cells for the endothelial and hematopoietic markers analyzed by flow cytometry in hPSC-BCs. Supplementary to Figure 4B-C.

**Additional file 18.** **Supplementary table 9.** Contains a list of significantly hematopoietic and endothelial Gene Ontology Biological Process enrichment in hPSC-EB-CD144+, hPSC-BCs, hPSC-BC-CD144+CD45+ and hPSC-ECs vs undifferentiated hESC. Enrichment analysis was performed using Gene Set Enrichment Analysis software v4.1.0 and probing the c5.go.bp.v7.4 collection of the Molecular Signatures Database (MSigDB ). Number of genes involved in the gene set, Enrichment score (ES), normalized enrichment score (NES) and nominal P-value are given in the supplementary Table. All selected Gene Ontology Biological Process enrichment show a P value ≤ 0.05.

**Additional file 19.** **Supplementary table 10.** Contains list of significantly hematopoietic and endothelial Gene Ontology Biological Process enrichment in hPSC-EB-CD144+, hPSC-BCs, hPSC-BC-CD144+CD45+ and hPSC-ECs compare to EL cell populations. Enrichment analysis was performed using Gene Set Enrichment Analysis software v4.1.0 and probing the c5.go.bp.v7.4 collection of the Molecular Signatures Database (MSigDB). Number of genes involved in the gene set, Enrichment score (ES), normalized enrichment score (NES) and nominal P-value are given in the supplementary Table 6. All selected Gene Ontology Biological Process enrichment show a P value ≤ 0.05.

## Data Availability

All materials are available by the corresponding author: alejandra.vargas@uclouvain.be.
