# Supplementary material for: Endothelial and hematopoietic hPSCs differentiation via a hematoendothelial progenitor
Source: Stem Cell Res Ther. 2022 Jun 17;13:254. doi: 10.1186/s13287-022-02925-w (PMC9205076; doi:10.1186/s13287-022-02925-w)
Supplement: Supplementary file 1 — Additional file 1. Supplementary figure 1. (A) Representative image of the flow cytometry analysis of 84h- hPSC-EBs for hematoendothelial (CD309, CD143 and CD34), endothelial (CD144 and CD31) and hematopoietic markers (CD43, CD45 and CD41) for every hPSC line. (B) Representative analysis of hematoendothelial, endothelial and hematopoietic markers within the positive population for CD144, CD143, CD309, CD34 and CD31 for A29-EBs. [file 13287_2022_2925_MOESM1_ESM.pdf]

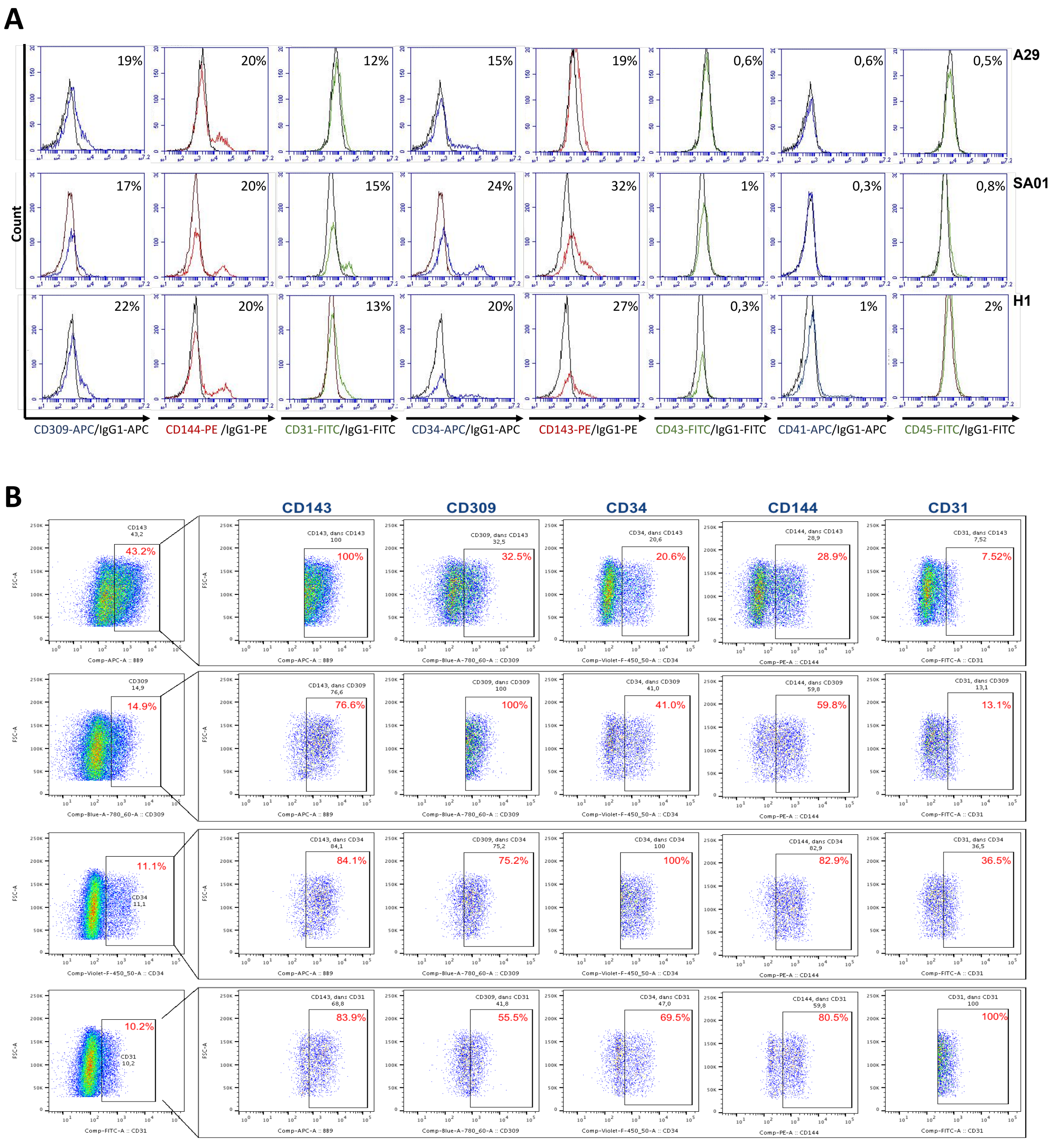


**Supplementary figure 1.** (**A)** Representative image of the flow cytometry analysis of 84h-hPSC-EBs for hematoendothelial (CD309, CD143 and CD34), endothelial (CD144 and CD31) and hematopoietic markers (CD43, CD45 and CD41) for every hPSC line (**B)** Representative analysis of hematoendothelial, endothelial and hematopoietic markers within the positive population for CD144, CD143, CD309, CD34 and CD31 for A29-EBs.
