# Supplementary material for: Endothelial and hematopoietic hPSCs differentiation via a hematoendothelial progenitor
Source: Stem Cell Res Ther. 2022 Jun 17;13:254. doi: 10.1186/s13287-022-02925-w (PMC9205076; doi:10.1186/s13287-022-02925-w)
Supplement: Supplementary file 2 — Additional file 2. Supplementary figure 2. (A) Representative phase-contrast images of hPSC-derived endothelial cells (hPSC-ECs) from passage (p) 1 to 5 derived from H1-CD144+-EBs. Scale bar 500 μm. (B) Representative flow cytometry histograms of hPSC-ECs from A29 (top), SA01 (center) and H1 (below) cell lines at p1 and p3 for the expression of hematoendothelial (CD309, CD143 and CD34), endothelial (CD144 and CD31) and hematopoietic markers (CD43, CD45 and CD41). [file 13287_2022_2925_MOESM2_ESM.pdf]

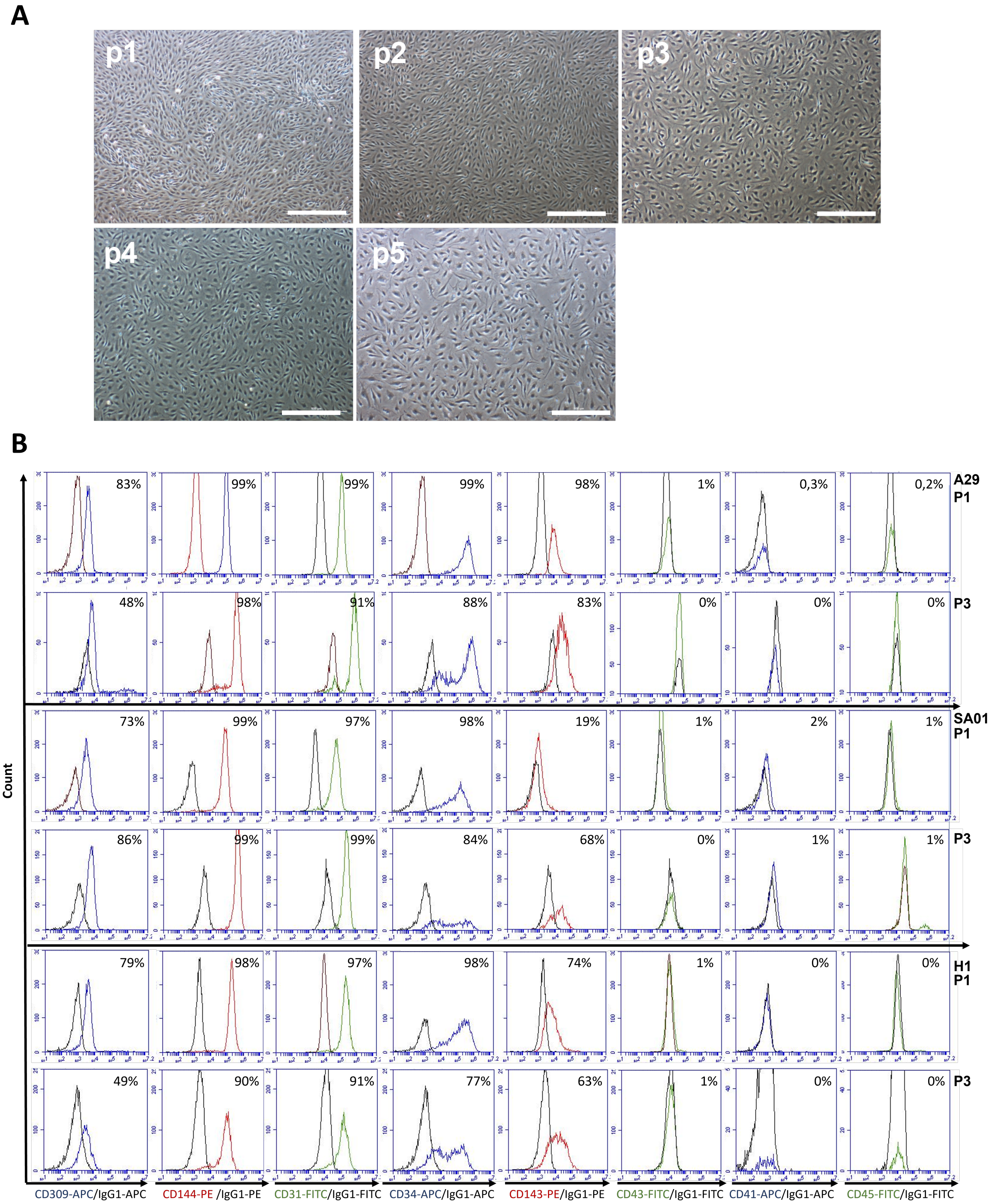

Supplementary figure 2. (A) Representative phase-contrast images of hPSC-derived endothelial cells (hPSC-ECs) from passage (p) 1 to 5 derived from H1-CD144$^+$-EBs. Scale bar 500 µm. **(B)** Representative flow cytometry histograms of hPSC-ECs from A29 (top), SA01 (center) and H1 (below) cell lines at p1 and p3 for the expression of hematoendothelial (CD309, CD143 and CD34), endothelial (CD144 and CD31) and hematopoietic markers (CD43, CD45 and CD41).
