# Supplementary material for: Endothelial and hematopoietic hPSCs differentiation via a hematoendothelial progenitor
Source: Stem Cell Res Ther. 2022 Jun 17;13:254. doi: 10.1186/s13287-022-02925-w (PMC9205076; doi:10.1186/s13287-022-02925-w)
Supplement: Supplementary file 3 — Additional file 3. Supplementary figure 3. Arterial and vein differentiation of hPSC-ECs. (A) Differentiation scheme of hPSC-EB-CD144+ into hPSC-ECs in the presence or absence of DAPT and representative phase-contrast microscopy images after differentiation. Scale Bar 500 μm. Flow cytometry analysis of the endothelial phenotype of hPSC-ECs in the presence and absence of DAPT. (B) Confocal immunofluorescent image of Matrigel networks formed by hPSC-ECs. CD31 in green and DAPI in blue. Scale Bar: 100 μm. [file 13287_2022_2925_MOESM3_ESM.pdf]

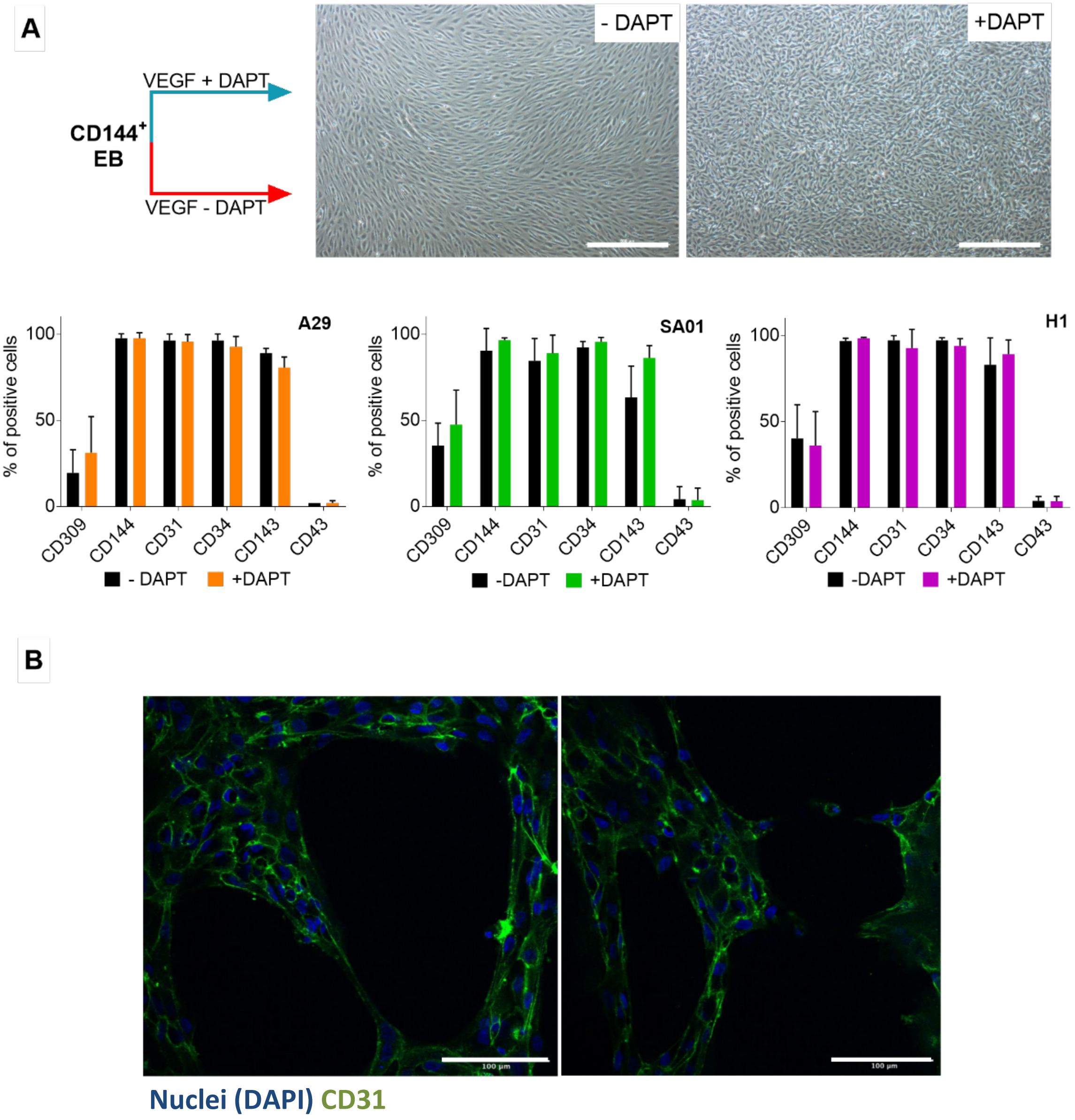

Supplementary figure 3. Arterial and vein differentiation of hPSC-ECs. (**A)** Differentiation scheme of hPSC-EB-CD144^{+} into hPSC-ECs in the presence or absence of DAPT and representative phase-contrast microscopy images after differentiation. Scale Bar 500 µm. Flow cytometry analysis of the endothelial phenotype of hPSC-ECs in the presence and absence of DAPT. (**B)** Confocal immunofluorescent image of Matrigel networks formed by hPSC-ECs. CD31 in green and DAPI in blue. Scale Bar: 100 µm
