# Supplementary material for: Endothelial and hematopoietic hPSCs differentiation via a hematoendothelial progenitor
Source: Stem Cell Res Ther. 2022 Jun 17;13:254. doi: 10.1186/s13287-022-02925-w (PMC9205076; doi:10.1186/s13287-022-02925-w)
Supplement: Supplementary file 4 — Additional file 4. Supplementary figure 4. (A) Representative histograms of the flow cytometry analysis of eNOS expression in hPSC-ECs and ECFC (B) Representative histograms of the flow cytometry analysis of the probe DAF-FM for NO detection in hPSC-EC and ECFC in the presence and absence of LPS and SNAP. [file 13287_2022_2925_MOESM4_ESM.pdf]

.

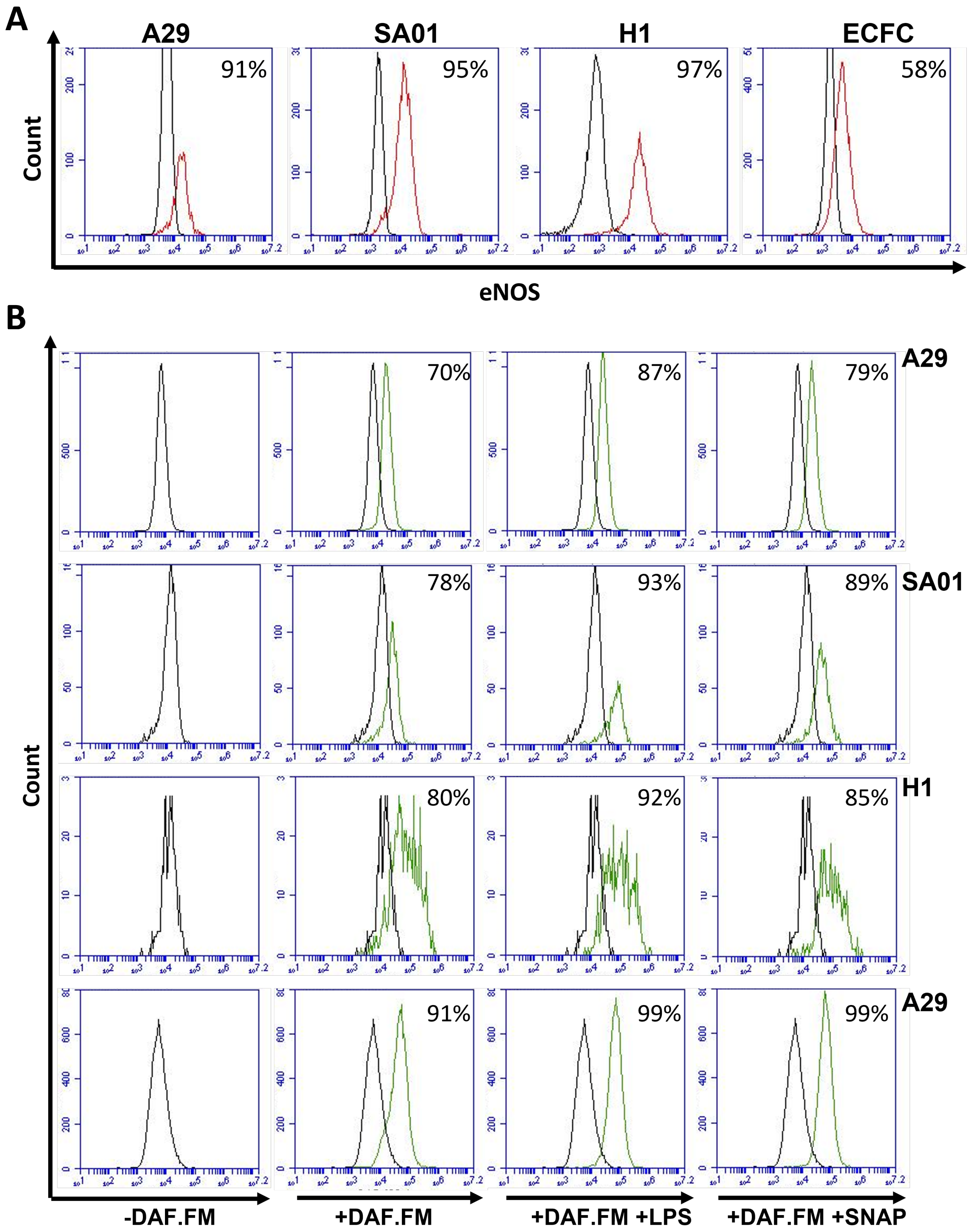

Supplementary figure 4. **(A)** Representative histograms of the flow cytometry analysis of eNOS expression in hPSC-ECs and ECFC **(B)** Representative histograms of the flow cytometry analysis of the probe DAF-FM for NO detection in hPSC-EC and ECFC in the presence and absence of LPS and SNAP.
