# Supplementary material for: Endothelial and hematopoietic hPSCs differentiation via a hematoendothelial progenitor
Source: Stem Cell Res Ther. 2022 Jun 17;13:254. doi: 10.1186/s13287-022-02925-w (PMC9205076; doi:10.1186/s13287-022-02925-w)
Supplement: Supplementary file 5 — Additional file 5. Supplementary figure 5. (A) Representative macroscopic images (left panels) of a mouse dorsal chamber without (superior panel) and with ischemia (inferior panel) at day 5 and day 29 post-injection of hPSC-ECs. Right panels show the corresponding treated images used for quantification of the area occupied by blood vessels. (B) Representative confocal image at day 19 post-injection of a mouse dorsal skinfold chamber with ischemia, injected with mcherry-hPSC-ECs (red, white arrows). Vessel perfusion was observed thanks to the injection of dextran 70 kDa-FITC (green). Scale bar: 200 μm. [file 13287_2022_2925_MOESM5_ESM.pdf]

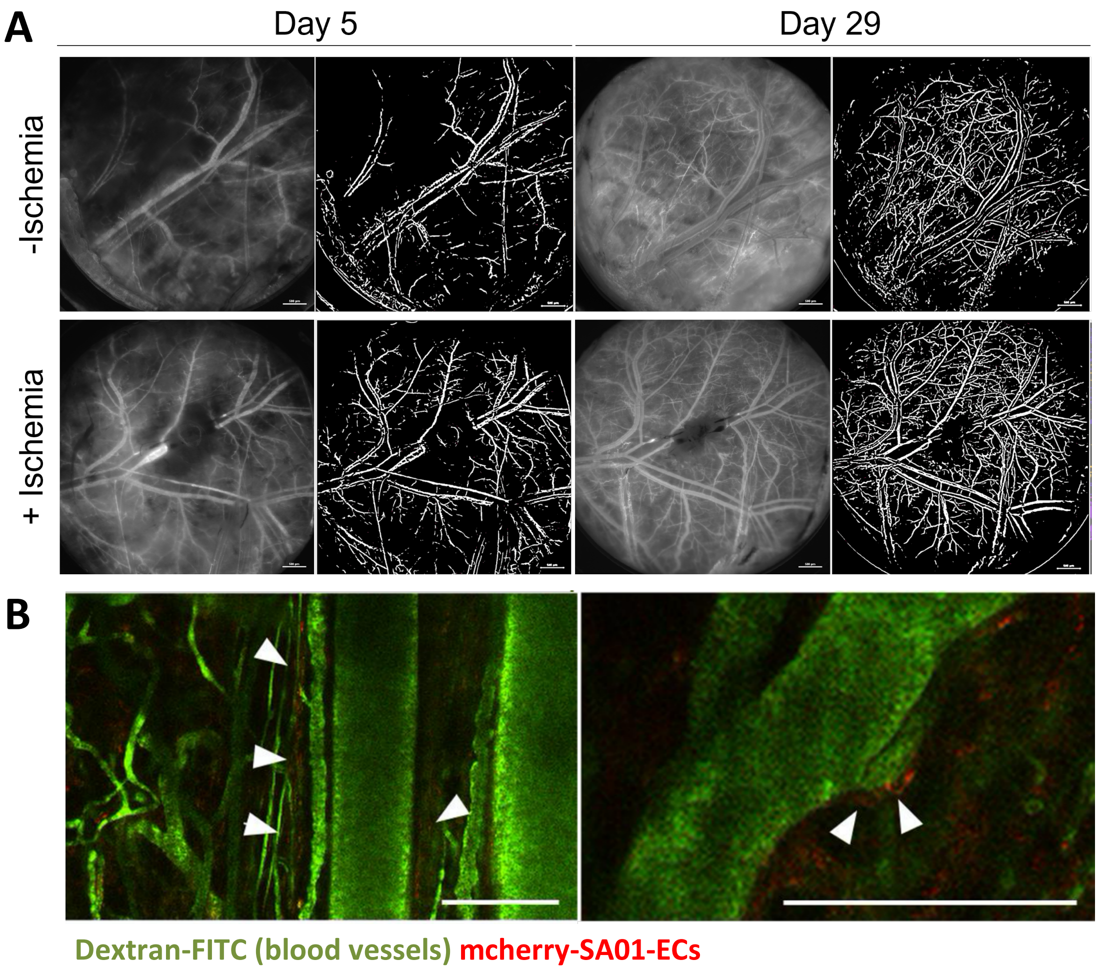

Supplementary figure 5. (A) Representative macroscopic images (left panels) of a mouse dorsal chamber without (superior panel) and with ischemia (inferior panel) at day 5 and day 29 post-injection of hPSC-ECs. Right panels show the corresponding treated images used for quantification of the area occupied by blood vessels. (**B)** Representative confocal image at day 19 post-injection of a mouse dorsal skinfold chamber with ischemia, injected with mcherry-hPSC-ECs (red, white arrows). Vessel perfusion was observed thanks to the injection of dextran 70 kDa-FITC (green). Scale bar: 200 µm.
