# Supplementary material for: Endothelial and hematopoietic hPSCs differentiation via a hematoendothelial progenitor
Source: Stem Cell Res Ther. 2022 Jun 17;13:254. doi: 10.1186/s13287-022-02925-w (PMC9205076; doi:10.1186/s13287-022-02925-w)
Supplement: Supplementary file 6 — Additional file 6. Supplementary figure 6. Representative flow cytometry histograms and dot plots of hPSC-BCs from A29 (top), SA01 (center) and H1 (below) cell lines for the expression of hematoendothelial (CD309, CD143 and CD34), endothelial (CD144 and CD31) and hematopoietic markers (CD43, CD45 and CD41) as (A) simple and (B) double labeling. [file 13287_2022_2925_MOESM6_ESM.pdf]

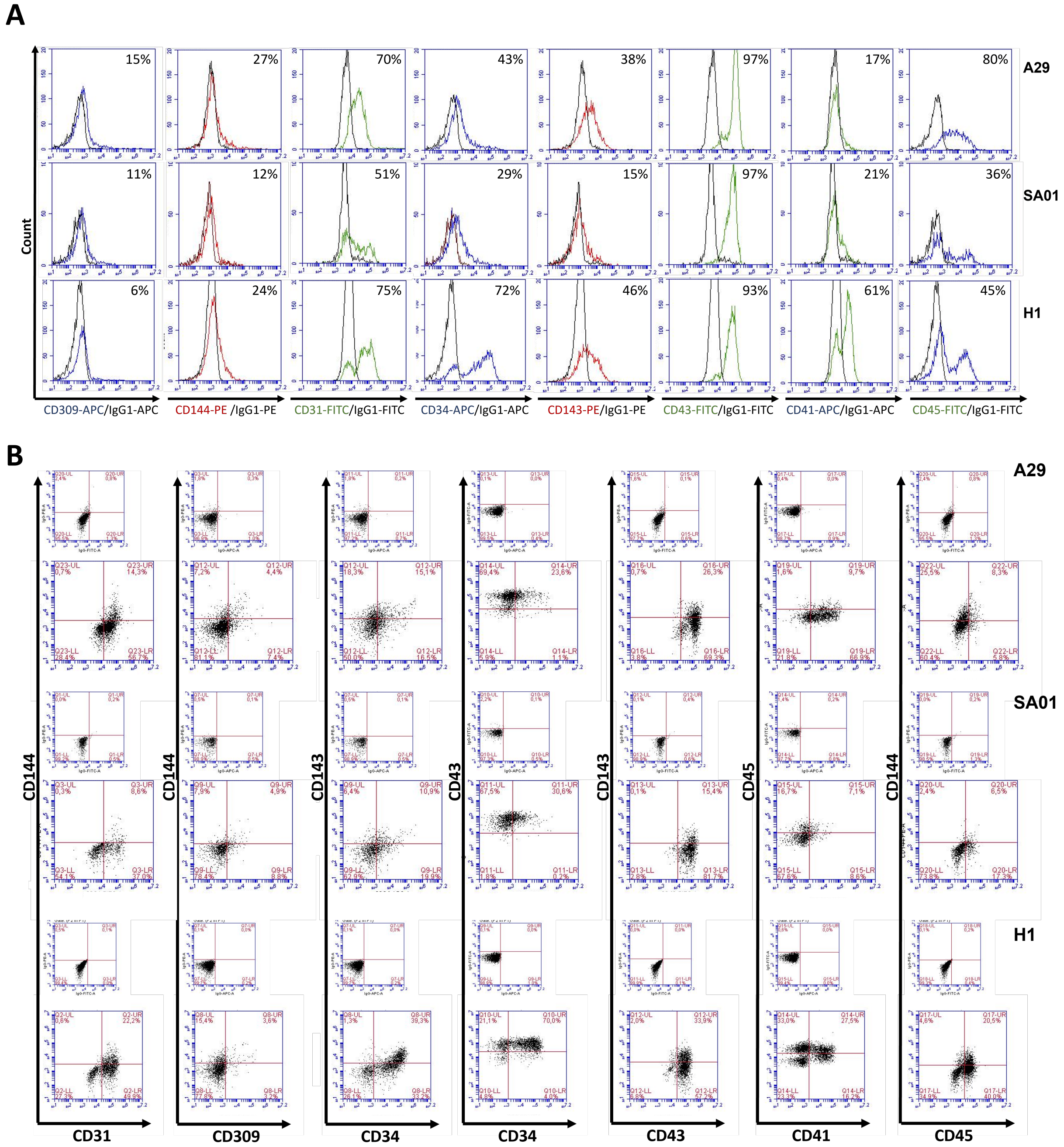

Supplementary figure 6. Representative flow cytometry histograms and dot plots of hPSC-BCs from A29 (top), SA01 (center) and H1 (below) cell lines for the expression of hematoendothelial (CD309, CD143 and CD34), endothelial (CD144 and CD31) and hematopoietic markers (CD43, CD45 and CD41) as (**A)** simple and (**B)** double labeling.
