# Supplementary material for: Endothelial and hematopoietic hPSCs differentiation via a hematoendothelial progenitor
Source: Stem Cell Res Ther. 2022 Jun 17;13:254. doi: 10.1186/s13287-022-02925-w (PMC9205076; doi:10.1186/s13287-022-02925-w)
Supplement: Supplementary file 7 — Additional file 7. Supplementary figure 7. Relative mRNA expression of the hemoglobin subunit epsilon-1 (HBE1), gamma-1 (HBG1) and beta-1 (HBB1) in the BFU-E and CFU-GEMM derived from every hPSC-cell line compared to mRNAs from human embryonic liver. [file 13287_2022_2925_MOESM7_ESM.pdf]

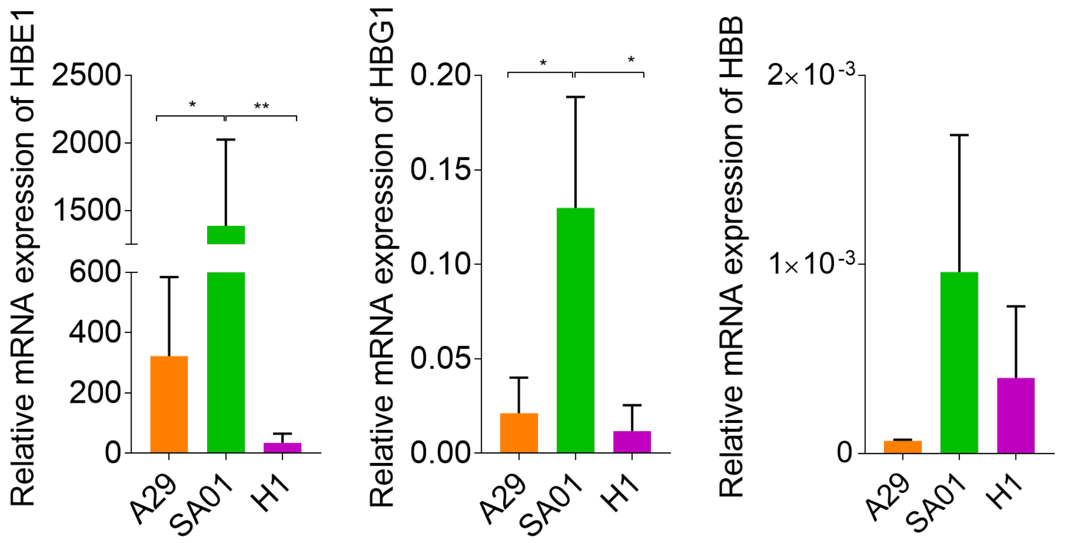

Supplementary figure 7. Relative mRNA expression of the hemoglobin subunit epsilon-1 (HBE1), gamma-1 (HBG1) and beta-1 (HBB1) in the BFU-E and CFU-GEMM derived from every hPSC-cell line compared to mRNAs from human embryonic liver.
