# Supplementary material for: Endothelial and hematopoietic hPSCs differentiation via a hematoendothelial progenitor
Source: Stem Cell Res Ther. 2022 Jun 17;13:254. doi: 10.1186/s13287-022-02925-w (PMC9205076; doi:10.1186/s13287-022-02925-w)
Supplement: Supplementary file 8 — Additional file 8. Supplementary figure 8. Counting of differential transcripts between undifferentiated hESCs, differentiated hESCs and EL cell populations. P<0.05 and -1.5<foldchange<1.5. [file 13287_2022_2925_MOESM8_ESM.pdf]

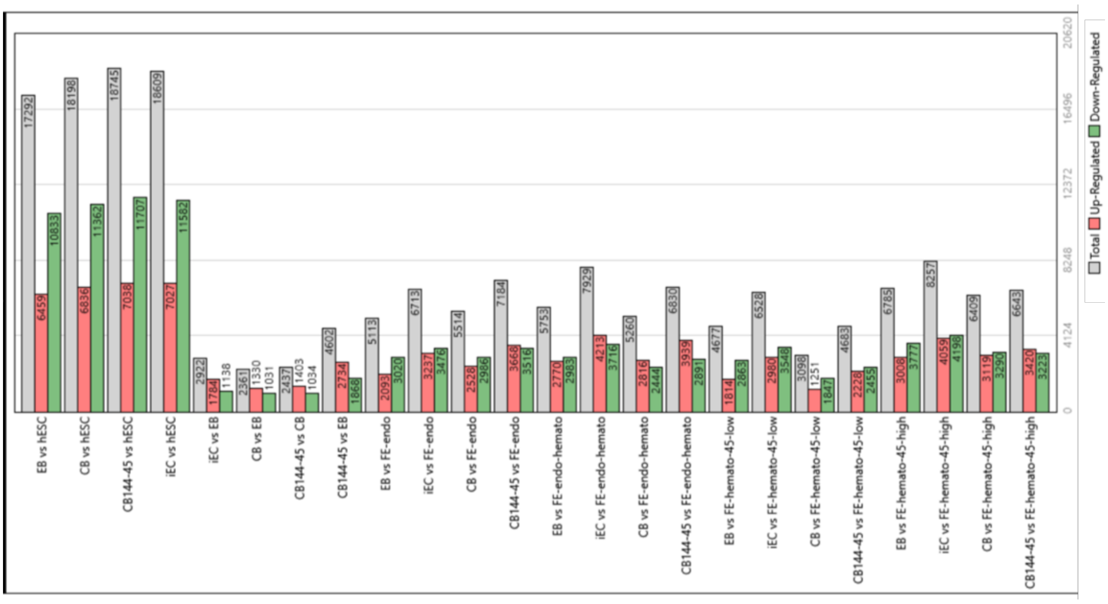

Supplementary figure 8. Counting of differential transcripts between undifferentiated hESCs, differentiated hESCs and EL cell populations. P<0.05 and -1.5<foldchange<1.5.
