# Supplementary material for: Endothelial and hematopoietic hPSCs differentiation via a hematoendothelial progenitor
Source: Stem Cell Res Ther. 2022 Jun 17;13:254. doi: 10.1186/s13287-022-02925-w (PMC9205076; doi:10.1186/s13287-022-02925-w)
Supplement: Supplementary file 9 — Additional file 9. Supplementary figure 9. (A) Heatmap depicting mean level expression of POU5F1, NANOG, KDR, CDH5, CD34, SPN, ITGA2B and PTPRC genes in undifferentiated hPSC (H1 cell line), in hPSC-EB-CD144+, hPSC-BCs and hPSC-BC-CD144+CD45+ and hPSC-ECs derived populations obtained by gene microarray analysis (GeneChip® HumanGene 2.0 ST; Affymetrix). Mean level expression of selected genes are also visualizing in CD144+CD45-CD34+ EL-EC, CD144+CD45lowCD34+ EL-pre-HSC, CD144-CD45lowCD34+ EL-HSC/HP and CD144-CD45highCD34-EL-mature HC EL cell subpopulations for comparison. Heatmap revealed the loss of pluripotency during hPSC-EB-CD144+ formation and subsequent hPSC- BCs and hPSC-ECs derivation and distinct endothelial and hematopoietic signatures in hPSC-EB-CD144+, hPSCBCs, hPSC-BC-CD144+CD45+ and hPSC-ECs derived populations. (B) Heatmap depicting mean level expression of embryonic HBE1 (Hemoglobin ε), fetal HBG1 (Hemoglobin γ), and adult HBB (hemoglobin β) genes in undifferentiated hPSC (H1 cell line), in hPSC-EB-CD144+, hPSC-BCs and hPSC-BC-CD144+CD45+ and hPSC-ECs derived populations obtained by gene microarray analysis (GeneChip® HumanGene 2.0 ST; Affymetrix). Mean level expression of selected genes are also visualizing in CD144+CD45lowCD34+ EL-pre-HSC, CD144-CD45lowCD34+ EL-HSCs/HPs and CD144-CD45highCD34- EL-mature HCs EL cell subpopulations for comparison. Heatmap revealed that hPSC-BCs, hPSC-BC-CD144+CD45+ contained principally embryonic erythrocytes and to lesser extent definitive erythrocytes. Undifferentiated hESC (H1) , hESC-derived cell populations (n=3) and EL cell populations (n=4) After normalization, for each cell population, a Tukey's Biweight Robust Mean is calculated from log2 expression values. For each gene analyzed the most highly expressed transcript, is in red, the lowest level expressed transcript is in green. ID, Identifiant Affymetrix; Undiff, undifferentiated. [file 13287_2022_2925_MOESM9_ESM.pdf]

A

ID	h-PSC-EB-144+ Avg (log2)	hPSC-BCs Avg (log2)	hPSC-BC-CD144+CD45+ Avg (log2)	hPSC-ECs Avg (log2)	Undiff hESC Avg (log2)	EL-ECs Avg (log2)	EL-pre-HSCs Avg (log2)	EL-HSCs/HPs Avg (log2)	EL-mature HCs Avg (log2)	Gene Symbol
16671784	5,47	5,17	5,24	5,3	6,5	5,2	6,2	5,57	5,65	POU5F1P4
16747852	2,14	2,48	2,4	2,24	2,65	1,96	1,78	1,96	1,99	NANOG
16976029	10,32	6,15	4,08	10,53	4,69	10,08	4	3,13	3,3	KDR
16819794	7,52	3,69	3,28	9,18	3,46	7,87	3,31	2,81	3	CDH5
16698801	7,13	6,05	8,32	9,27	4,25	7,03	7,72	6,62	3,54	CD34
16817624	3,49	6,63	7,48	3,27	4,36	4,79	5,99	7,57	6,16	SPN
16845681	3,36	7,9	8,65	3,91	4,88	3,64	7,41	6,04	4,07	ITGA2B
16675578	3,07	4	5,23	2,89	3,14	2,82	5,11	5,47	8,86	PTPRC

B

ID	hPSC-EB Avg (log2)	hPSC-BC Avg (log2)	hPSC-BC-CD144+CD45+ Avg (log2)	hPSC-ECs Avg (log2)	Undiff hPSC Avg (log2)	EL-pre-HSCs Avg (log2)	EL-mature HCs Avg (log2)	EL-HSCs/HPs Avg (log2)	Gene Symbol
16734877	6,76	10,53	9,87	2,92	4,42	6,98	7,42	6,84	HBE1
16734862	5,1	11,48	8,48	3,52	5,03	10,94	10,31	10,08	HBG1; BGLT3
16734840	2,61	2,8	2,7	2,58	2,76	4,15	3,67	3,35	HBB

Supplementary figure 9. (A) Heatmap depicting mean level expression of *POU5F1, NANOG, KDR, CDH5, CD34, SPN, ITGA2B* and *PTPRC* genes in undifferentiated hPSC (H1 cell line), in hPSC-EB-CD144^{+}, hPSC-BCs and hPSC-BC-CD144^{+}CD45^{+} and hPSC-ECs derived populations obtained by gene microarray analysis (GeneChip® HumanGene 2.0 ST; Affymetrix). Mean level expression of selected genes are also visualizing in CD144^{+}CD45^{-}CD34^{+} EL-EC, CD144^{+}CD45^{low}CD34^{+} EL-pre-HSC, CD144^{-}CD45^{low}CD34^{+} EL-HSC/HP and CD144^{-}CD45^{high}CD34^{-} EL-mature HC EL cell subpopulations for comparison. Heatmap revealed the loss of pluripotency during hPSC-EB-CD144+ formation and subsequent hPSC- BCs and hPSC-ECs derivation and distinct endothelial and hematopoietic signatures in hPSC-EB-CD144^{+}, hPSC-BCs, hPSC-BC-CD144^{+}CD45^{+} and hPSC-ECs derived populations.

(B) Heatmap depicting mean level expression of embryonic *HBE1* (Hemoglobin ε), fetal *HBG1* (Hemoglobin γ), and adult *HBB* (hemoglobin β) genes in undifferentiated hPSC (H1 cell line), in hPSC-EB-CD144^{+}, hPSC-BCs and hPSC-BC-CD144^{+}CD45^{+} and hPSC-ECs derived populations obtained by gene microarray analysis (GeneChip® HumanGene 2.0 ST; Affymetrix). Mean level expression of selected genes are also visualizing in CD144^{+}CD45^{low}CD34^{+} EL-pre-HSC, CD144^{-}CD45^{low}CD34^{+} EL-HSCs/HPs and CD144^{-}CD45^{high}CD34^{-} EL-mature HCs EL cell subpopulations for comparison. Heatmap revealed that hPSC-BCs, hPSC-BC-CD144^{+}CD45^{+} contained principally embryonic erythrocytes and to lesser extent definitive erythrocytes.

Undifferentiated hESC (H1) , hESC-derived cell populations (n=3) and EL cell populations (n=4) After normalization, for each cell population, a Tukey's Biweight Robust Mean is calculated from log2 expression values. For each gene analyzed the most highly expressed transcript, is in red, the lowest level expressed transcript is in green. ID, Identifiant Affymetrix; Undiff, undifferentiated.
