# Supplementary material for: Endothelial and hematopoietic hPSCs differentiation via a hematoendothelial progenitor
Source: Stem Cell Res Ther. 2022 Jun 17;13:254. doi: 10.1186/s13287-022-02925-w (PMC9205076; doi:10.1186/s13287-022-02925-w)
Supplement: Supplementary file 10 — Additional file 10. Supplementary table 1. List of antibodies used in flow cytometry and immunofluorescence analysis. [file 13287_2022_2925_MOESM10_ESM.pdf]

Supplementary table 1. List of antibodies used in flow cytometry and immunofluorescence analysis.

Antibodies	Source	Catalogue number
PE Mouse Anti-Human SSEA-4	BD Biosciences	Cat# 560128, RRID : AB_1645533
PE Mouse Anti-Human TRA-1-81	BD Biosciences	Cat# 560161, RRID : AB_1645540
APC Mouse Anti-Human CD34	BD Biosciences	Cat# 555824, RRID : AB_398614
APC Mouse Anti-Human CD41a	BD Biosciences	Cat# 559777, RRID : AB_398671
FITC Mouse Anti-Human CD45	BD Biosciences	Cat# 555482, RRID : AB_395874
FITC Mouse Anti-Human CD43	BD Biosciences	Cat# 555475, RRID : AB_395867
Rabbit Anti-Human Von Willebrand Factor Polyclonal Antibody, Unconjugated	Dako, Agilent	Cat# A0082, RRID : AB_2315602
Donkey Anti-Mouse IgG (H+L) Highly Cross-Adsorbed Secondary Antibody, Alexa Fluor 546	Invitrogen	Cat# A10036, RRID : AB_2534012
Donkey Anti-Mouse IgG (H+L) Highly Cross-Adsorbed Secondary Antibody, Alexa Fluor Plus 488	Invitrogen	Cat# A32766, RRID : AB_2762823
Donkey Anti-Goat IgG (H+L) Cross-Adsorbed Secondary Antibody, Alexa Fluor 568	Invitrogen	Cat# A11057, RRID : AB_2534104
Donkey Anti-Rabbit IgG (H+L) Highly Cross-Adsorbed Secondary Antibody, Alexa Fluor 546	Invitrogen	Cat# A10040, RRID : AB_2534016
CD144 (VE-Cadherin) MicroBeads, human	Miltenyi Biotec	Cat# 130-097-857
APC Mouse Anti-Human CD309 (VEGFR-2)	Miltenyi Biotec	Cat# 130-120-478, RRID : AB_2784122
PE Anti-Human CD143 (ACE) REAfinity™	Miltenyi Biotec	Cat# 130-107-951, RRID : AB_2655145
PE Anti-HumanCD144 (VE-Cadherin) REAfinity™	Miltenyi Biotec	Cat# 130-118-358, RRID : AB_2751492
FITC Mouse Anti-Human CD31	Miltenyi Biotec	Cat# 130-092-654, RRID : AB_871662
PE Anti-Human eNOS, REAfinity™	Miltenyi Biotec	Cat# 130-106-787, RRID : AB_2651629
APC Anti-Human CD54 (ICAM-1), , REAfinity™	Miltenyi Biotec	Cat# 30-103-840, RRID : AB_2658697
Goat Anti-Human VE-Cadherin Polyclonal Antibody, Unconjugated	R&D Systems	Cat# AF938, RRID : AB_355726
Mouse Anti-Human CD31 / pecam-1 Monoclonal Antibody, Unconjugated	R&D Systems	Cat# BBA7, RRID : AB_356960
