# Supplementary material for: Endothelial and hematopoietic hPSCs differentiation via a hematoendothelial progenitor
Source: Stem Cell Res Ther. 2022 Jun 17;13:254. doi: 10.1186/s13287-022-02925-w (PMC9205076; doi:10.1186/s13287-022-02925-w)
Supplement: Supplementary file 11 — Additional file 11. Supplementary table 2. Oligonucleotides used in RTqPCR experiments for hPSC-EBs, hPSC-ECs and hPSC-BCs. [file 13287_2022_2925_MOESM11_ESM.pdf]

Supplementary table 2. Oligonucleotides used in RTqPCR experiments for hPSC-EBs, hPSC-ECs and hPSC-BCs.

	Forward	Reverse
**RUNX1 (a+b+c)**	TCGGCTGAGCTGAGAAATG	GTGATGGTCAGAGTGAAGCTTTT
**RUNX1c**	GTGCATTTTCAGGAGGAAGC	TCGTGGACGTCTCTAGAAGGA
**TBXT**	GCTCACCAATGAGATGATCG	AGACACGTTCACCTTCAGCA
**KDR**	GCTCAAGACAGGAAGACCAAG	GGTGCCACACGCTCTAGG
**ETV2**	GATGCCCCAAAACTAACCAC	GGCTGTTGCCAGTCCAAC
**GATA1**	CCCTGTCCCCAATAGTGCT	CCTGCCCGTTTACTGACAAT
**GATA2**	AAGGCTCGTTCCTGTTCAGA	GGCATTGCACAGGTAGTGG
**NANOG**	CCTATGCCTGTGATTTGT	GTTGTTTGCCTTTGGGAC
**POU5F1**	CGAAAGAGAAAGCGAACCAG	TGAGAAAGGAGACCCAGCAC
**TAL1**	AATCGAGTGAAGAGGAGACCTTC	CTCATTCTTGCTGAGCTTCTTGT
**18S**	GATATGCTCATGTGGTGTTG	AATCTTCTTCAGTCGCTCCA
**ERCC3**	GCTGGTCTTGAAGCACAACA	GGATGCAACGTTTCTGGAGT
**HBB**	GGCAAGGTGAACGTGGATGAA	CAAAGTGATGGGCCAGCACAC
**HBE1**	GGAGCAAGATGAATGTGGAAGAGG	GCCAAAGTGAGTAGCCAGAATAATC
**HBG**	GGGCAAGGTGAATGTGGAAGAT	CGAAATGGATTGCCAAAACGG
**HOXA3**	CACAAAGCAGAAAACCAGCA	ACAGGTAGCGGTTGAAGTGG
