# Supplementary material for: Endothelial and hematopoietic hPSCs differentiation via a hematoendothelial progenitor
Source: Stem Cell Res Ther. 2022 Jun 17;13:254. doi: 10.1186/s13287-022-02925-w (PMC9205076; doi:10.1186/s13287-022-02925-w)
Supplement: Supplementary file 12 — Additional file 12. Supplementary table 3. Oligonucleotides used in RTqPCR experiments in artery and vein differentiation. [file 13287_2022_2925_MOESM12_ESM.pdf]

Supplementary table 3. Oligonucleotides used in RTqPCR experiments in artery and vein differentiation.

	**Source**	**Identifier**
EFNB2	ThermoFisher Scientific	Assay ID: Hs00187950_m1
NR2F2	ThermoFisher Scientific	Assay ID: Hs00819630_m1
EPHB4	ThermoFisher Scientific	Assay ID: Hs00174752_m1
NRP1	ThermoFisher Scientific	Assay ID: Hs00826128_m1
NRP2	ThermoFisher Scientific	Assay ID: Hs00187290_m1
18S	ThermoFisher Scientific	Assay ID: Hs99999901_s1
