# Supplementary material for: Endothelial and hematopoietic hPSCs differentiation via a hematoendothelial progenitor
Source: Stem Cell Res Ther. 2022 Jun 17;13:254. doi: 10.1186/s13287-022-02925-w (PMC9205076; doi:10.1186/s13287-022-02925-w)
Supplement: Supplementary file 13 — Additional file 13. Supplementary table 4. Oligonucleotides used in qPCR for the detection of human DNA. [file 13287_2022_2925_MOESM13_ESM.pdf]

Supplementary table 4. Oligonucleotides used in qPCR for the detection of human DNA.

	**Forward**	**Reverse**
**hCD45**	ACTTTCCCATCTGATCTCTGATTC	CTGGATTTATCATCTGGGTTTGT
**Mouse actine**	GTACCACAGGCATTGTGATG	GCAACATAGCACAGCTTCTC
