# Supplementary material for: Endothelial and hematopoietic hPSCs differentiation via a hematoendothelial progenitor
Source: Stem Cell Res Ther. 2022 Jun 17;13:254. doi: 10.1186/s13287-022-02925-w (PMC9205076; doi:10.1186/s13287-022-02925-w)
Supplement: Supplementary file 14 — Additional file 14. Supplementary table 5. Average percentage ± SD of positive cells for the endothelial and hematopoietic markers analyzed by flow cytometry in hPSC-EBs. Supplementary to Figure 1F. [file 13287_2022_2925_MOESM14_ESM.pdf]

Supplementary table 5. Average percentage ± SD of positive cells for the endothelial and hematopoietic markers analyzed by flow cytometry in hPSC-EBs. Supplementary to Figure 1F.

	A29		SA01		H1	
**Markers**	**Mean %**	**± SD**	**Mean %**	**± SD**	**Mean %**	**± SD**
**CD309**	20,4	12,8	20,1	8,3	27,1	12,4
**CD144**	16,5	5,8	15,4	5,0	17,5	8,0
**CD31**	9,1	5,4	10,8	5,5	11,3	6,9
**CD34**	15,3	7,1	17,4	8,2	15,4	7,9
**CD143**	24,8	14,8	27,0	13,2	36,1	13,2
**CD43**	1,9	3,3	1,8	2,0	2,4	2,8
**CD41**	2,4	6,8	0,2	0,6	0,3	0,8
**CD45**	0,2	0,3	0,2	0,6	0,2	0,6
