# Supplementary material for: Endothelial and hematopoietic hPSCs differentiation via a hematoendothelial progenitor
Source: Stem Cell Res Ther. 2022 Jun 17;13:254. doi: 10.1186/s13287-022-02925-w (PMC9205076; doi:10.1186/s13287-022-02925-w)
Supplement: Supplementary file 15 — Additional file 15. Supplementary table 6. Average percentage ± SD of positive cells for the endothelial and hematopoietic markers analyzed by flow cytometry in hPSC-ECs during consecutive passages. Supplementary to Figure 2D. [file 13287_2022_2925_MOESM15_ESM.pdf]

Supplementary table 6. Average percentage ± SD of positive cells for the endothelial and hematopoietic markers analyzed by flow cytometry in hPSC-ECs during consecutive passages. Supplementary to Figure 2D.

	A29					
	P1		P2		P3	
**Markers**	**Mean %**	**± SD**	**Mean %**	**± SD**	**Mean %**	**± SD**
**CD309**	58,9	24,2	66,6	15,9	65,2	22,8
**CD144**	96,7	4,2	97,8	3,6	88,0	15,6
**CD31**	93,7	6,6	94,6	4,6	87,2	15,3
**CD34**	88,7	11,5	88,1	12,3	91	13,4
**CD143**	54,0	27,7	59,0	25,9	74,8	19,2
**CD43**	0,8	1,0	0,4	0,8	0,5	1,0
**CD41**	2,4	6,8	0,75	1,0	0,0	0,0
**CD45**	0,2	0,3	2,8	4,9	0,0	0,0

	SA01									
	P1		P2		P3		P4		P5	
**Markers**	**Mean %**	**± SD**	**Mean %**	**± SD**	**Mean %**	**± SD**	**Mean %**	**± SD**	**Mean %**	**± SD**
**CD309**	52,6	21,0	55,0	26,4	69	13,9	59,5	0,7	58,3	19,6
**CD144**	93,0	8,6	87,9	20,1	90,6	16,6	97	2,8	98,3	1,2
**CD31**	90,2	9,6	86,6	19,8	88,8	16,3	98,5	0,7	97	1,7
**CD34**	90,0	8,5	88,9	13,5	89,8	6,9	91,5	4,9	87,3	3,1
**CD143**	54,1	27,4	58,9	23,1	66,8	8,3	72	24,0	72,7	15,7
**CD43**	2,7	5,8	1,4	2,5	0	0	0	0	0	0
**CD41**	0,2	0,8	1,3	1,0	1,5	0,1	-	-	-	-
**CD45**	2,3	4,0	0,8	0,2	2,8	0,2	-	-	-	-

	H1									
	P1		P2		P3		P4		P5	
**Markers**	**Mean %**	**± SD**	**Mean %**	**± SD**	**Mean %**	**± SD**	**Mean %**	**± SD**	**Mean %**	**± SD**
**CD309**	57,2	27,2	44,2	22,9	38,8	26,6	33,7	37,1	35,7	26,0
**CD144**	95,8	3,7	95,4	5,4	97,2	2,4	82,7	25,7	94,7	5,9
**CD31**	94,6	4,9	94,0	6,8	93,2	4,6	82,7	24,8	94,3	5,6
**CD34**	87,1	13,3	82,1	22,4	84,5	18,0	74,0	38,2	67,4	29,0
**CD143**	58,0	32,0	68,8	22,2	68,0	19,2	36,3	28,1	79,3	19,4
**CD43**	2,5	4,4	2,1	1,9	2,0	0,9	0,7	1,2	1,3	2,3
**CD41**	0,8	0,1	1,8	0,9	0,9	0,2	-	-	-	-
**CD45**	0,2	0,5	0,2	0,3	0,4	0,4	-	-	-	-
