# Supplementary material for: Endothelial and hematopoietic hPSCs differentiation via a hematoendothelial progenitor
Source: Stem Cell Res Ther. 2022 Jun 17;13:254. doi: 10.1186/s13287-022-02925-w (PMC9205076; doi:10.1186/s13287-022-02925-w)
Supplement: Supplementary file 16 — Additional file 16. Supplementary table 7. Summary of average ± SD values of in vitro endothelial characterization tests: Matrigel tube formation assay, TNFα activation assay, eNOS expression and NO production. Supplementary to Figure 3A-D. [file 13287_2022_2925_MOESM16_ESM.pdf]

Supplementary table 7. Summary of average ± SD values of *in vitro* endothelial characterization tests: Matrigel tube formation assay, TNFα activation assay, eNOS expression and NO production. Supplementary to Figure 3A-D.

	**Matrigel**							
	**A29**		**SA01**		**H1**		**ECFC**	
**Measured parameter**	**Mean**	**± SD**	**Mean**	**± SD**	**Mean**	**± SD**	**Mean**	**± SD**
**N° nodes**	447,3	229,03	390,7	179,2	561,9	132,6	217,5	85,6
**Total segment length**	8399,8	3172,9	8499,7	3495,3	10373,5	1669,6	6857,5	2106,5
**Total branching length**	4729,1	1751,6	4781,5	1506,4	5539,6	948,7	3113	179,6
**N° master segments**	78,92	46,3	72,2	37,1	101,9	26,3	41	19,8
**N° meshes**	29,5	21,6	25,9	15,4	39,7	11,9	14,5	9,2
**N° segments**	148,4	80,5	129,5	62,6	188,5	46,5	73	32,5
**Branching interval**	112,5	33,56	128,0	63,4	113,5	19,3	161,7	33,8
**N° master segments**	78,9	46,3	72,2	37,1	101,9	26,3	41	19,8

	**% ICAM^{High} cells**							
	**A29**		**SA01**		**H1**		**ECFC**	
	**Mean**	**± SD**	**Mean**	**± SD**	**Mean**	**± SD**	**Mean**	**± SD**
**-TNFα**	2,7	4,6	6,8	6,7	5,0	1,6	13,3	8,4
**+ TNFα**	93,3	4,9	94,5	5,7	90,3	2,5	93,5	7,1

**% eNOS^{+} cells**							
**A29**		**SA01**		**H1**		**ECFC**	
**Mean**	**± SD**	**Mean**	**± SD**	**Mean**	**± SD**	**Mean**	**± SD**
72,3	16,4	86,0	17,0	84,5	24,5	74,3	18,9

	**% NO producer cells**							
	**A29**		**SA01**		**H1**		**ECFC**	
	**Mean %**	**± SD**	**Mean %**	**± SD**	**Mean %**	**± SD**	**Mean %**	**± SD**
-DAF-FM	0	0	0	0	0	0	0	0
+DAF-FM	83,3	12,6	91,7	11,8	90,3	5,0	88,0	9,0
+DAF-FM +LPS	93,3	5,7	97,3	3,8	95,3	4,7	94,5	8,3
+DAF-FM +SNAP	91,7	11,0	95,7	5,8	95,3	3,5	95,4	4,5
