# Supplementary material for: Endothelial and hematopoietic hPSCs differentiation via a hematoendothelial progenitor
Source: Stem Cell Res Ther. 2022 Jun 17;13:254. doi: 10.1186/s13287-022-02925-w (PMC9205076; doi:10.1186/s13287-022-02925-w)
Supplement: Supplementary file 17 — Additional file 17. Supplementary table 8. Average percentage of positive cells for the endothelial and hematopoietic markers analyzed by flow cytometry in hPSC-BCs. Supplementary to Figure 4B-C. [file 13287_2022_2925_MOESM17_ESM.pdf]

Supplementary table 8. Average percentage of positive cells for the endothelial and hematopoietic markers analyzed by flow cytometry in hPSC-BCs. Supplementary to Figure 4B-C.

	A29		SA01		H1	
**Markers**	**Mean %**	**± SD**	**Mean %**	**± SD**	**Mean %**	**± SD**
**CD309**	14,5	8,3	7,25	5,7	7,0	1,0
**CD144**	20,0	8,0	8,7	3,5	29,3	5,0
**CD31**	63,8	8,3	32,75	12,3	79	6,9
**CD34**	48,6	11,3	22,1	7,9	74,3	12,7
**CD143**	37,5	14,4	23,5	10,1	51,7	6,0
**CD43**	91,1	9,6	90,25	7,5	94,0	2,6
**CD41**	33,8	15,45	38,5	18,3	64,0	17,67
**CD45**	61,6	17,1	33,6	11,0	56,3	11,0
**$CD144^+$ $CD31^+$**	18,7	12,9	6,2	3,9	28,0	7,2
**$CD144^+$ $CD309^+$**	9,2	8,2	2,67	2,0	4,7	1,2
**$CD34^+$ $CD143^+$**	21,4	12,1	11,4	7,8	46,3	2,5
**$CD34^+$ $CD43^+$**	33,6	18,6	21,8	11,0	71,7	11,2
**$CD143^+$ $CD43^+$**	27,5	14,9	24,2	12,1	47,7	6,6
**$CD41^+$ $CD45^+$**	18,6	6,6	13,3	3,0	37,0	27,3
**$CD144^+$ $CD45^+$**	18,3	8,0	5,6	1,3	25,0	9,6
