# Supplementary material for: Endothelial and hematopoietic hPSCs differentiation via a hematoendothelial progenitor
Source: Stem Cell Res Ther. 2022 Jun 17;13:254. doi: 10.1186/s13287-022-02925-w (PMC9205076; doi:10.1186/s13287-022-02925-w)
Supplement: Supplementary file 18 — Additional file 18. Supplementary table 9. Contains a list of significantly hematopoietic and endothelial Gene Ontology Biological Process enrichment in hPSC-EB-CD144+, hPSC-BCs, hPSC-BC-CD144+CD45+ and hPSC-ECs vs undifferentiated hESC. Enrichment analysis was performed using Gene Set Enrichment Analysis software v4.1.0 and probing the c5.go.bp.v7.4 collection of the Molecular Signatures Database (MSigDB ). Number of genes involved in the gene set, Enrichment score (ES), normalized enrichment score (NES) and nominal P-value are given in the supplementary Table. All selected Gene Ontology Biological Process enrichment show a P value ≤ 0.05. [file 13287_2022_2925_MOESM18_ESM.pdf]

Supplementary table 9. Contains a list of significantly hematopoietic and endothelial Gene Ontology Biological Process enrichment in hPSC-EB-CD144$^+$, hPSC-BCs, hPSC-BC-CD144$^+$CD45$^+$ and hPSC-ECs vs undifferentiated hESC. Enrichment analysis was performed using Gene Set Enrichment Analysis software v4.1.0 and probing the c5.go.bp.v7.4 collection of the Molecular Signatures Database (MSigDB). Number of genes involved in the gene set, Enrichment score (ES), normalized enrichment score (NES) and nominal P-value are given in the supplementary Table. All selected Gene Ontology Biological Process enrichment show a P value ≤ 0.05.

Hemopoiesis | Endothelium | Erythrocyte | Myeloid | Megakaryocyte

Leukocyte | Lymphoid cell | Immune System

hPSC-EB-CD144$^+$ *vs* hESCs

NAME	SIZ	ES	NES	NOM p-va
GOBP_EMBRYONIC_HEMOPOIESIS	23	0,5254805	1,6547778	0,018518519
GOBP_HEMATOPOIETIC_PROGENITOR_CELL_DIFFERENTIATION	158	0,5792662	2,699441	0
GOBP_HEMATOPOIETIC_STEM_CELL_DIFFERENTIATION	88	0,6427582	2,706844	0
GOBP_REGULATION_OF_HEMATOPOIETIC_PROGENITOR_CELL_DIFFERENTIATION	89	0,6784608	2,8777285	0
GOBP_REGULATION_OF_HEMATOPOIETIC_STEM_CELL_DIFFERENTIATION	74	0,68638617	2,8659632	0
GOBP_REGULATION_OF_HEMOPOIESIS	375	0,306668	1,5586056	0
GOBP_ENDOTHELIUM_DEVELOPMENT	131	0,29242912	1,3090827	0,04692082
GOBP_POSITIVE_REGULATION_OF_VASCULAR_ENDOTHELIAL_GROWTH_FACTOR_PRODUCTION	28	0,4562116	1,5213748	0,045801528
GOBP_VASCULAR_ENDOTHELIAL_GROWTH_FACTOR_RECEPTOR_SIGNALING_PATHWAY	91	0,42548275	1,7780535	0
GOBP_ERYTHROCYTE_DEVELOPMENT	31	0,48191306	1,6375896	0,014209592
GOBP_ERYTHROCYTE_HOMEOSTASIS	117	0,43918675	1,9469122	0
GOBP_POSITIVE_REGULATION_OF_ERYTHROCYTE_DIFFERENTIATION	36	0,613802	2,1968856	0
GOBP_MEGAKARYOCYTE_DEVELOPMENT	132	0,4145793	1,8396958	0
GOBP_MEGAKARYOCYTE_DIFFERENTIATION	15	0,6625636	1,9064964	0,001930502
GOBP_REGULATION_OF_MEGAKARYOCYTE_DIFFERENTIATION	69	0,5379506	2,1668715	0
GOBP_MYELOID_CELL_DEVELOPMENT	53	0,49123913	1,8918791	0,003597122
GOBP_MYELOID_CELL_DIFFERENTIATION	64	0,46057147	1,8202299	0,001706485
GOBP_MYELOID_CELL_HOMEOSTASIS	379	0,36891356	1,8919784	0
GOBP_MYELOID_LEUKOCYTE_CYTOKINE_PRODUCTION	141	0,44520748	2,012909	0
GOBP_REGULATION_OF_MYELOID_CELL_DIFFERENTIATION	98	0,60889834	2,6435843	0
GOBP_POSITIVE_REGULATION_OF_MYELOID_LEUKOCYTE_CYTOKINE_PRODUCTION_INVOLVED_IN_IMMUNE_RESPONSE	32	0,6895977	2,3926609	0
GOBP_LEUKOCYTE_APOPTOTIC_PROCESS	16	0,62675905	1,8103799	0,003898636
GOBP_LEUKOCYTE_HOMEOSTASIS	85	0,39507228	1,6386065	0

hPSC-BCs *vs* hESCs

NAME	SIZE	ES	NES	NOM p-val
GOBP_REGULATION_OF_HEMATOPOIETIC_STEM_CELL_DIFFERENTIATION	74	0,7191193	2,8600254	0
GOBP_REGULATION_OF_HEMATOPOIETIC_PROGENITOR_CELL_DIFFERENTIATION	89	0,68365055	2,8202999	0
GOBP_HEMATOPOIETIC_STEM_CELL_DIFFERENTIATION	88	0,6842167	2,81331	0
GOBP_HEMATOPOIETIC_PROGENITOR_CELL_DIFFERENTIATION	158	0,5823979	2,5657387	0
GOBP_REGULATION_OF_HEMOPOIESIS	375	0,3358644	1,6100389	0
GOBP_EMBRYONIC_HEMOPOIESIS	23	0,5690219	1,7966584	0,006557377
GOBP_VASCULAR_ENDOTHELIAL_GROWTH_FACTOR_RECEPTOR_SIGNALING_PATHWAY	91	0,39152935	1,5896512	0,010810811
GOBP_ERYTHROCYTE_HOMEOSTASIS	117	0,5105271	2,1430273	0
GOBP_POSITIVE_REGULATION_OF_ERYTHROCYTE_DIFFERENTIATION	31	0,5885723	1,973608	0
GOBP_ERYTHROCYTE_DEVELOPMENT	31	0,5732315	1,912028	0
GOBP_REGULATION_OF_ERYTHROCYTE_DIFFERENTIATION	46	0,5076777	1,8362316	0,002976191
GOBP_MEGAKARYOCYTE_DIFFERENTIATION	69	0,61334205	2,4054708	0
GOBP_MEGAKARYOCYTE_DEVELOPMENT	15	0,67227316	1,8627614	0
GOBP_REGULATION_OF_MEGAKARYOCYTE_DIFFERENTIATION	53	0,58970696	2,2039273	0
GOBP_MYELOID_CELL_HOMEOSTASIS	141	0,5058612	2,2065067	0
GOBP_REGULATION_OF_MYELOID_CELL_DIFFERENTIATION	222	0,3976876	1,8198955	0
GOBP_POSITIVE_REGULATION_OF_MYELOID_CELL_DIFFERENTIATION	99	0,41029593	1,7125163	0
GOBP_MYELOID_CELL_DIFFERENTIATION	379	0,41333562	1,9771869	0
GOBP_MYELOID_CELL_DEVELOPMENT	64	0,49684045	1,9212459	0
GOBP_GRANULOCYTE_DIFFERENTIATION	33	0,44910172	1,5052586	0,047328245
GOBP_LYMPHOCYTE_ACTIVATION_INVOLVED_IN_IMMUNE_RESPONSE	184	0,31042713	1,4059935	0,01097561
GOBP_LYMPHOCYTE_HOMEOSTASIS	57	0,41419455	1,5536903	0,022222223
GOBP_T_CELL_RECEPTOR_SIGNALING_PATHWAY	194	0,48355088	2,1904423	0
GOBP_B_CELL_ACTIVATION_INVOLVED_IN_IMMUNE_RESPONSE	74	0,43751642	1,7529659	0,001371742
GOBP_B_CELL_HOMEOSTASIS	28	0,56945467	1,8467761	0,001631321
GOBP_POSITIVE_REGULATION_OF_B_CELL_DIFFERENTIATION	15	0,6263547	1,7377572	0,012367492
GOBP_B_CELL_ACTIVATION	284	0,2776108	1,3191406	0,022446688
GOBP_B_CELL_DIFFERENTIATION	136	0,32358435	1,4060766	0,03448276
GOBP_LEUKOCYTE_HOMEOSTASIS	79	0,41109908	1,6498717	0,004126547
GOBP_ACTIVATION_OF_INNATE_IMMUNE_RESPONSE	143	0,5893772	2,578446	0
GOBP_REGULATION_OF_INNATE_IMMUNE_RESPONSE	306	0,50110555	2,3594534	0
GOBP_INNATE_IMMUNE_RESPONSE_ACTIVATING_SIGNAL_TRANSDUCTION	118	0,54763496	2,324258	0
GOBP_SOMATIC_DIVERSIFICATION_OF_IMMUNE_RECEPTORS	69	0,55995256	2,171281	0
GOBP_SOMATIC_DIVERSIFICATION_OF_IMMUNE_RECEPTORS_VIA_SOMATIC_MUTATION	16	0,67423946	1,9543653	0
GOBP_IMMUNE_RESPONSE_REGULATING_SIGNALING_PATHWAY	40	0,64925694	2,3134427	0
GOBP_IMMUNOGLOBULIN_PRODUCTION_INVOLVED_IN_IMMUNOGLOBULIN_MEDIATED_IMMUNE_RESPONSE	49	0,4908481	1,775441	0,002967359

hPSC-BC-CD144^{+}CD45^{+} *vs* hESCs

NAME	SIZE	ES	NES	NOM p-val
GOBP_REGULATION_OF_HEMOPOIESIS	375	0,426282	2,0953286	0
GOBP_POSITIVE_REGULATION_OF_HEMOPOIESIS	151	0,4192429	1,8797725	0
GOBP_EMBRYONIC_HEMOPOIESIS	23	0,6203057	1,9516518	0,001669449
GOBP_REGULATION_OF_HEMATOPOIETIC_STEM_CELL_DIFFERENTIATION	74	0,6955431	2,7666595	0
GOBP_HEMATOPOIETIC_STEM_CELL_DIFFERENTIATION	88	0,6591906	2,763276	0
GOBP_REGULATION_OF_HEMATOPOIETIC_PROGENITOR_CELL_DIFFERENTIATION	89	0,64723206	2,7288601	0
GOBP_HEMATOPOIETIC_PROGENITOR_CELL_DIFFERENTIATION	158	0,56177336	2,5746481	0
GOBP_VASCULAR_ENDOTHELIAL_GROWTH_FACTOR_RECEPTOR_SIGNALING_PATHWAY	91	0,4305598	1,8228532	0
GOBP_POSITIVE_REGULATION_OF_ERYTHROCYTE_DIFFERENTIATION	31	0,66393554	2,2001503	0
GOBP_ERYTHROCYTE_HOMEOSTASIS	117	0,49052125	2,1509287	0
GOBP_REGULATION_OF_ERYTHROCYTE_DIFFERENTIATION	46	0,58469844	2,1503425	0
GOBP_ERYTHROCYTE_DEVELOPMENT	69	0,7066413	2,819529	0
GOBP_MEGAKARYOCYTE_DIFFERENTIATION	53	0,6801214	2,5563688	0
GOBP_REGULATION_OF_MEGAKARYOCYTE_DIFFERENTIATION	15	0,78460735	2,2090528	0
GOBP_MEGAKARYOCYTE_DEVELOPMENT	31	0,5321034	1,7688103	0,008361204
GOBP_MYELOID_CELL_DIFFERENTIATION	379	0,48637992	2,380282	0
GOBP_REGULATION_OF_MYELOID_CELL_DIFFERENTIATION	222	0,4862088	2,2947261	0
GOBP_MYELOID_CELL_HOMEOSTASIS	141	0,5000715	2,2309575	0
GOBP_MYELOID_CELL_DEVELOPMENT	64	0,54712766	2,135457	0
GOBP_POSITIVE_REGULATION_OF_MYELOID_CELL_DIFFERENTIATION	99	0,481474	2,035618	0
GOBP_MYELOID_LEUKOCYTE_DIFFERENTIATION	199	0,40220228	1,8561171	0
GOBP_REGULATION_OF_MYELOID_LEUKOCYTE_MEDIATED_IMMUNITY	52	0,48214766	1,8085638	0
GOBP_REGULATION_OF_MYELOID_LEUKOCYTE_DIFFERENTIATION	114	0,3664183	1,5820631	0,002762431
GOBP_MYELOID_LEUKOCYTE_MIGRATION	204	0,3225073	1,5056573	0,00375
GOBP_POSITIVE_REGULATION_OF_MYELOID_LEUKOCYTE_DIFFERENTIATION	57	0,4424027	1,6679332	0,005988024
GOBP_NEGATIVE_REGULATION_OF_MYELOID_CELL_DIFFERENTIATION	76	0,38858154	1,5694475	0,013392857
GOBP_MYELOID_DENDRITIC_CELL_ACTIVATION	29	0,49277386	1,6474639	0,013468013
GOBP_MYELOID_LEUKOCYTE_CYTOKINE_PRODUCTION	26	0,49858448	1,6296837	0,022413794
GOBP_NEUTROPHIL_MIGRATION	116	0,33313757	1,434634	0,021917809
GOBP_NEUTROPHIL_CHEMOTAXIS	97	0,3712755	1,5648873	0,008403362
GOBP_MAST_CELL_ACTIVATION_INVOLVED_IN_IMMUNE_RESPONSE	46	0,5489144	2,0124369	0
GOBP_MAST_CELL_ACTIVATION	58	0,5130783	1,9790058	0
GOBP_REGULATION_OF_MAST_CELL_ACTIVATION_INVOLVED_IN_IMMUNE_RESPONSE	30	0,54469746	1,8339247	0,001663894
GOBP_REGULATION_OF_MAST_CELL_ACTIVATION	39	0,48138306	1,6986775	0,006430868
GOBP_LYMPHOCYTE_ACTIVATION_INVOLVED_IN_IMMUNE_RESPONSE	184	0,39948472	1,8357497	0
GOBP_REGULATION_OF_LYMPHOCYTE_ACTIVATION	456	0,28381386	1,4244148	0
GOBP_LYMPHOCYTE_HOMEOSTASIS	57	0,479855	1,8336222	0,001503759
GOBP_REGULATION_OF_LYMPHOCYTE_DIFFERENTIATION	173	0,3470733	1,582589	0,00256082
GOBP_POSITIVE_REGULATION_OF_LYMPHOCYTE_DIFFERENTIATION	100	0,40011662	1,687281	0,002801121
GOBP_REGULATION_OF_LYMPHOCYTE_MIGRATION	59	0,40198606	1,5484954	0,016541352
GOBP_POSITIVE_REGULATION_OF_LYMPHOCYTE_MIGRATION	36	0,42934465	1,5012125	0,0455312
GOBP_T_CELL_LINEAGE_COMMITMENT	27	0,5478518	1,7813352	0,003327787
GOBP_T_CELL_RECEPTOR_SIGNALING_PATHWAY	194	0,5226132	2,4220293	0
GOBP_T_CELL_ACTIVATION	460	0,3459406	1,7402446	0
GOBP_T_CELL_DIFFERENTIATION	244	0,34494835	1,6289542	0
GOBP_REGULATION_OF_T_CELL_ACTIVATION	314	0,31509876	1,5238477	0
GOBP_REGULATION_OF_T_CELL_DIFFERENTIATION	144	0,3391448	1,5137659	0,006666667
GOBP_T_CELL_MIGRATION	62	0,4233886	1,6559789	0,002989537
GOBP_POSITIVE_REGULATION_OF_T_CELL_MIGRATION	29	0,46365365	1,5276139	0,03125
GOBP_POSITIVE_REGULATION_OF_ALPHA_BETA_T_CELL_ACTIVATION	65	0,44352597	1,7342634	0,001545595
GOBP_POSITIVE_REGULATION_OF_CD4_POSITIVE_ALPHA_BETA_T_CELL_ACTIVATION	38	0,4627821	1,6306753	0,001666667
GOBP_CD4_POSITIVE_ALPHA_BETA_T_CELL_ACTIVATION	97	0,36605084	1,559438	0,008534851
GOBP_ALPHA_BETA_T_CELL_ACTIVATION	146	0,34790444	1,5353842	0,006613757
GOBP_B_CELL_HOMEOSTASIS	28	0,6488756	2,1022246	0
GOBP_POSITIVE_REGULATION_OF_B_CELL_DIFFERENTIATION	15	0,7089446	1,9900485	0
GOBP_B_CELL_ACTIVATION_INVOLVED_IN_IMMUNE_RESPONSE	74	0,48532432	1,9439756	0
GOBP_B_CELL_DIFFERENTIATION	136	0,39240506	1,7525575	0
GOBP_B_CELL_ACTIVATION	284	0,3317585	1,5925012	0
GOBP_B_CELL_APOPTOTIC_PROCESS	25	0,5495275	1,7621042	0,003401361
GOBP_LEUKOCYTE_HOMEOSTASIS	79	0,46703365	1,9253492	0
GOBP_REGULATION_OF_LEUKOCYTE_DIFFERENTIATION	272	0,3562978	1,7200013	0
GOBP_LEUKOCYTE_PROLIFERATION	304	0,3097039	1,4928325	0,001187649
GOBP_POSITIVE_REGULATION_OF_LEUKOCYTE_CELL_CELL_ADHESION	225	0,3433133	1,6111315	0,002460025
GOBP_LEUKOCYTE_CHEMOTAXIS	213	0,32772407	1,5358342	0,002557545
GOBP_LEUKOCYTE_MIGRATION	471	0,27528164	1,381385	0,003289474
GOBP_LEUKOCYTE_CELL_CELL_ADHESION	351	0,29115477	1,427517	0,003516999
GOBP_REGULATION_OF_LEUKOCYTE_DEGRANULATION	43	0,45346266	1,6336738	0,009584664
GOBP_POSITIVE_REGULATION_OF_LEUKOCYTE_PROLIFERATION	144	0,34332934	1,5190574	0,013227513
GOBP_REGULATION_OF_LEUKOCYTE_MEDIATED_IMMUNITY	199	0,31099126	1,4347152	0,015132409
GOBP_LEUKOCYTE_MEDIATED_CYTOTOXICITY	107	0,33658144	1,4450555	0,022130014
GOBP_POSITIVE_REGULATION_OF_LEUKOCYTE_MIGRATION	129	0,3248139	1,4274356	0,023004059
GOBP_REGULATION_OF_LEUKOCYTE_PROLIFERATION	233	0,28036788	1,3166403	0,024600247
GOBP_LEUKOCYTE_APOPTOTIC_PROCESS	101	0,33341715	1,4294116	0,026988637
GOBP_REGULATION_OF_LEUKOCYTE_CHEMOTAXIS	114	0,3255346	1,3989065	0,029810298
GOBP_REGULATION_OF_LEUKOCYTE_MIGRATION	196	0,28755593	1,3346994	0,03379224
GOBP_ACTIVATION_OF_INNATE_IMMUNE_RESPONSE	143	0,58433217	2,6024532	0
GOBP_REGULATION_OF_INNATE_IMMUNE_RESPONSE	306	0,52651477	2,540307	0
GOBP_INNATE_IMMUNE_RESPONSE_ACTIVATING_SIGNAL_TRANSDUCTION	118	0,56147665	2,4240775	0
GOBP_SOMATIC_DIVERSIFICATION_OF_IMMUNE_RECEPTORS	69	0,5769365	2,2766454	0
GOBP_SOMATIC_DIVERSIFICATION_OF_IMMUNE_RECEPTORS_VIA_SOMATIC_MUTATION	16	0,66732657	1,9309615	0,001782531
GOBP_IMMUNE_RESPONSE_REGULATING_SIGNALING_PATHWAY	461	0,3780738	1,8976215	0
GOBP_POSITIVE_REGULATION_OF_IMMUNE_EFFECTOR_PROCESS	210	0,3234279	1,5048718	0,001283697
GOBP_REGULATION_OF_IMMUNE_EFFECTOR_PROCESS	430	0,28105462	1,4001334	0,002217295
GOBP_NEGATIVE_REGULATION_OF_INNATE_IMMUNE_RESPONSE	64	0,44949266	1,7449913	0,004545454
GOBP_NEGATIVE_REGULATION_OF_IMMUNE_SYSTEM_PROCESS	382	0,27598736	1,3722013	0,008055236
GOBP_NEGATIVE_REGULATION_OF_IMMUNE_RESPONSE	144	0,3224637	1,4255928	0,022849463
GOBP_NEGATIVE_REGULATION_OF_IMMUNE_EFFECTOR_PROCESS	123	0,33413303	1,4472044	0,028806584
GOBP_CYTOKINE_PRODUCTION_INVOLVED_IN_IMMUNE_RESPONSE	92	0,33743083	1,4072566	0,049157303
GOBP_REGULATION_OF_PRODUCTION_OF_MOLECULAR_MEDIATOR_OF_IMMUNE_RESPONSE	140	0,2978033	1,3171536	0,049932525

hPSC-ECs *vs* hESCs

NAME	SIZE	ES	NES	NOM p-val
GOBP_REGULATION_OF_HEMATOPOIETIC_PROGENITOR_CELL_DIFFERENTIATION	89	0,6532313	2,6609511	0
GOBP_REGULATION_OF_HEMATOPOIETIC_STEM_CELL_DIFFERENTIATION	74	0,6589702	2,6311827	0
GOBP_HEMATOPOIETIC_STEM_CELL_DIFFERENTIATION	88	0,6268067	2,54444	0
GOBP_HEMATOPOIETIC_PROGENITOR_CELL_DIFFERENTIATION	158	0,55502385	2,4371755	0
GOBP_REGULATION_OF_HEMOPOIESIS	375	0,3136925	1,5171429	0
GOBP_LYMPHOID_PROGENITOR_CELL_DIFFERENTIATION	21	0,51344377	1,5638828	0,029360967
GOBP_POSITIVE_REGULATION_OF_HEMOPOIESIS	151	0,30588982	1,3495471	0,03164557
GOBP_VASCULAR_ENDOTHELIAL_GROWTH_FACTOR_RECEPTOR_SIGNALING_PATHWAY	91	0,48557162	1,9905674	0
GOBP_RETINA_VASCULATURE_DEVELOPMENT_IN_CAMERA_TYPE_EYE	18	0,66682136	1,9577587	0
GOBP_ENDOTHELIUM_DEVELOPMENT	131	0,4232409	1,8419935	0
GOBP_ENDOTHELIAL_CELL_DEVELOPMENT	62	0,4512575	1,7452127	0
GOBP_ENDOTHELIAL_CELL_MIGRATION	257	0,33533987	1,579009	0,001190476
GOBP_VASCULOGENESIS	78	0,43494567	1,7377623	0,001422475
GOBP_POSITIVE_REGULATION_OF_ENDOTHELIAL_CELL_MIGRATION	123	0,3684358	1,5836663	0,002680965
GOBP_ESTABLISHMENT_OF_ENDOTHELIAL_BARRIER	45	0,46837935	1,7151308	0,007788162
GOBP_ENDOTHELIAL_CELL_PROLIFERATION	176	0,31284937	1,4240185	0,008706467
GOBP_REGULATION_OF_ENDOTHELIAL_CELL_MIGRATION	213	0,30620354	1,4211493	0,015568863
GOBP_REGULATION_OF_VASCULOGENESIS	16	0,5966438	1,6846743	0,01584507
GOBP_ENDOTHELIAL_CELL_APOPTOTIC_PROCESS	61	0,3807299	1,4622917	0,029007634
GOBP_POSITIVE_REGULATION_OF_ENDOTHELIAL_CELL_APOPTOTIC_PROCESS	20	0,5114112	1,575634	0,030195381
GOBP_REGULATION_OF_VASCULAR_PERMEABILITY	42	0,4250209	1,5036095	0,035313003
GOBP_POSITIVE_REGULATION_OF_ENDOTHELIAL_CELL_PROLIFERATION	101	0,33509216	1,3964865	0,04027778
GOBP_BRANCHING_INVOLVED_IN_BLOOD_VESSEL_MORPHOGENESIS	17	0,55149174	1,6147072	0,021276595
GOBP_LYMPHANGIOGENESIS	29	0,48502138	1,5735108	0,02685624
GOBP_ANGIOGENESIS_INVOLVED_IN_WOUND_HEALING	17	0,51745945	1,5328506	0,041516244
GOBP_LABYRINTHINE_LAYER_BLOOD_VESSEL_DEVELOPMENT	430	0,69246167	3,3931408	0
GOBP_ERYTHROCYTE_HOMEOSTASIS	117	0,45743918	1,977097	0
GOBP_POSITIVE_REGULATION_OF_ERYTHROCYTE_DIFFERENTIATION	31	0,48283735	1,6102372	0,021103896
GOBP_REGULATION_OF_ERYTHROCYTE_DIFFERENTIATION	46	0,43107173	1,5573173	0,023584906
GOBP_MEGAKARYOCYTE_DIFFERENTIATION	69	0,42282152	1,6451592	0,01010101
GOBP_MYELOID_CELL_DIFFERENTIATION	379	0,3868108	1,8735901	0
GOBP_REGULATION_OF_MYELOID_CELL_DIFFERENTIATION	222	0,34771094	1,6114055	0
GOBP_MYELOID_CELL_DEVELOPMENT	64	0,4100764	1,6102357	0,006144393
GOBP_MYELOID_CELL_HOMEOSTASIS	141	0,4621684	2,0481856	0
GOBP_MYELOID_LEUKOCYTE_DIFFERENTIATION	199	0,30268943	1,3817177	0,015719468
GOBP_POSITIVE_REGULATION_OF_MYELOID_CELL_DIFFERENTIATION	99	0,34679043	1,4305787	0,02739726
GOBP_NEGATIVE_REGULATION_OF_MYELOID_CELL_DIFFERENTIATION	76	0,36198428	1,4569572	0,037956204
GOBP_MYELOID_LEUKOCYTE_CYTOKINE_PRODUCTION	26	0,48894176	1,5817249	0,028911564
GOBP_LEUKOCYTE_HOMEOSTASIS	25	0,51463217	1,6265696	0,011093502
