# Supplementary material for: Endothelial and hematopoietic hPSCs differentiation via a hematoendothelial progenitor
Source: Stem Cell Res Ther. 2022 Jun 17;13:254. doi: 10.1186/s13287-022-02925-w (PMC9205076; doi:10.1186/s13287-022-02925-w)
Supplement: Supplementary file 19 — Additional file 19. Supplementary table 10. Contains list of significantly hematopoietic and endothelial Gene Ontology Biological Process enrichment in hPSC-EB-CD144+, hPSC-BCs, hPSC-BC-CD144+CD45+ and hPSC-ECs compare to EL cell populations. Enrichment analysis was performed using Gene Set Enrichment Analysis software v4.1.0 and probing the c5.go.bp.v7.4 collection of the Molecular Signatures Database (MSigDB). Number of genes involved in the gene set, Enrichment score (ES), normalized enrichment score (NES) and nominal P-value are given in the supplementary Table 6. All selected Gene Ontology Biological Process enrichment show a P value ≤ 0.05. [file 13287_2022_2925_MOESM19_ESM.pdf]

Supplementary table 10. Contains list of significantly hematopoietic and endothelial Gene Ontology Biological Process enrichment in hPSC-EB-CD144^{+}, hPSC-BCs, hPSC-BC-CD144^{+}CD45^{+} and hPSC-ECs compare to EL cell populations. Enrichment analysis was performed using Gene Set Enrichment Analysis software v4.1.0 and probing the c5.go.bp.v7.4 collection of the Molecular Signatures Database (MSigDB). Number of genes involved in the gene set, Enrichment score (ES), normalized enrichment score (NES) and nominal P-value are given in the supplementary Table 6. All selected Gene Ontology Biological Process enrichment show a P value ≤ 0.05.

Hemopoiesis | Endothelium | Erythrocyte | Myeloid | Megakaryocyte

Leukocyte | Lymphoid cell | Immune System | Platelet

hPSC-EB-CD144^{+} *vs* EL-pre-HSCs

NAME	SIZE	ES	NES	NOM p-val
GOBP_RESPONSE_TO_PLATELET_DERIVED_GROWTH_FACTOR	18	0,71	1,98	0,00E+00
GOBP_PLATELET_DERIVED_GROWTH_FACTOR_RECEPTOR_SIGNALING_PATHWAY	56	0,48	1,72	0,00E+00
GOBP_REGULATION_OF_HEMATOPOIETIC_PROGENITOR_CELL_DIFFERENTIATION	89	0,55	2,12	0,00E+00
GOBP_HEMATOPOIETIC_STEM_CELL_DIFFERENTIATION	88	0,54	2,09	0,00E+00
GOBP_REGULATION_OF_HEMATOPOIETIC_STEM_CELL_DIFFERENTIATION	74	0,56	2,07	0,00E+00
GOBP_HEMATOPOIETIC_PROGENITOR_CELL_DIFFERENTIATION	158	0,44	1,85	0,00E+00
GOBP_LYMPHANGIOGENESIS	17	0,84	2,31	0,00E+00
GOBP_VASCULAR_ENDOTHELIAL_GROWTH_FACTOR_SIGNALING_PATHWAY	45	0,54	1,83	0,00E+00
GOBP_ENDOTHELIAL_CELL_MIGRATION	257	0,40	1,78	0,00E+00
GOBP_CELLULAR_RESPONSE_TO_VASCULAR_ENDOTHELIAL_GROWTH_FACTOR_STIMULUS	68	0,48	1,76	0,00E+00
GOBP_SPROUTING_ANGIOGENESIS	164	0,40	1,68	0,00E+00
GOBP_ENDOTHELIAL_CELL_PROLIFERATION	176	0,40	1,68	0,00E+00
GOBP_REGULATION_OF_ENDOTHELIAL_CELL_MIGRATION	213	0,37	1,58	1,39E-03
GOBP_POSITIVE_REGULATION_OF_ENDOTHELIAL_CELL_PROLIFERATION	101	0,43	1,70	1,47E-03
GOBP_BLOOD_VESSEL_ENDOTHELIAL_CELL_MIGRATION	161	0,36	1,49	1,49E-03
GOBP_VASCULAR_ENDOTHELIAL_GROWTH_FACTOR_RECEPTOR_SIGNALING_PATHWAY	91	0,43	1,67	1,52E-03
GOBP_ENDOTHELIAL_CELL_DEVELOPMENT	62	0,46	1,67	1,58E-03
GOBP_POSITIVE_REGULATION_OF_ENDOTHELIAL_CELL_MIGRATION	123	0,36	1,45	1,28E-02
GOBP_REGULATION_OF_CELLULAR_RESPONSE_TO_VASCULAR_ENDOTHELIAL_GROWTH_FACTOR_STIMULUS	26	0,54	1,63	2,15E-02
GOBP_REGULATION_OF_SPROUTING_ANGIOGENESIS	51	0,42	1,45	2,57E-02
GOBP_CELL_MIGRATION_INVOLVED_IN_SPROUTING_ANGIOGENESIS	84	0,36	1,39	4,01E-02
GOBP_ACTIVATION_OF_INNATE_IMMUNE_RESPONSE	143	0,32	1,31	4,55E-02

hPSC-EB-CD144^{+} *vs* EL-ECs

NAME	SIZE	ES	NES	NOM p-val
GOBP_REGULATION_OF_PLATELET_DERIVED_GROWTH_FACTOR_RECEPTOR_SIGNALING_PATHWAY	23	0,54	1,61	1,27E-02
GOBP_PLATELET_MORPHOGENESIS	20	0,53	1,52	3,31E-02
GOBP_HEMATOPOIETIC_PROGENITOR_CELL_DIFFERENTIATION	158	0,46	2,01	0,00E+00
GOBP_HEMATOPOIETIC_STEM_CELL_DIFFERENTIATION	88	0,48	1,90	0,00E+00
GOBP_REGULATION_OF_HEMATOPOIETIC_PROGENITOR_CELL_DIFFERENTIATION	89	0,47	1,89	0,00E+00
GOBP_REGULATION_OF_HEMATOPOIETIC_STEM_CELL_DIFFERENTIATION	74	0,46	1,83	0,00E+00
GOBP_IMMUNOGLOBULIN_PRODUCTION_INVOLVED_IN_IMMUNOGLOBULIN_MEDIATED_IMMUNE_RESPONSE	49	0,40	1,43	3,25E-02

hPSC-BCs *vs* EL-HSCs/HPs

NAME	SIZE	ES	NES	NOM p-val
GOBP_REGULATION_OF_PLATELET_ACTIVATION	34	0,74	2,38	0,00E+00
GOBP_PLATELET_ACTIVATION	156	0,53	2,24	0,00E+00
GOBP_PLATELET_DEGRANULATION	123	0,52	2,13	0,00E+00
GOBP_REGULATION_OF_PLATELET_AGGREGATION	19	0,71	2,01	0,00E+00
GOBP_PLATELET_AGGREGATION	62	0,53	1,90	0,00E+00
GOBP_NEGATIVE_REGULATION_OF_PLATELET_ACTIVATION	17	0,74	1,96	2,12E-03
GOBP_REGULATION_OF_PLATELET_DERIVED_GROWTH_FACTOR_RECEPTOR_SIGNALING_PATHWAY	23	0,57	1,64	6,20E-03
GOBP_RESPONSE_TO_PLATELET_DERIVED_GROWTH_FACTOR	18	0,64	1,74	8,16E-03
GOBP_PLATELET_DERIVED_GROWTH_FACTOR_RECEPTOR_SIGNALING_PATHWAY	56	0,38	1,35	4,42E-02
GOBP_HEMATOPOIETIC_STEM_CELL_DIFFERENTIATION	88	0,42	1,63	0,00E+00
GOBP_HEMATOPOIETIC_PROGENITOR_CELL_DIFFERENTIATION	158	0,35	1,50	0,00E+00
GOBP_REGULATION_OF_HEMATOPOIETIC_PROGENITOR_CELL_DIFFERENTIATION	89	0,40	1,54	2,30E-03
GOBP_REGULATION_OF_HEMATOPOIETIC_STEM_CELL_DIFFERENTIATION	74	0,42	1,55	1,16E-02
GOBP_VASCULOGENESIS	78	0,56	2,09	0,00E+00
GOBP_REGULATION_OF_VASCULOGENESIS	16	0,76	2,04	0,00E+00
GOBP_ENDOTHELIAL_CELL_PROLIFERATION	176	0,47	2,00	0,00E+00
GOBP_NEGATIVE_REGULATION_OF_ENDOTHELIAL_CELL_PROLIFERATION	67	0,53	1,93	0,00E+00
GOBP_LYMPHANGIOGENESIS	17	0,69	1,86	0,00E+00
GOBP_VASCULAR_ENDOTHELIAL_GROWTH_FACTOR_SIGNALING_PATHWAY	45	0,52	1,77	0,00E+00
GOBP_ENDOTHELIAL_CELL_MIGRATION	257	0,39	1,77	0,00E+00
GOBP_SPROUTING_ANGIOGENESIS	164	0,41	1,74	0,00E+00
GOBP_VASCULAR_ENDOTHELIAL_GROWTH_FACTOR_RECEPTOR_SIGNALING_PATHWAY	91	0,43	1,71	0,00E+00
GOBP_POSITIVE_REGULATION_OF_ENDOTHELIAL_CELL_PROLIFERATION	101	0,43	1,67	0,00E+00
GOBP_REGULATION_OF_ENDOTHELIAL_CELL_MIGRATION	213	0,35	1,55	0,00E+00
GOBP_REGULATION_OF_ENDOTHELIAL_CELL_CHEMOTAXIS	22	0,62	1,83	4,24E-03
GOBP_POSITIVE_REGULATION_OF_ENDOTHELIAL_CELL_MIGRATION	123	0,35	1,45	9,90E-03
GOBP_CELLULAR_RESPONSE_TO_VASCULAR_ENDOTHELIAL_GROWTH_FACTOR_STIMULUS	68	0,40	1,48	1,08E-02
GOBP_BLOOD_VESSEL_ENDOTHELIAL_CELL_PROLIFERATION_INVOLVED_IN_SPROUTING_ANGIOGENESIS	47	0,43	1,49	1,94E-02
GOBP_REGULATION_OF_SPROUTING_ANGIOGENESIS	51	0,44	1,55	2,16E-02
GOBP_CELL_MIGRATION_INVOLVED_IN_SPROUTING_ANGIOGENESIS	84	0,37	1,42	2,57E-02
GOBP_ENDOTHELIAL_CELL_DEVELOPMENT	62	0,42	1,50	2,73E-02
GOBP_BLOOD_VESSEL_ENDOTHELIAL_CELL_MIGRATION	161	0,32	1,34	2,95E-02
GOBP_NEGATIVE_REGULATION_OF_VASCULAR_ENDOTHELIAL_CELL_PROLIFERATION	23	0,51	1,49	4,33E-02
GOBP_ERYTHROCYTE_DEVELOPMENT	31	0,47	1,46	4,85E-02
GOBP_T_CELL_RECEPTOR_SIGNALING_PATHWAY	194	0,30	1,31	1,27E-02
GOBP_LEUKOCYTE_MIGRATION	471	0,28	1,32	2,96E-03
GOBP_REGULATION_OF_INNATE_IMMUNE_RESPONSE	306	0,29	1,35	0,00E+00
GOBP_REGULATION_OF_NATURAL_KILLER_CELL_MEDIATED_IMMUNITY	46	0,46	1,61	2,24E-03
GOBP_NEGATIVE_REGULATION_OF_NATURAL_KILLER_CELL_MEDIATED_IMMUNITY	19	0,58	1,63	2,08E-02
GOBP_INNATE_IMMUNE_RESPONSE_ACTIVATING_SIGNAL_TRANSDUCTION	118	0,34	1,38	2,53E-02

hPSC-BC-CD144^{+}CD45^{+} *vs* EL-pre-HSCs

NAME	SIZE	ES	NES	NOM p-val
GOBP_REGULATION_OF_PLATELET_ACTIVATION	34	0,77	2,34	0,00E+00
GOBP_PLATELET_ACTIVATION	156	0,57	2,16	0,00E+00
GOBP_PLATELET_MORPHOGENESIS	20	0,77	2,09	0,00E+00
GOBP_PLATELET_DEGRANULATION	123	0,56	2,05	0,00E+00
GOBP_REGULATION_OF_PLATELET_AGGREGATION	19	0,76	2,00	0,00E+00
GOBP_PLATELET_AGGREGATION	62	0,59	1,98	0,00E+00
GOBP_NEGATIVE_REGULATION_OF_PLATELET_ACTIVATION	17	0,66	1,73	2,99E-03
GOBP_RESPONSE_TO_PLATELET_DERIVED_GROWTH_FACTOR	18	0,65	1,72	1,06E-02
GOBP_REGULATION_OF_HEMATOPOIETIC_STEM_CELL_DIFFERENTIATION	74	0,62	2,14	0,00E+00
GOBP_HEMATOPOIETIC_STEM_CELL_DIFFERENTIATION	88	0,61	2,12	0,00E+00
GOBP_REGULATION_OF_HEMATOPOIETIC_PROGENITOR_CELL_DIFFERENTIATION	89	0,60	2,09	0,00E+00
GOBP_HEMATOPOIETIC_PROGENITOR_CELL_DIFFERENTIATION	158	0,50	1,92	0,00E+00
GOBP_REGULATION_OF_HEMOPOIESIS	375	0,43	1,72	0,00E+00
GOBP_EMBRYONIC_HEMOPOIESIS	23	0,64	1,80	2,89E-03
GOBP_POSITIVE_REGULATION_OF_HEMOPOIESIS	151	0,37	1,38	1,91E-02
GOBP_VASCULAR_ENDOTHELIAL_GROWTH_FACTOR_RECEPTOR_SIGNALING_PATHWAY	91	0,50	1,79	0,00E+00
GOBP_ENDOTHELIAL_CELL_DEVELOPMENT	62	0,49	1,64	0,00E+00
GOBP_VASCULOGENESIS	78	0,44	1,54	1,21E-02
GOBP_POSITIVE_REGULATION_OF_ERYTHROCYTE_DIFFERENTIATION	31	0,61	1,83	0,00E+00
GOBP_REGULATION_OF_ERYTHROCYTE_DIFFERENTIATION	46	0,50	1,60	7,95E-03
GOBP_ERYTHROCYTE_HOMEOSTASIS	117	0,40	1,48	1,03E-02
GOBP_MEGAKARYOCYTE_DIFFERENTIATION	69	0,68	2,33	0,00E+00
GOBP_REGULATION_OF_MEGAKARYOCYTE_DIFFERENTIATION	53	0,68	2,27	0,00E+00
GOBP_MEGAKARYOCYTE_DEVELOPMENT	15	0,70	1,78	3,12E-03
GOBP_REGULATION_OF_MYELOID_LEUKOCYTE_MEDIATED_IMMUNITY	52	0,57	1,85	0,00E+00
GOBP_MYELOID_LEUKOCYTE_DIFFERENTIATION	199	0,41	1,56	1,06E-03
GOBP_MYELOID_LEUKOCYTE_MIGRATION	204	0,35	1,35	1,71E-02
GOBP_REGULATION_OF_MYELOID_LEUKOCYTE_DIFFERENTIATION	114	0,39	1,41	2,55E-02
GOBP_T_CELL_RECEPTOR_SIGNALING_PATHWAY	194	0,50	1,91	0,00E+00
GOBP_MAST_CELL_ACTIVATION	58	0,55	1,85	0,00E+00
GOBP_T_CELL_ACTIVATION	460	0,38	1,54	0,00E+00
GOBP_REGULATION_OF_MAST_CELL_ACTIVATION	39	0,57	1,79	1,34E-03
GOBP_REGULATION_OF_T_CELL_ACTIVATION	314	0,35	1,39	2,07E-03
GOBP_CD4_POSITIVE_OR_CD8_POSITIVE_ALPHA_BETA_T_CELL_LINEAGE_COMMITMENT	22	0,62	1,68	2,94E-03
GOBP_T_CELL_DIFFERENTIATION	244	0,36	1,40	4,27E-03
GOBP_T_CELL_LINEAGE_COMMITMENT	27	0,54	1,58	6,87E-03
GOBP_REGULATION_OF_LYMPHOCYTE_DIFFERENTIATION	173	0,37	1,41	1,44E-02
GOBP_POSITIVE_T_CELL_SELECTION	36	0,51	1,55	1,67E-02
GOBP_LYMPHOCYTE_COSTIMULATION	58	0,45	1,48	2,17E-02
GOBP_REGULATION_OF_LYMPHOCYTE_ACTIVATION	456	0,31	1,24	2,33E-02
GOBP_LYMPHOCYTE_APOPTOTIC_PROCESS	71	0,43	1,46	2,63E-02
GOBP_REGULATION_OF_CD8_POSITIVE_ALPHA_BETA_T_CELL_ACTIVATION	18	0,59	1,52	2,66E-02
GOBP_NEGATIVE_REGULATION_OF_T_CELL_RECEPTOR_SIGNALING_PATHWAY	22	0,56	1,54	2,71E-02
GOBP_ALPHA_BETA_T_CELL_ACTIVATION	146	0,35	1,33	3,52E-02
GOBP_REGULATION_OF_T_CELL_RECEPTOR_SIGNALING_PATHWAY	39	0,47	1,44	3,62E-02
GOBP_REGULATION_OF_LYMPHOCYTE_MIGRATION	59	0,42	1,41	3,75E-02
GOBP_REGULATION_OF_T_CELL_DIFFERENTIATION	144	0,36	1,34	3,90E-02
GOBP_T_CELL_SELECTION	48	0,46	1,46	3,94E-02
GOBP_T_CELL_MIGRATION	62	0,42	1,41	4,00E-02
GOBP_T_CELL_PROLIFERATION	188	0,35	1,32	4,54E-02
GOBP_REGULATION_OF_LEUKOCYTE_DEGRANULATION	43	0,57	1,81	1,31E-03
GOBP_REGULATION_OF_LEUKOCYTE_DIFFERENTIATION	272	0,37	1,45	3,16E-03
GOBP_REGULATION_OF_LEUKOCYTE_MIGRATION	196	0,37	1,44	5,37E-03
GOBP_LEUKOCYTE_APOPTOTIC_PROCESS	101	0,42	1,50	8,35E-03
GOBP_LEUKOCYTE_MIGRATION	471	0,32	1,30	1,02E-02
GOBP_LEUKOCYTE_CELL_CELL_ADHESION	351	0,32	1,29	1,43E-02
GOBP_REGULATION_OF_LEUKOCYTE_CHEMOTAXIS	114	0,39	1,42	1,86E-02
GOBP_LEUKOCYTE_MEDIATED_CYTOTOXICITY	107	0,40	1,45	1,89E-02
GOBP_LEUKOCYTE_PROLIFERATION	304	0,33	1,31	2,90E-02
GOBP_POSITIVE_REGULATION_OF_LEUKOCYTE_CELL_CELL_ADHESION	225	0,34	1,31	3,34E-02
GOBP_POSITIVE_REGULATION_OF_LEUKOCYTE_MIGRATION	129	0,36	1,34	3,95E-02
GOBP_REGULATION_OF_INNATE_IMMUNE_RESPONSE	306	0,52	2,08	0,00E+00
GOBP_INNATE_IMMUNE_RESPONSE_ACTIVATING_SIGNAL_TRANSDUCTION	118	0,54	1,96	0,00E+00
GOBP_REGULATION_OF_MAST_CELL_ACTIVATION_INVOLVED_IN_IMMUNE_RESPONSE	30	0,66	1,95	0,00E+00
GOBP_ACTIVATION_OF_INNATE_IMMUNE_RESPONSE	143	0,52	1,95	0,00E+00
GOBP_MAST_CELL_ACTIVATION_INVOLVED_IN_IMMUNE_RESPONSE	46	0,61	1,93	0,00E+00
GOBP_NATURAL_KILLER_CELL_MEDIATED_IMMUNITY	65	0,51	1,69	0,00E+00
GOBP_IMMUNE_RESPONSE_REGULATING_SIGNALING_PATHWAY	461	0,38	1,54	0,00E+00
GOBP_NEGATIVE_REGULATION_OF_IMMUNE_SYSTEM_PROCESS	382	0,35	1,39	1,02E-03
GOBP_NEGATIVE_REGULATION_OF_INNATE_IMMUNE_RESPONSE	64	0,48	1,63	4,83E-03
GOBP_B_CELL_ACTIVATION_INVOLVED_IN_IMMUNE_RESPONSE	74	0,46	1,58	7,39E-03
GOBP_NEGATIVE_REGULATION_OF_IMMUNE_RESPONSE	144	0,38	1,42	1,11E-02
GOBP_REGULATION_OF_NATURAL_KILLER_CELL_MEDIATED_IMMUNITY	46	0,50	1,61	1,48E-02
GOBP_SOMATIC_DIVERSIFICATION_OF_IMMUNE_RECEPTORS	69	0,43	1,49	1,49E-02
GOBP_SOMATIC_DIVERSIFICATION_OF_IMMUNE_RECEPTORS_VIA_SOMATIC_MUTATION	16	0,62	1,61	1,84E-02
GOBP_LYMPHOCYTE_ACTIVATION_INVOLVED_IN_IMMUNE_RESPONSE	184	0,36	1,37	1,86E-02
GOBP_T_HELPER_17_TYPE_IMMUNE_RESPONSE	32	0,52	1,53	1,89E-02
GOBP_REGULATION_OF_LEUKOCYTE_MEDIATED_IMMUNITY	199	0,35	1,33	2,15E-02
GOBP_SOMATIC_DIVERSIFICATION_OF_IMMUNOGLOBULINS	59	0,43	1,45	2,52E-02
GOBP_NEGATIVE_REGULATION_OF_IMMUNE_EFFECTOR_PROCESS	123	0,38	1,38	2,87E-02
GOBP_MACROPHAGE_ACTIVATION_INVOLVED_IN_IMMUNE_RESPONSE	18	0,57	1,52	2,91E-02

hPSC-BC-CD144$^+$CD45$^+$ *vs* EL-HSCs/HPs

NAME	SIZE	ES	NES
GOBP_PLATELET_ACTIVATION	156	0,71	2,86
GOBP_REGULATION_OF_PLATELET_ACTIVATION	34	0,87	2,71
GOBP_PLATELET_DEGRANULATION	123	0,68	2,67
GOBP_PLATELET_AGGREGATION	62	0,73	2,58
GOBP_REGULATION_OF_PLATELET_AGGREGATION	19	0,88	2,38
GOBP_PLATELET_MORPHOGENESIS	20	0,76	2,09
GOBP_NEGATIVE_REGULATION_OF_PLATELET_ACTIVATION	17	0,74	1,99
GOBP_REGULATION_OF_PLATELET_DERIVED_GROWTH_FACTOR_RECEPTOR_SIGNALING_PATHWAY	23	0,61	1,72
GOBP_PLATELET_DERIVED_GROWTH_FACTOR_RECEPTOR_SIGNALING_PATHWAY	56	0,42	1,45
GOBP_REGULATION_OF_HEMOPOIESIS	375	0,36	1,64
GOBP_HEMATOPOIETIC_STEM_CELL_DIFFERENTIATION	88	0,39	1,45
GOBP_DEFINITIVE_HEMOPOIESIS	18	0,59	1,61
GOBP_HEMATOPOIETIC_PROGENITOR_CELL_DIFFERENTIATION	158	0,33	1,36
GOBP_EMBRYONIC_HEMOPOIESIS	23	0,53	1,51
GOBP_POSITIVE_REGULATION_OF_HEMOPOIESIS	151	0,32	1,31
GOBP_REGULATION_OF_HEMATOPOIETIC_STEM_CELL_DIFFERENTIATION	74	0,38	1,37
GOBP_VASCULAR_ENDOTHELIAL_GROWTH_FACTOR_RECEPTOR_SIGNALING_PATHWAY	91	0,58	2,14
GOBP_REGULATION_OF_ENDOTHELIAL_CELL_CHEMOTAXIS	22	0,73	2,09
GOBP_ENDOTHELIAL_CELL_DEVELOPMENT	62	0,54	1,90
GOBP_VASCULOGENESIS	78	0,48	1,76
GOBP_ENDOTHELIAL_CELL_MIGRATION	257	0,40	1,74
GOBP_POSITIVE_REGULATION_OF_ENDOTHELIAL_CELL_MIGRATION	123	0,40	1,59
GOBP_BLOOD_VESSEL_ENDOTHELIAL_CELL_MIGRATION	161	0,36	1,50
GOBP_ENDOTHELIAL_CELL_CHEMOTAXIS	29	0,58	1,73
GOBP_ENDOTHELIAL_CELL_PROLIFERATION	176	0,37	1,53
GOBP_REGULATION_OF_ENDOTHELIAL_CELL_MIGRATION	213	0,35	1,48
GOBP_ESTABLISHMENT_OF_ENDOTHELIAL_BARRIER	45	0,50	1,63
GOBP_REGULATION_OF_SPROUTING_ANGIOGENESIS	51	0,45	1,54
GOBP_POSITIVE_REGULATION_OF_SPROUTING_ANGIOGENESIS	26	0,55	1,62
GOBP_MEGAKARYOCYTE_DIFFERENTIATION	69	0,57	2,05
GOBP_REGULATION_OF_MEGAKARYOCYTE_DIFFERENTIATION	53	0,56	1,92
GOBP_MEGAKARYOCYTE_DEVELOPMENT	15	0,67	1,72
GOBP_REGULATION_OF_MYELOID_LEUKOCYTE_MEDIATED_IMMUNITY	52	0,67	2,25
GOBP_MYELOID_LEUKOCYTE_MIGRATION	204	0,47	1,98
GOBP_POSITIVE_REGULATION_OF_MACROPHAGE_CHEMOTAXIS	17	0,75	1,95
GOBP_GRANULOCYTE_CHEMOTAXIS	118	0,46	1,81
GOBP_MYELOID_LEUKOCYTE_DIFFERENTIATION	199	0,42	1,77
GOBP_REGULATION_OF_GRANULOCYTE_CHEMOTAXIS	48	0,52	1,74
GOBP_NEUTROPHIL_CHEMOTAXIS	97	0,44	1,64
GOBP_REGULATION_OF_MYELOID_LEUKOCYTE_DIFFERENTIATION	114	0,42	1,61
GOBP_REGULATION_OF_MACROPHAGE_CHEMOTAXIS	27	0,59	1,74
GOBP_POSITIVE_REGULATION_OF_MYELOID_LEUKOCYTE_DIFFERENTIATION	57	0,45	1,53
GOBP_POSITIVE_REGULATION_OF_MYELOID_LEUKOCYTE_MEDIATED_IMMUNITY	17	0,60	1,61
GOBP_MACROPHAGE_CHEMOTAXIS	37	0,50	1,60
GOBP_LYMPHOCYTE_COSTIMULATION	58	0,51	1,76
GOBP_REGULATION_OF_LYMPHOCYTE_ACTIVATION	456	0,29	1,33
GOBP_REGULATION_OF_LYMPHOCYTE_MIGRATION	59	0,44	1,55
GOBP_NEGATIVE_REGULATION_OF_LYMPHOCYTE_MEDIATED_IMMUNITY	45	0,45	1,46
GOBP_LYMPHOCYTE_MEDIATED_IMMUNITY	305	0,28	1,26
GOBP_LYMPHOCYTE_MIGRATION	111	0,35	1,35
GOBP_REGULATION_OF_LEUKOCYTE_DEGRANULATION	43	0,70	2,28
GOBP_LEUKOCYTE_MIGRATION	471	0,45	2,06
GOBP_REGULATION_OF_LEUKOCYTE_MIGRATION	196	0,47	1,98
GOBP_POSITIVE_REGULATION_OF_LEUKOCYTE_MIGRATION	129	0,49	1,94
GOBP_NEGATIVE_REGULATION_OF_LEUKOCYTE_MEDIATED_IMMUNITY	53	0,55	1,86
GOBP_REGULATION_OF_LEUKOCYTE_CHEMOTAXIS	114	0,47	1,85
GOBP_POSITIVE_REGULATION_OF_LEUKOCYTE_CHEMOTAXIS	89	0,48	1,80
GOBP_LEUKOCYTE_CHEMOTAXIS	213	0,42	1,80
GOBP_LEUKOCYTE_MEDIATED_CYTOTOXICITY	107	0,46	1,74
GOBP_REGULATION_OF_LEUKOCYTE_MEDIATED_IMMUNITY	199	0,41	1,74
GOBP_LEUKOCYTE_CELL_CELL_ADHESION	351	0,34	1,51
GOBP_REGULATION_OF_LEUKOCYTE_DIFFERENTIATION	272	0,32	1,37
GOBP_POSITIVE_REGULATION_OF_LEUKOCYTE_CELL_CELL_ADHESION	225	0,34	1,45
GOBP_LEUKOCYTE_PROLIFERATION	304	0,32	1,42
GOBP_POSITIVE_REGULATION_OF_LEUKOCYTE_DEGRANULATION	23	0,61	1,76
GOBP_REGULATION_OF_LEUKOCYTE_MEDIATED_CYTOTOXICITY	75	0,43	1,55
GOBP_NEGATIVE_REGULATION_OF_LEUKOCYTE_MEDIATED_CYTOTOXICITY	22	0,61	1,72
GOBP_LEUKOCYTE_APOPTOTIC_PROCESS	101	0,40	1,52
GOBP_POSITIVE_REGULATION_OF_LEUKOCYTE_PROLIFERATION	144	0,35	1,40
GOBP_REGULATION_OF_LEUKOCYTE_PROLIFERATION	233	0,33	1,39
GOBP_NEGATIVE_REGULATION_OF_LEUKOCYTE_MIGRATION	40	0,44	1,44
GOBP_NATURAL_KILLER_CELL_MEDIATED_IMMUNITY	65	0,64	2,25
GOBP_REGULATION_OF_NATURAL_KILLER_CELL_MEDIATED_IMMUNITY	46	0,64	2,18
GOBP_REGULATION_OF_MAST_CELL_ACTIVATION_INVOLVED_IN_IMMUNE_RESPONSE	30	0,71	2,14
GOBP_MAST_CELL_ACTIVATION_INVOLVED_IN_IMMUNE_RESPONSE	46	0,63	2,08
GOBP_POSITIVE_REGULATION_OF_NATURAL_KILLER_CELL_MEDIATED_IMMUNITY	29	0,67	2,04
GOBP_REGULATION_OF_INNATE_IMMUNE_RESPONSE	306	0,45	1,97
GOBP_NEGATIVE_REGULATION_OF_NATURAL_KILLER_CELL_MEDIATED_IMMUNITY	19	0,70	1,92
GOBP_INNATE_IMMUNE_RESPONSE_ACTIVATING_SIGNAL_TRANSDUCTION	118	0,46	1,80
GOBP_ACTIVATION_OF_INNATE_IMMUNE_RESPONSE	143	0,43	1,71
GOBP_IMMUNE_RESPONSE_REGULATING_SIGNALING_PATHWAY	461	0,37	1,70
GOBP_NEGATIVE_REGULATION_OF_IMMUNE_SYSTEM_PROCESS	382	0,36	1,63
GOBP_NEGATIVE_REGULATION_OF_IMMUNE_EFFECTOR_PROCESS	123	0,40	1,58
GOBP_REGULATION_OF_IMMUNE_EFFECTOR_PROCESS	430	0,34	1,54
GOBP_NEGATIVE_REGULATION_OF_INNATE_IMMUNE_RESPONSE	64	0,48	1,68
GOBP_NEGATIVE_REGULATION_OF_IMMUNE_RESPONSE	144	0,37	1,51
GOBP_POSITIVE_REGULATION_OF_IMMUNE_EFFECTOR_PROCESS	210	0,35	1,47
GOBP_MACROPHAGE_ACTIVATION_INVOLVED_IN_IMMUNE_RESPONSE	18	0,63	1,67
GOBP_NATURAL_KILLER_CELL_ACTIVATION_INVOLVED_IN_IMMUNE_RESPONSE	31	0,48	1,48

hPSC-ECs *vs* EL-ECs

NAME	SIZE	ES	NES	NOM p-val
GOBP_PLATELET_AGGREGATION	62	0,46	1,58	7,08E-03
GOBP_PLATELET_DEGRANULATION	123	0,37	1,40	1,60E-02
GOBP_REGULATION_OF_PLATELET_DERIVED_GROWTH_FACTOR_RECEPTOR_SIGNALING_PATHWAY	23	0,58	1,60	1,67E-02
GOBP_PLATELET_DERIVED_GROWTH_FACTOR_RECEPTOR_SIGNALING_PATHWAY	56	0,45	1,50	2,12E-02
GOBP_PLATELET_ACTIVATION	156	0,33	1,30	3,68E-02
GOBP_DNA_TEMPLATED_TRANSCRIPTION_INITIATION	228	0,31	1,25	3,72E-02
GOBP_NEGATIVE_REGULATION_OF_PLATELET_ACTIVATION	17	0,58	1,48	4,68E-02
GOBP_HEMATOPOIETIC_PROGENITOR_CELL_DIFFERENTIATION	158	0,46	1,81	0,00E+00
GOBP_HEMATOPOIETIC_STEM_CELL_DIFFERENTIATION	88	0,48	1,73	0,00E+00
GOBP_REGULATION_OF_HEMATOPOIETIC_PROGENITOR_CELL_DIFFERENTIATION	89	0,46	1,66	0,00E+00
GOBP_REGULATION_OF_HEMATOPOIETIC_STEM_CELL_DIFFERENTIATION	74	0,46	1,61	1,77E-03
GOBP_VASCULAR_ENDOTHELIAL_GROWTH_FACTOR_RECEPTOR_SIGNALING_PATHWAY	91	0,41	1,50	9,29E-03
GOBP_ENDOTHELIAL_CELL_DEVELOPMENT	62	0,44	1,49	1,77E-02
GOBP_ENDOTHELIAL_CELL_MIGRATION	257	0,30	1,23	4,48E-02
GOBP_MYELOID_LEUKOCYTE_MIGRATION	204	0,31	1,27	2,86E-02
GOBP_T_CELL_RECEPTOR_SIGNALING_PATHWAY	194	0,35	1,41	3,63E-03
GOBP_REGULATION_OF_T_CELL_RECEPTOR_SIGNALING_PATHWAY	39	0,46	1,43	3,88E-02
GOBP_NEGATIVE_REGULATION_OF_T_CELL_RECEPTOR_SIGNALING_PATHWAY	22	0,54	1,48	4,58E-02
GOBP_POSITIVE_REGULATION_OF_LEUKOCYTE_DEGRANULATION	23	0,62	1,71	8,99E-03
GOBP_LEUKOCYTE_MIGRATION	471	0,29	1,28	1,66E-02
GOBP_POSITIVE_REGULATION_OF_LEUKOCYTE_MIGRATION	129	0,34	1,30	4,89E-02
GOBP_INNATE_IMMUNE_RESPONSE_ACTIVATING_SIGNAL_TRANSDUCTION	118	0,37	1,39	2,41E-02
GOBP_IMMUNE_RESPONSE_REGULATING_SIGNALING_PATHWAY	461	0,28	1,25	2,48E-02
